# Heterolysis of Dihydrogen by Nucleophilic Calcium Alkyls

**DOI:** 10.1002/anie.201809833

**Published:** 2018-10-26

**Authors:** Andrew S. S. Wilson, Chiara Dinoi, Michael S. Hill, Mary F. Mahon, Laurent Maron

**Affiliations:** ^1^ Department of Chemistry University of Bath Bath BA2 7AY UK; ^2^ Université de Toulouse et CNRS INSA UPS, UMR 5215 LPCNO 135 Avenue de Rangueil 31077 Toulouse France

**Keywords:** calcium, catalysis, density functional theory, hydrogenation, main group chemistry

## Abstract

β‐Diketiminato (BDI) calcium alkyl derivatives undergo hydrogenolysis with H_2_ to regenerate [(BDI)CaH]_2_, allowing the catalytic hydrogenation of a wide range of 1‐alkenes and norbornene under very mild conditions (2 bar H_2_, 298 K). The reactions are deduced to take place with the retention of the dimeric structures of the calcium hydrido‐alkyl and alkyl intermediates via a well‐defined sequence of Ca−H/C=C insertion and Ca−C hydrogenation events. This latter deduction is strongly supported by DFT calculations (B3PW91) performed on the 1‐hexene/H_2_ system, which also indicates that the hydrogenation transition states display features which discriminate them from a classical σ‐bond metathesis mechanism. In particular, NBO analysis identifies a strong second order interaction between the filled α‐methylene sp^3^ orbital of the n‐hexyl chain and the σ* orbital of the H_2_ molecule, signifying that the H−H bond is broken by what is effectively the nucleophilic displacement of hydride by the organic substituent.

The catalytic hydrogenation of alkenes and alkynes by mid to late transition metal complexes has been developed to a high degree of sophistication and is typically predicated on the cooperative binding and activation of both unsaturated hydrocarbons and H_2_ at a redox‐active metal center.^1^, ^2^, ^3^, ^4^, ^5^, ^6^, ^7^, ^8^, ^9^ Albeit less well developed, similar catalysis was achieved by group 3 and organolanthanide complexes more than a quarter of a century ago.^10^, ^11^, ^12^, ^13^, ^14^ In these latter cases, a d^0^ electron configuration mitigates against redox‐based reactivity and turnover is derived from alkene insertion into a polarised metal‐hydrogen bond to form an alkyl intermediate, which is itself reactive toward H_2_ through σ‐bond metathesis to release the hydrocarbon and regenerate the active hydride catalyst.

Reports of analogous catalysis by main group (s‐ or p‐block) compounds are more recent in origin and largely restricted to conjugated C=C and C≡C bonded substrates.^15^, ^16^ Reactivity is again, however, dependent on the intermediacy of highly reactive hydride derivatives. While these species may be formed by frustrated Lewis pair (FLP)‐derived heterolysis of the dihydrogen H−H σ bond,^17^ a seminal report in s‐block chemistry was provided by Harder's molecular β‐diketiminato calcium hydride complex, [(BDI)CaH(THF)]_2_ (BDI=HC{(Me)CN‐2,6‐*i*‐Pr_2_C_6_H_3_}_2_; **1**).

Like the aforementioned organolanthanides, the calcium center of **1** possesses a formal d^0^ configuration and was found to engage in polarized insertion reactivity with a variety of unsaturated substrates.^18^, ^19^ Although compound **1** helped stimulate widespread interest in the isolation of heavy s‐block hydrides, its reactivity with C=C bonds was limited to the conjugated and more activated alkenes, 1,3‐cyclohexadiene,^19^ myrcene, and 1,1‐diphenylethene.^20^ Addition of hydrogen (20 bar, 60 °C) to THF solutions of the resultant organometallic compounds also completely regenerated the parent hydride (**1**), albeit incorporation of this hydrogenation reactivity into a catalytic regime was similarly limited to these activated alkenes. While related hydrogenation catalysis has also been extended to the reduction of more polarized imines,^21^ the calcium‐catalyzed hydrogenation of unactivated terminal alkenes has only been described very recently. Okuda and co‐workers have reported that the catalytic hydrogenation of linear alkenes, including 1‐hexene and 1‐octene, can be mediated by the dicationic calcium hydride [Ca_2_H_2_(Me_4_TACD)_2_][BAr_4_]_2_ (Me_4_TACD=1,4,7,10‐tetramethyl‐1,4,7,10‐tetraazacyclododecane; Ar=C_6_H_4_‐4‐^*t*^Bu) (**2**) in THF. The catalysis was proposed to proceed via linear calcium σ‐*n*‐alkyls that either rapidly cleave dihydrogen or themselves undergo β‐hydride elimination to reform the active calcium hydride species (Scheme 1).^22^ The necessary calcium *n*‐alkyl intermediates could only be inferred, however, and the nature of the hydrogenolysis step was consequently unamenable to direct study.

**Scheme 1:**
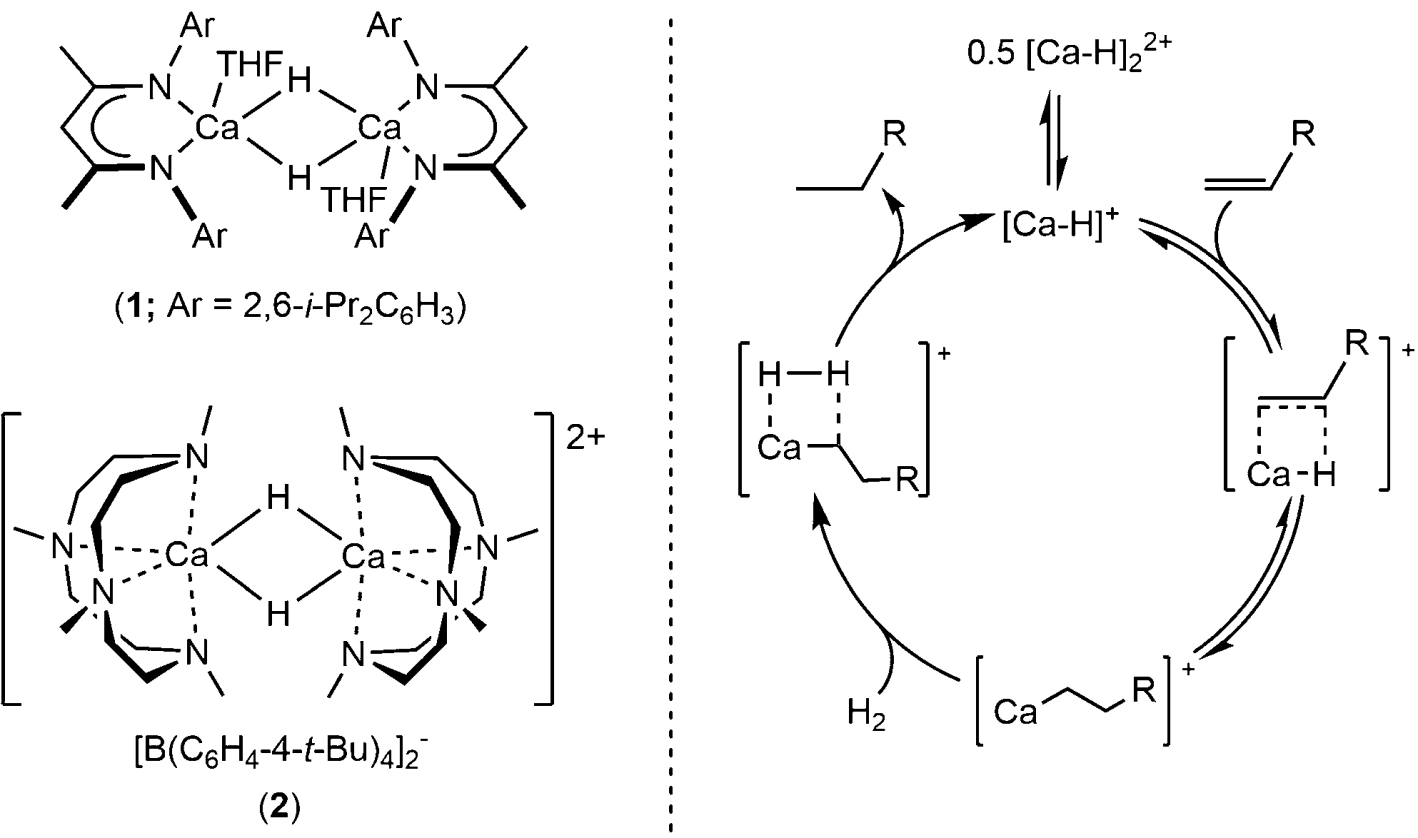
Compounds **1** and **2** and Okuda's proposed catalytic cycle for the hydrogenation of terminal alkenes by **2**.

In contrast, we have recently reported that a non‐THF solvated analogue of compound **1**, compound **3**, reacts readily with the terminal alkenes, ethene, 1‐butene and 1‐hexene to provide the corresponding calcium ethyl, *n*‐butyl and *n*‐hexyl derivatives (**7**–**9**, Scheme 2).^23^ The formation of the dimeric compounds **7**–**9** was observed to ensue in a stepwise fashion through the intermediacy of mixed hydride‐alkyl species (**4**–**6**). Although **7**–**9** were stable in aliphatic solvents, solutions in benzene at even mildly elevated temperatures (ca. 50 °C) were found effect the unprecedented nucleophilic alkylation of a C−D bond of the C_6_D_6_ solvent. In this contribution, we further examine the reactivity of these heteroleptic calcium alkyls with dihydrogen and report our observations with regard to the activity of compound **3** for the catalytic hydrogenation of alkenes.

**Scheme 2:**
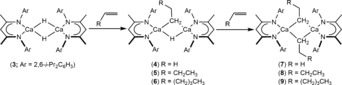
Synthesis of compounds **4**–**9**.

An atmosphere of dihydrogen (2 bar) was added to a C_6_D_6_ solution of the calcium *n*‐hexyl derivative (**9**). Immediate analysis of the resultant ^1^H NMR spectrum confirmed that the solution comprised primarily a mixture of unreacted **9** (*δ*=4.69 ppm) and hydrogen (*δ*=4.46 ppm). Small quantities of the hydride (**3**) and the dicalcium *n*‐hexyl‐hydride (**6**), however, were also detected by their respective BDI methine resonances at *δ*=4.83 and 4.77 ppm. After storage at room temperature for 24 hours, the reaction was observed to contain a mixture of **3** and **9**, whilst no significant increase in the intensity of the signals associated with the dicalcium hexyl‐hydride intermediate (**6**) was observed. The apparent steady‐state concentration of the dicalcium hexyl‐hydride suggested hydrogenation was occurring via a two‐step process, in which **6** is formed but more rapidly converted to the hydride (**3**). After 48 hours at room temperature, the complete conversion of **9** to **3** and the quantitative formation of *n*‐hexane were confirmed by the emergence of the BDI methine and hydride resonances of **3** (*δ*=4.83 and 4.27 ppm) and the triplet of *n*‐hexane (*δ*=0.89 ppm), which displayed a 1:1:6 ratio of intensities in the resultant ^1^H NMR spectrum. Although slower due to a likely primary kinetic isotope effect, analogous addition of D_2_ to a C_6_D_6_ solution of **9** similarly afforded **3**‐*d_2_* and CH_2_D(CH_2_)_3_CH_3_, which could be clearly detected by the emergence of its BDI methine signal at *δ=*4.83 ppm in the resultant ^1^H NMR spectrum and by the relevant deuterated Ca−D and CH_2_D(CH_2_)_3_CH_3_ signals (*δ*=4.30 and 0.85 ppm) in the corresponding ^2^H NMR spectrum.

An initial catalytic reaction mediated by 10 mol % of **3** with 1‐hexene under a dihydrogen atmosphere (2 bar) in C_6_D_6_ provided slow turnover at room temperature to hexane over the duration of three weeks as confirmed by the emergence of the diagnostic triplet signal of hexane at *δ*=0.89 in the resultant ^1^H NMR spectrum. Encouraged by this successful reactivity, the catalytic hydrogenation of a range of alkenes by **3** was investigated. The results of this study are summarized in Table 1.

**Table 1:** Catalytic hydrogenation of alkenes with **3** (10 mol %, 25 °C, C_6_D_6_).

Entry	Substrate	Product	*t* [days]	Conv.^[a]^ [%]	TOF [h^−1^]
1	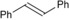	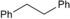	21	99	0.02
2	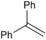	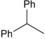	21	99	0.02
3	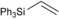	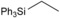	7	65	0.04
4	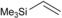	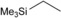	7	65	0.04
5	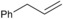	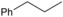	14	99	0.03
6	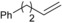	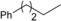	14	99	0.03
7	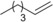	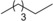	21	99	0.02
8	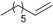	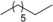	21	99	0.02
9	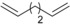	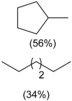	14	95^[b]^	0.03
10	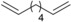	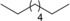	21	90^[c]^	0.02
11	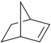	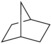	14	69	0.02

[a] Determined by ^1^H NMR spectroscopy by integration against C_6_Me_6_ as an internal standard; [b] ca. 5 % 2‐hexene also observed; [c] ca. 10 % 2‐octene also observed.

All substrates proceeded with good to excellent conversions (ca. ≥65 %) at room temperature, albeit with similar turnover frequencies (0.02–0.04 h^−1^) and prolonged reactions times. This latter observation is interpreted to indicate that the slow rate of catalysis is a result of the limited solubility and availability of hydrogen in C_6_D_6_ under the conditions of the experiments (2 bar) rather than any variation in the nature of the substrates. At elevated temperatures, hydrogenation catalysis by **3** was suppressed by facile redistribution of the ligand system to the catalytically inactive homoleptic β‐diketiminate species, [(BDI)_2_Ca].^24^, ^25^ Consistent with the lower conversions, alongside the desired alkanes, unidentified products were observed in the ^1^H NMR spectra throughout the hydrogenation of vinyltrimethylsilane, triphenyl(vinyl)silane, and norbornene (Entries 3, 4 and 11). Notably, a significant quantity of the cyclized alkane, methylcyclopentane (56 %) was observed to form during the hydrogenation of 1,5‐hexadiene (Entry 9), which suggested the intramolecular cyclization is competitive with hydrogenation. In contrast, markedly less methylcyclopentane (4 %) was formed during Okuda's hydrogenation of 1,5‐hexadiene catalyzed by **2**.^22^ To provide further insight into this observation, three equivalents of 1,5‐hexadiene were added to a C_6_D_6_ solution of compound **3**. Monitoring of the reaction at room temperature for 24 hours revealed the production of a mixture of unidentified products in addition to a dicalcium cyclopentylmethyl‐hydride species (**10**) and the corresponding dicalcium‐dicyclopentylmethyl derivative (**11**), identified by the emergence of BDI methine and upfield methylene doublet signals with relative intensities of 1:2 at *δ*=4.72 and −0.55 ppm, respectively. Despite the mixture of products formed, single crystals deposited from the reaction mixture and the resultant X‐ray diffraction analysis allowed the structure of **11** to be confirmed as the result of facile carbocalciation and 5‐*exo*‐trig cyclization (Figure 1).

**Figure 1:**
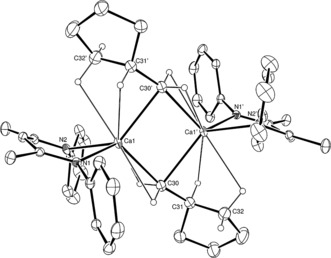
ORTEP representation of compound **11** with thermal ellipsoids at 25 % probability. *Iso*‐propyl groups, and hydrogen atoms except those attached to C30—C32 have been removed for clarity. Selected bond lengths (Å) angles (°): Ca1−N1 2.3541(12), Ca1−N2 2.3391(12), Ca1−C30 2.5164(18), Ca1−C30′ 2.5759(18), Ca1⋅⋅⋅C31′ 2.915(2), N2−Ca1−N1 83.16(4), Ca1−C30−Ca1′ 82.03(5); Primed labelled atoms related to those in the asymmetric unit by the 3/2−*x*, 3/2−*y*, 1−*z* symmetry operator.

The hydrogenation of the activated alkenes, 1,1‐diphenylethylene, styrene and α‐methylstyrene catalyzed by compound **1** was reported to occur over a shorter timeframe (17–25 hours) than the corresponding results shown in Table 1. This previous study, however, was performed with a greater pressure of dihydrogen (20 bar) and at an elevated temperature (60 °C) and provided significantly lower selectivity for conversion to the corresponding alkanes, 1,1‐diphenylethane (49 %), ethylbenzene (99 % but 19 % oligomers) and isopropylbenzene (60 %).^20^ Okuda's recent study of catalytic alkene hydrogenation with the cationic calcium hydride, [Ca_2_H_2_(Me_4_TACD)_2_][B(C_6_H_4_‐4‐^*t*^Bu)_4_]_2_ (**2**) significantly expanded this substrate scope to include less activated terminal alkenes.^22^ Hydrogenation was also observed to ensue at a faster rate (16–36 hours) and to provide conversions of 92–98 % under a 1 bar atmosphere of dihydrogen, albeit these experiments were also performed at 60 °C in [D8]THF. Catalysis performed by **2**, however, also ensued without any observable formation of the necessary alkyl intermediates, an observation which was ascribed to their instantaneous consumption either by hydrogenolysis or competitive β‐hydride elimination (Scheme 1). We have observed only limited potential for β‐hydride elimination from monitoring of solutions of compound **9** alone or a 1:1 mixture of compounds **3** and **9**. This latter solution equilibrated to an approximate 1:1:0.7 mixture of **3**:**9**:**6** over a 24 hour period but evidenced no elimination of 1‐hexene. Similarly, monitoring of a C_6_D_6_ solution of the calcium *n*‐hexyl (**9**) under 1 atmosphere of 1‐butene by ^1^H NMR spectroscopy demonstrated that the 1‐butene was only very slowly consumed over the course of four weeks. This process occurred with the simultaneous emergence of a new alkene multiplet at *δ*=5.48–5.53 ppm and an aliphatic doublet signal at 1.55 ppm, which were assigned to the generation of the internal alkene, 2‐butene. Although not investigated further, the formation of 2‐butene is proposed to have arisen from an effective chain walking mechanism involving the β‐hydride elimination reaction of **9**, 2,1‐insertion of a molecule of 1‐butene into the reformed Ca−H bond and a subsequent β‐hydride elimination reaction.

On the basis of these observations, the mechanism shown in Scheme 3 may be invoked for the catalytic hydrogenation of alkenes with compound **3**. The mechanism is again predicated upon a cascade of polarized insertion reactions in conjunction with irreversible hydrogenolysis and occurs with the maintenance of dimeric calcium species throughout the catalysis. The stoichiometric reactivity studies described above indicate that insertion of alkenes occurs in a stepwise fashion, whereupon the resultant dicalcium alkyl‐hydride intermediates are produced in an effective steady state throughout the course of catalysis. Although these latter species have the potential to undergo a second alkene insertion, we suggest that turnover is largely based on their interception and hydrogenolysis by H_2_.

**Scheme 3:**
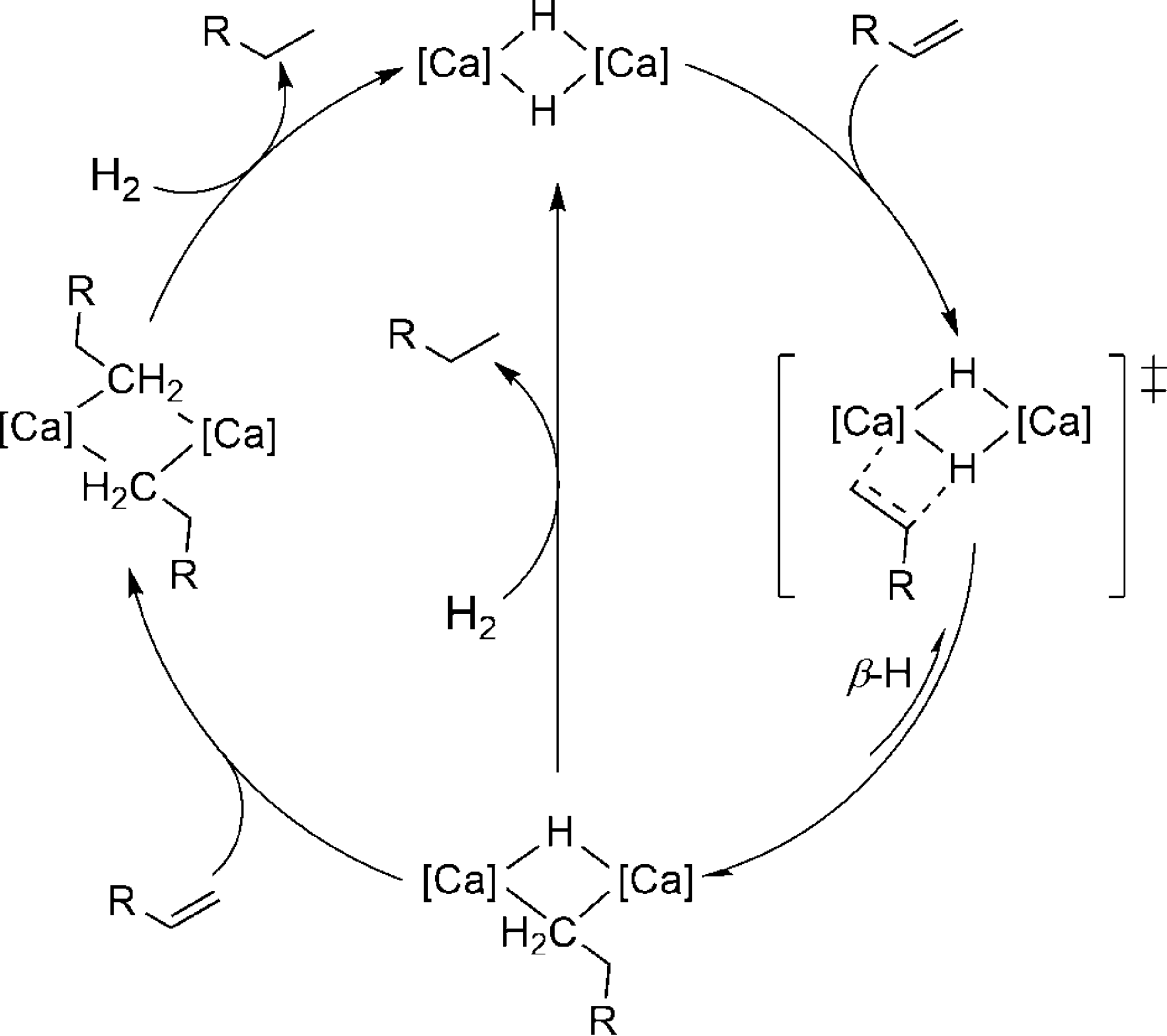
Proposed mechanism for catalytic hydrogenation of alkenes with **3**.

Further insight into this proposed mechanism was provided by density functional theory (DFT) calculations carried out with the same computational approach (B3PW91, see SI) previously used to describe the stepwise formation of the dicalcium di‐*n*‐hexyl derivative (**9**), through the reaction of compound **3** with 1‐hexene.^23^ The first 1‐hexene insertion with compound **3** was described in our previous report and the associated barrier yielding the dicalcium *n*‐hexyl‐hydride complex **C** (**6**) is 20.1 kcal mol^−1^. From **C** (**6**), either hydrogenation by H_2_ (left part of Figure 2) or further 1‐hexene insertion (right part of Figure 2) was considered computationally. It should first be noted that both reactions occur on the bimetallic species as the hydride dimer dissociation was computed to be endothermic by 40.4 kcal mol^−1^ (Figure S20 in the Supporting Information).

**Figure 2:**
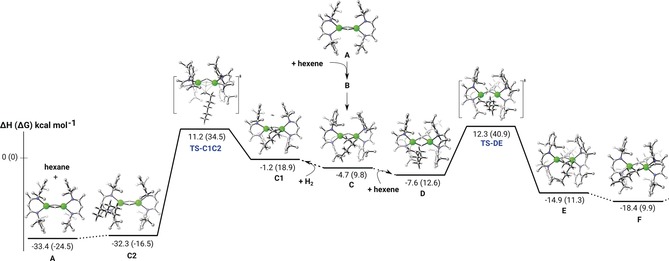
DFT (B3PW91) computed enthalpy reaction profile at room temperature for the direct hydrogenation vs. the 1‐hexene insertion of the dicalcium *n*‐hexyl‐hydride complex **C** (**6**).

The difference between the barrier heights associated with the direct hydrogenation (15.9 kcal mol^−1^) of **C** (**6**) and 1‐hexene insertion (19.9 kcal mol^−1^) is 4.0 kcal mol^−1^, indicating a marginal preference for the direct hydrogenation. Within the precision of the method, however, the possibility of 1‐hexene insertion cannot be ruled out completely. The complete hydrogenation subsequent to the second 1‐hexene insertion, that is, from the dimeric *n*‐hexyl complex **F** (**9**), is also reported on Figure S21. The two barriers found for the hydrogenation from **F** (**9**) (17.3 and 19.1 kcal mol^−1^) are of a similar magnitude as the barrier for the hydrogenation from **C** (**6**). The hydrogenation, therefore, may be deduced to be similarly kinetically accessible for both possible pathways.

The hydrogenation transition states are particularly notable and display features which discriminate them from a classical σ‐bond metathesis. Examination of the relevant molecular orbitals and the NBO analysis of the hydrogenation of the *n*‐hexyl‐hydride species **C** (**6**) is clearly indicative of a heterolytic cleavage of the H_2_ molecule. The HOMO−2 (Figure S22) involves the filled *sp* ^3^ orbital of the *n*‐hexyl chain, the H_2_ σ* and a 3*d* orbital of both Ca centers. The involvement of 3d orbitals on Ca has recently been invoked as a potentially significant factor in the structural and reaction chemistry of organocalcium species.^26^, ^27^, ^28^ This analysis indicates, therefore, that the hydrogen atom interacting with the *n*‐hexyl unit is better viewed as a proton, whereas the hydrogen atom interacting with the two Ca centers is much more hydridic in nature. This interpretation is further corroborated by the corresponding NBO analysis in which a strong second order interaction is observed between the filled α‐methylene sp^3^ orbital of the *n*‐hexyl chain and the σ* orbital of the H_2_ molecule, signifying that the H−H bond is broken by what is effectively the nucleophilic displacement of hydride by the organic substituent. This electron density is then redistributed via by a less strong second order interaction between the σ*_H−H_ orbital and the two Ca atoms. The highly polarized nature of this process is underscored by the NPA charge analysis, which indicates a charge of −1.0 on the α‐methylene carbon of the *n*‐hexyl chain, charges of +1.50 and +1.43 for the two Ca centers and charges of −0.23 and +0.03 for the two H_2_ hydrogen atoms. An effectively identical interpretation also emerges for the hydrogenation TS from the dicalcium di‐*n*‐hexyl species **F** (**9**) (Figure S23).

In conclusion, hydrogenation of the Ca−C bonds of β‐diketiminato calcium alkyls occurs through retention of the dimeric calcium alkyl and hydrido‐alkyl species allowing the catalytic hydrogenation of a wide range of olefinic substrates under mild conditions. The hydrogenolysis step ensues through an unprecedented bimetallic nucleophilic pathway in which the H−H bond is cleaved heterolytically, that was so far only reported on monometallic species.^29^, ^30^ Although largely a consequence of the highly polarized nature of the Ca−C bonding, more generally these observations underscore the view that group 2 organometallic reactivity is not simply “lanthanide mimetic”, but displays features that are unique to each of the available elements. We are continuing to examine these possibilities.

## Conflict of interest

The authors declare no conflict of interest.

## Supporting information

As a service to our authors and readers, this journal provides supporting information supplied by the authors. Such materials are peer reviewed and may be re‐organized for online delivery, but are not copy‐edited or typeset. Technical support issues arising from supporting information (other than missing files) should be addressed to the authors.

Supplementary
