# Supplementary material for: Heterolysis of Dihydrogen by Nucleophilic Calcium Alkyls
Source: Angew Chem Int Ed Engl. 2018 Oct 26;57(47):15500–4. doi: 10.1002/anie.201809833 (PMC6282996; doi:10.1002/anie.201809833)
Supplement: Supplementary file 1 — Supplementary [file ANIE-57-15500-s001.pdf]

Angewandte Chemie
Eine Zeitschrift der Gesellschaft Deutscher Chemiker

Supporting Information

Heterolysis of Dihydrogen by Nucleophilic Calcium Alkyls

Andrew S. S. Wilson, Chiara Dinoi, Michael S. Hill, Mary F. Mahon, and Laurent Maron**

anie_201809833_sm_miscellaneous_information.pdf

Supplementary Methods

General considerations and starting materials.

All manipulations were carried out using standard Schlenk line and glovebox techniques under an inert atmosphere of argon. NMR experiments were conducted in J Young tap NMR tubes prepared and sealed in a Glovebox. NMR spectra were collected on a Bruker AV300 spectrometer operating at 300.2 MHz (^{1}H), 75.5 MHz (^{13}C) or an Agilent ProPulse spectrometer operating at 500 MHz (^{1}H), 126 MHz (^{13}C). The spectra were referenced relative to residual protio solvent resonances. Solvents (Toluene, Hexane, Et_2O) were dried by passage through a commercially available (Innovative Technologies) solvent purification system, under nitrogen and stored in ampoules over 4Å molecular sieves. C_6D_6 was purchased from Sigma-Aldrich Corp., dried over a potassium mirror before vacuum distilling under argon and storing over molecular sieves. Calcium iodide (99.95%) and phenylsilane (97%) were purchased from Sigma-Aldrich Corp. and used without further purification. Diphenylacetylene (98%) and *trans*-stilbene (96%) were purchased from Sigma-Aldrich Corp., recrystalised from ethanol and dried under high vacuum. Liquid alkenes; 1,1-diphenylethene (97 %), 3,3-dimethyl-1-butene (97%), 1-hexene (97%), 1,5-hexadiene (97%), 1-octene (98%), 1,7-octadiene (98%), allylbenzene (98%) and 4-phenyl-1-butene (99%) were purchased from Sigma-Aldrich Corp., dried over calcium hydride and distilled under argon before use. But-1-ene was purchased from Fluorochem Ltd. and used without further purification. Hydrogen (99.99%) was purchased from BOC and passed through an oxygen/moisture trap before each use. [(BDI)CaH] (BDI = HC{(MeCN-2,6-*i*$Pr_2C_6H_3$}$_2$) (**3**) was synthesized by a literature procedure.[1]

Synthetic, spectroscopic and analytical data for new compounds

[(BDI)Ca(CH{CH_2}$_4$)]$_2$ (11)

In a J Youngs NMR tube, C_6D_6 (0.5 mL) was added to a mixture of **3** (30 mg, 0.065 mmol) and 1,5-hexadiene (23.3 µl, 0.20 mmol). Over the course of one day at room temperature colourless material containing single crystals of compound **11** suitable for X-ray diffraction analysis deposited (1 mg).

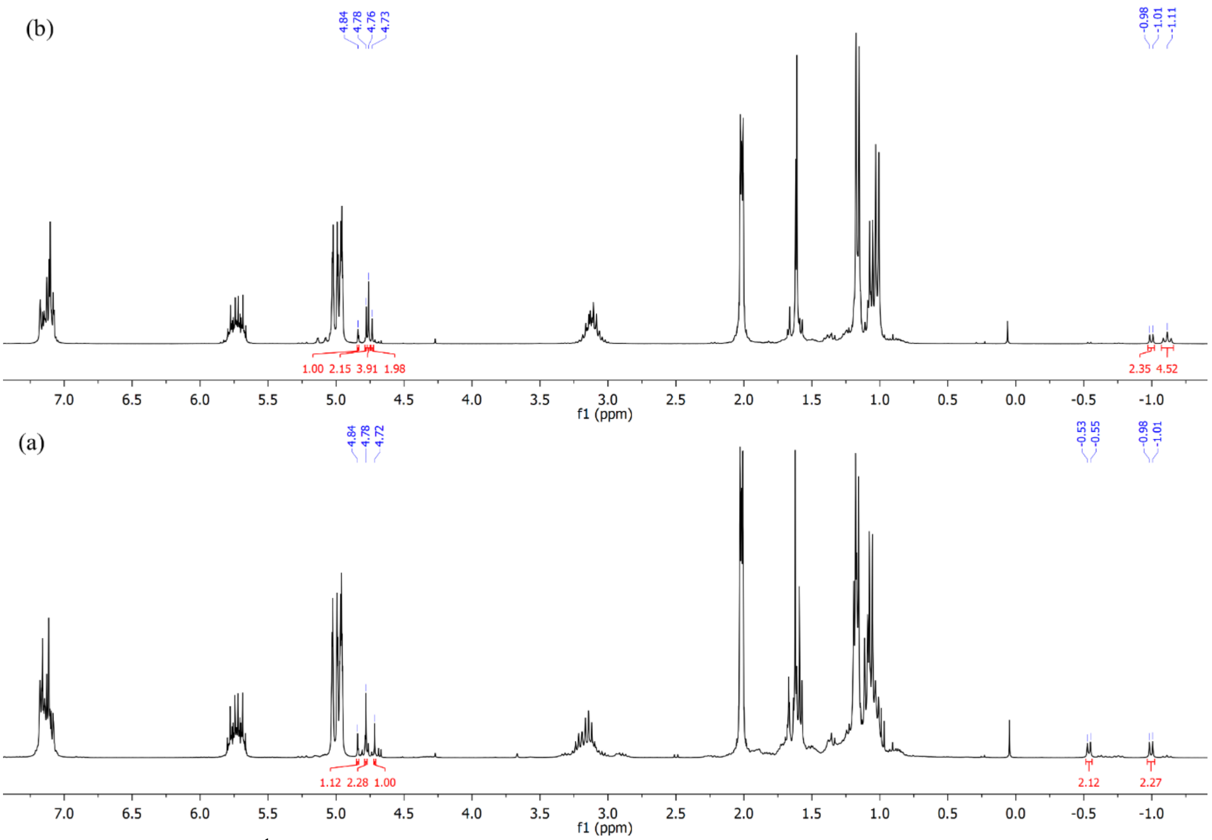

Figure S1: Stacked ^{1}H NMR spectra (300 MHz) of the reaction of compound **3** (30 mg, 0.065 mmol) with 1,5-hexadiene (23.3 µL, 0.20 mmol) in C_6D_6 after (a) 15 mins and (b) 24 hours. Assignment of major species: (BDI)Ca$(CH_2)_3$CH=CH_2: (BDI methine = 4.76, CH = 4.73 and α-CH_2 = −1.11 ppm) in 2:1:2 ratio. Compound **10**: (BDI methine = 4.78, Ca*H* = 4.84 and α-CH2 = –0.99 ppm) in 2:1:2 ratio. Compound **11**: (BDI methine = 4.72 and α-CH_2 = –0.54 ppm) in 1:2 ratio.

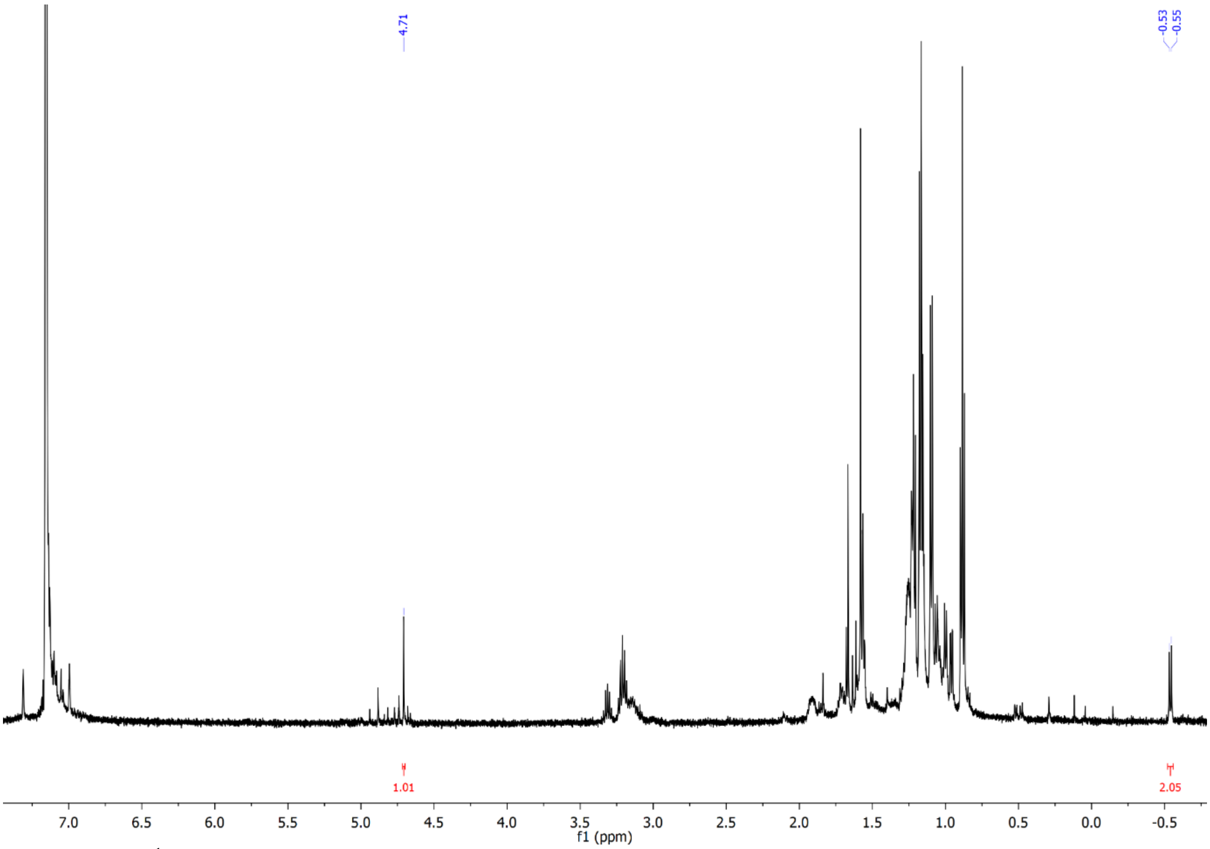

Figure S2: ^{1}H NMR spectrum (500 MHz) of material isolated from the reaction of compound **3** (30 mg, 0.065mmol) with 1,5-hexadiene (23.3 µL, 0.20 mmol) in C_6D_6 after 24 hours at room temperature.

Stoichiometric Hydrogenation

In a J Young NMR tube, C_6D_6 (0.5 mL) added to **9** (16 mg, 0.03 mmol), degassed via three freeze-pump-thaw cycles and hydrogen (2 bar) was added.

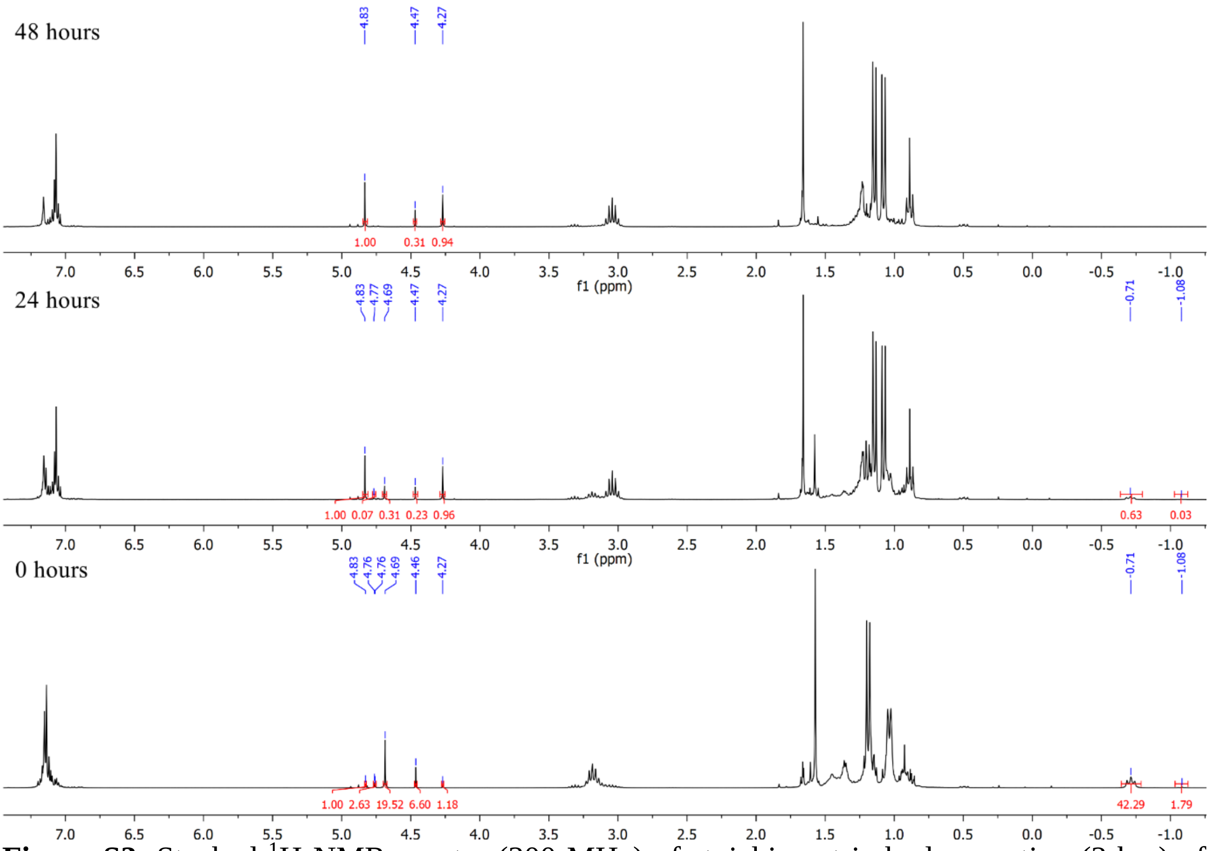

Figure S3: Stacked ^{1}H NMR spectra (300 MHz) of stoichiometric hydrogenation (2 bar) of compound **9** (0.03 mmol) in C6D6 (500 µL).

Assignment of major species: Compound **3** (BDI methine = 4.83, Ca*H* = 4.27 ppm) in 1:1 ratio. Compound **6** (BDI methine = 4.76, Ca*H* = 1.08 ppm) in a 3:2 ratio. Compound **9** (BDI methine = 4.69, α-CH_2 = –0.72 ppm) in a 1:2 ratio.

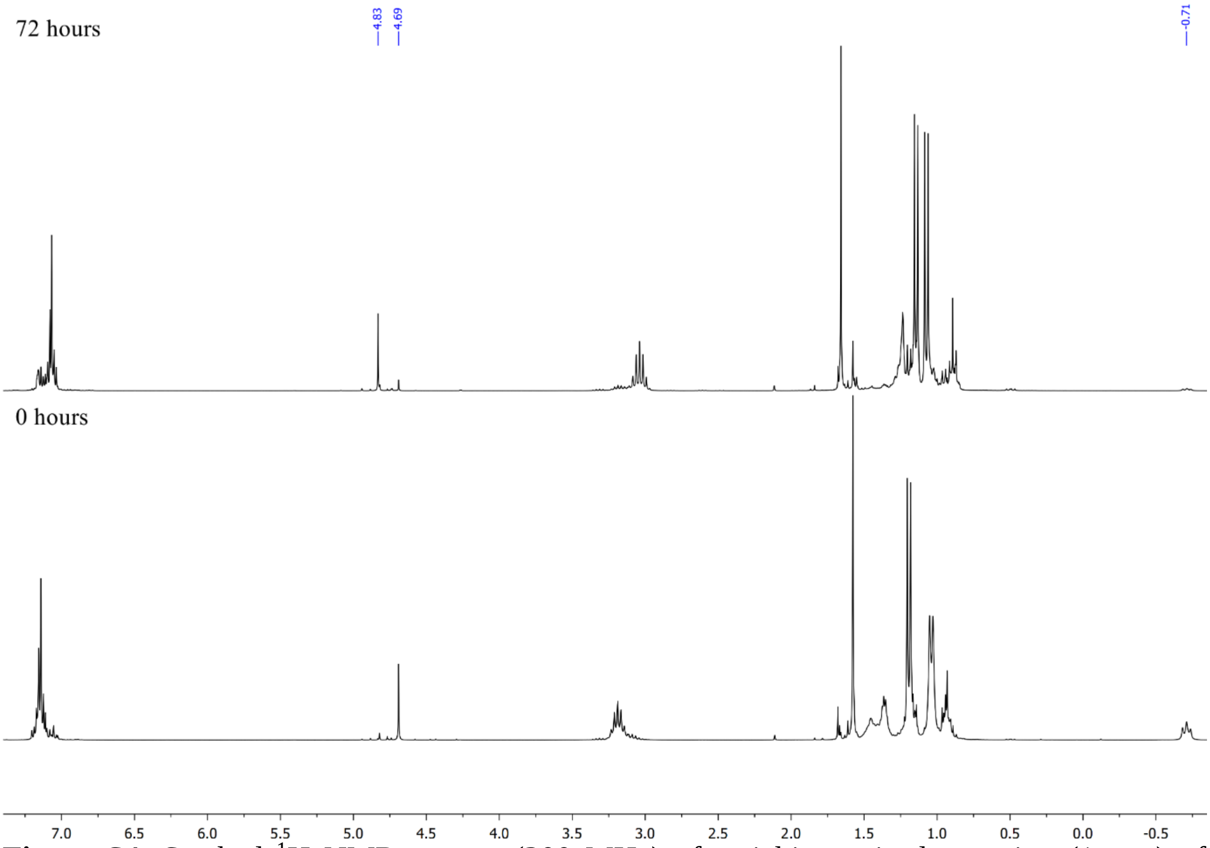

Figure S4: Stacked ^{1}H NMR spectra (300 MHz) of stoichiometric deuteration (1 atm) of compound **9** (0.072 mmol) in C_6D_6 (500 μL).

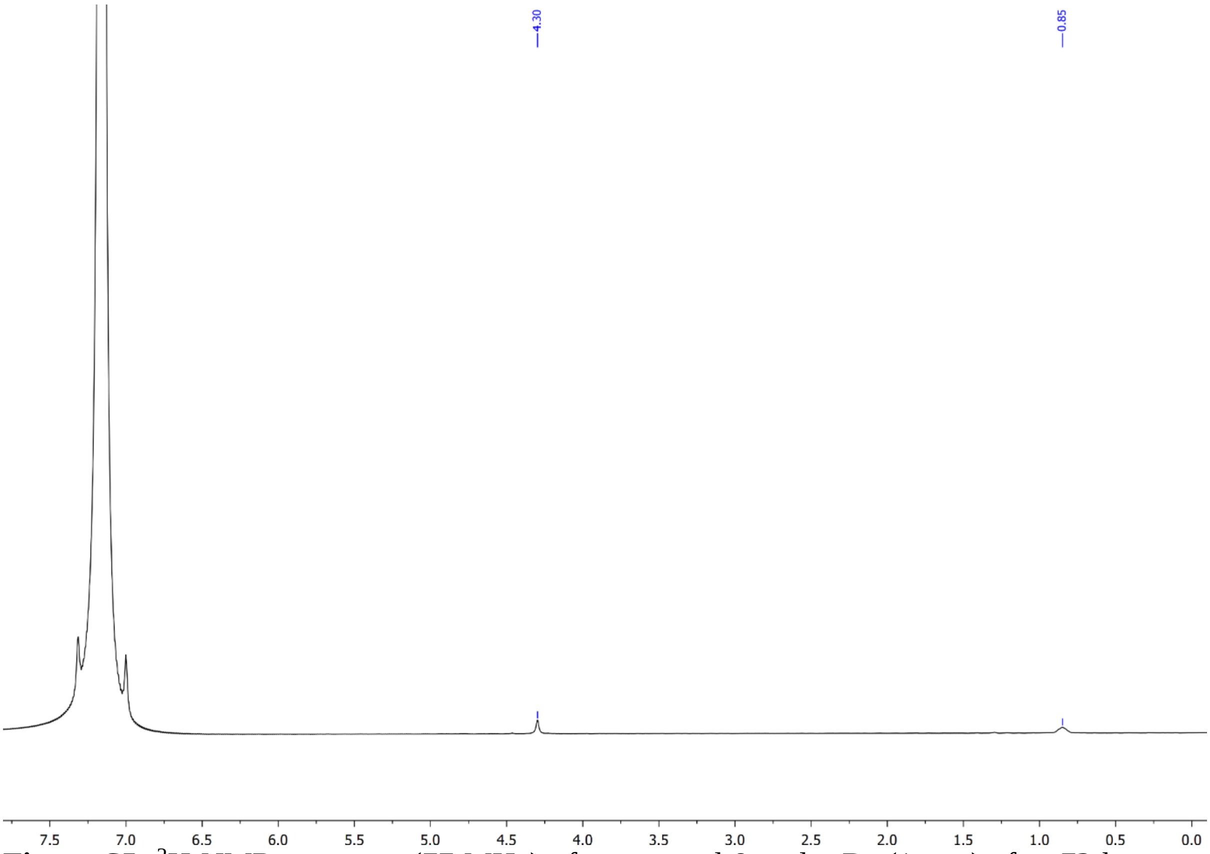

Figure S5: ^{2}H NMR spectrum (77 MHz) of compound **9** under D_2 (1 atm) after 72 hours at room temperature (C_6D_6 solvent).

General Method for Catalytic Hydrogenation

In a J Young NMR tube, the catalyst, **3** (10 mol%), and the olefinic substrate (0.11 mmol) were dissolved in C_6D_6 (500 µL). The solutions were degassed via three freeze-pump-thaw cycles and charged with hydrogen (2 bar). The reactions were monitored via ^{1}H NMR spectroscopy with their progress evidenced by the consumption of the olefinic resonances alongside the emergence of the respective hydrocarbon resonances. An aliquot of a stock C_6D_6 solution of hexamethylbenzene (55 µL, 0.5 mmolmL^{-1}) was added as an internal standard after the disappearance of the olefinic resonances of the reactants.

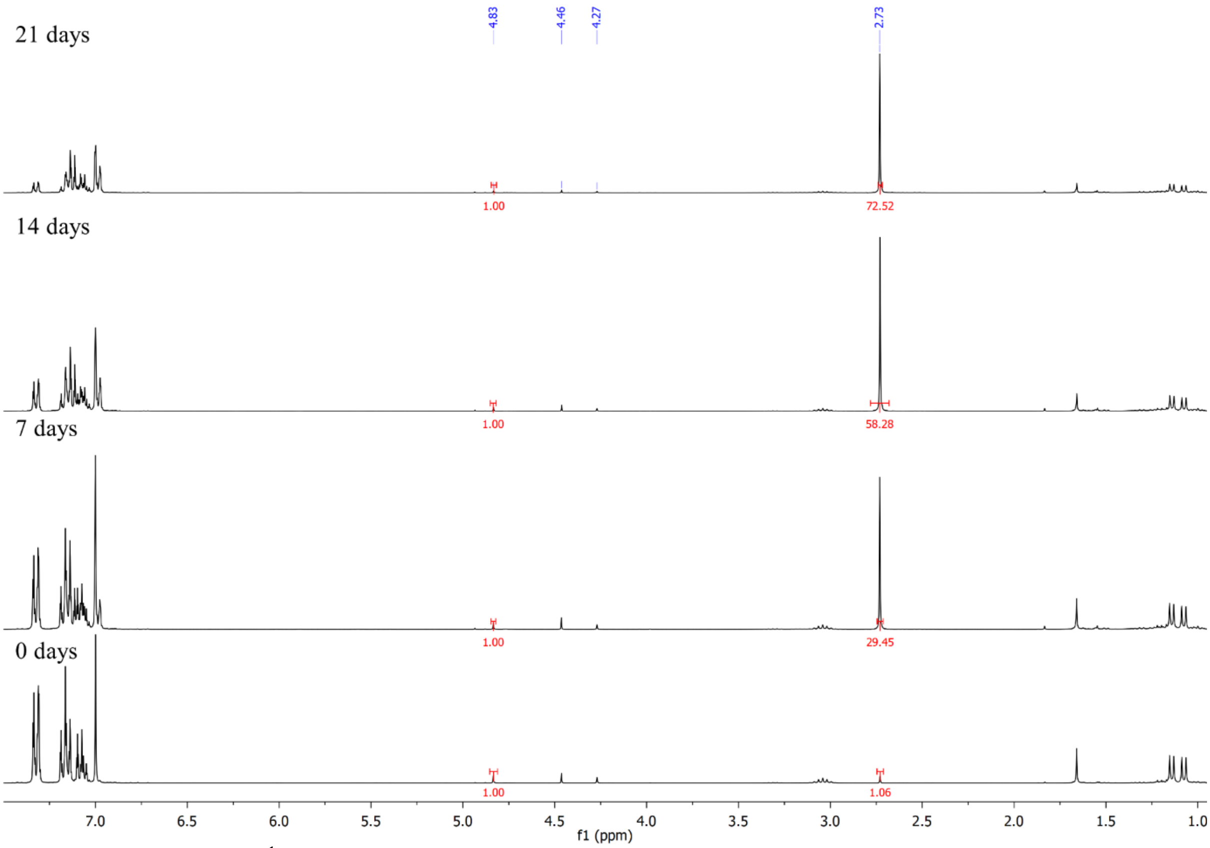

Figure S6: Stacked ^{1}H NMR spectra (300 MHz) of catalytic hydrogenation (2 bar) of *trans*-stilbene (0.11 mmol) mediated by compound **3** (0.01 mmol) in C_6D_6 (500 μL).

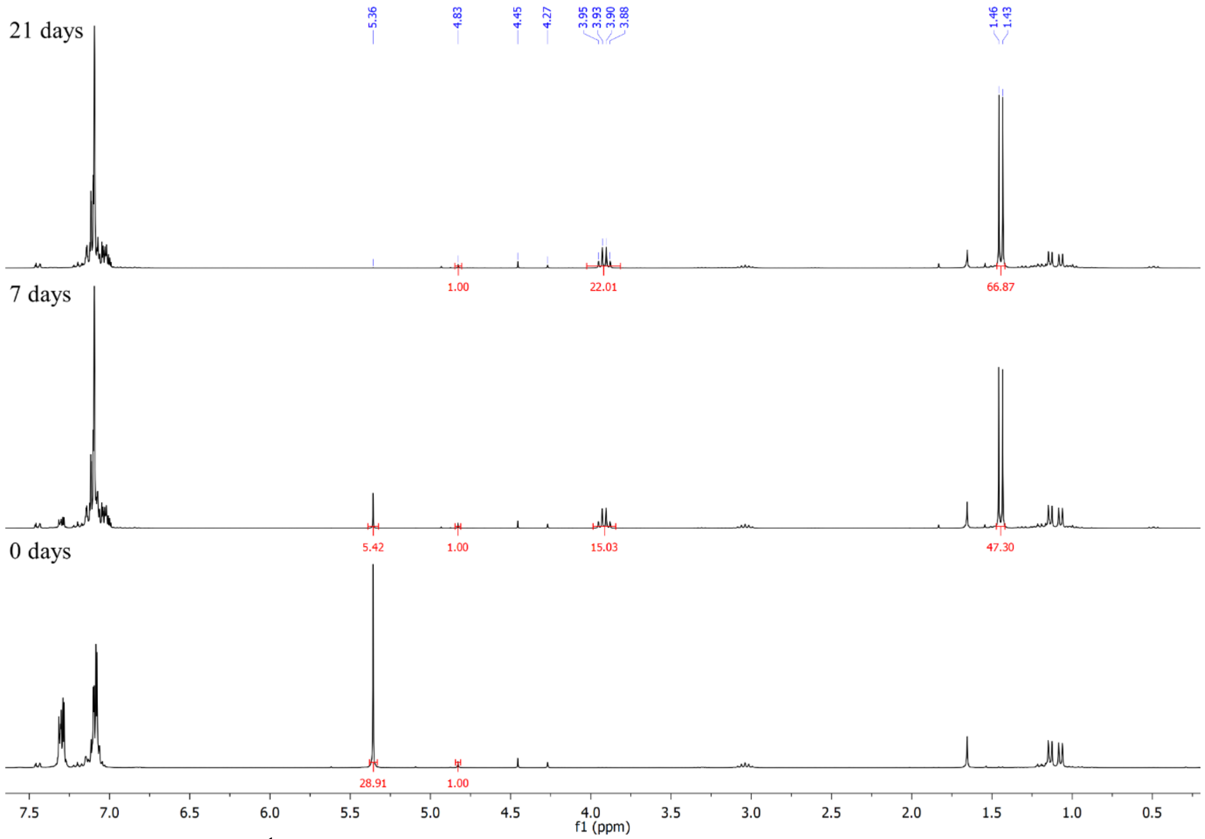

Figure S7: Stacked ^{1}H NMR spectra (300 MHz) of catalytic hydrogenation (2 bar) of 1,1-diphenylethene (0.11 mmol) mediated by compound 3 (0.01 mmol) in C_6D_6 (500 μL).

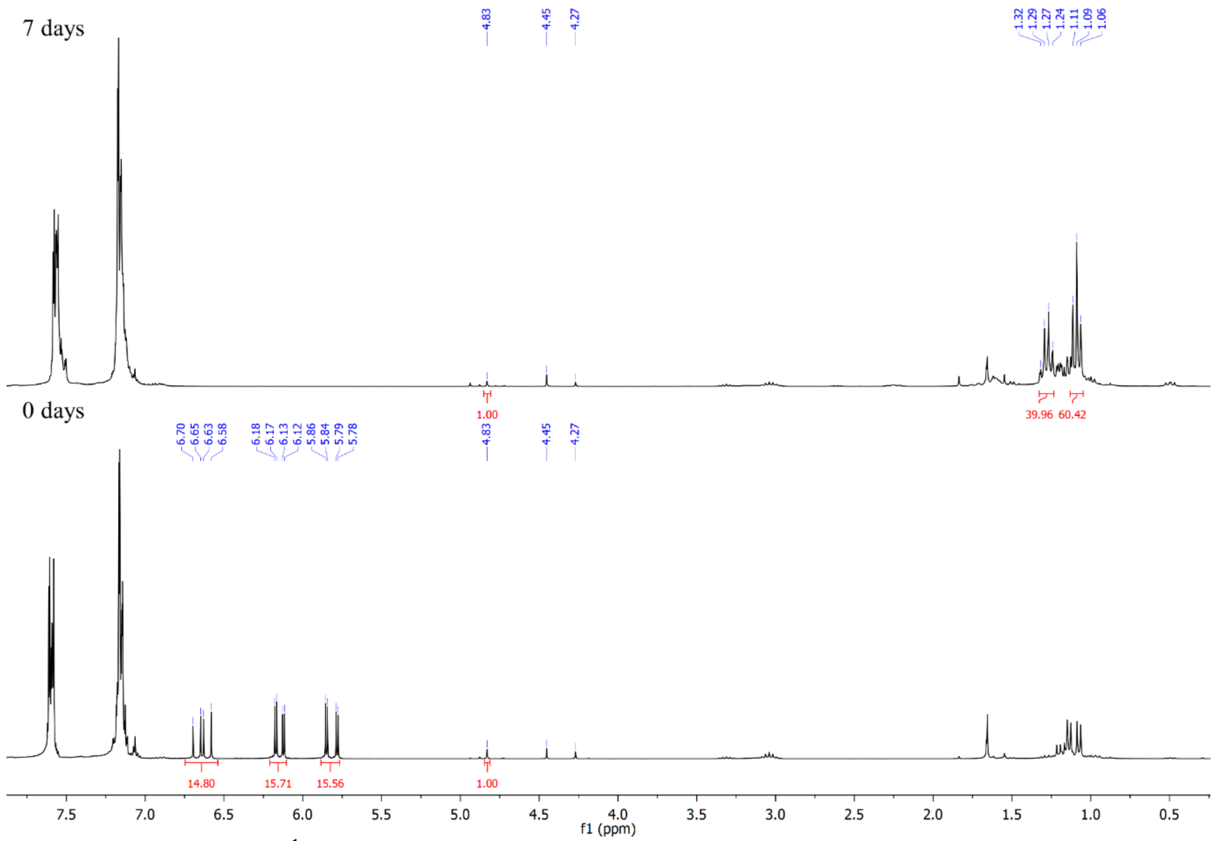

Figure S8: Stacked ^{1}H NMR spectra (300 MHz) of catalytic hydrogenation (2 bar) of triphenyl(vinyl)silane (0.11 mmol) mediated by compound **3** (0.01 mmol) in C_6D_6 (500 μL).

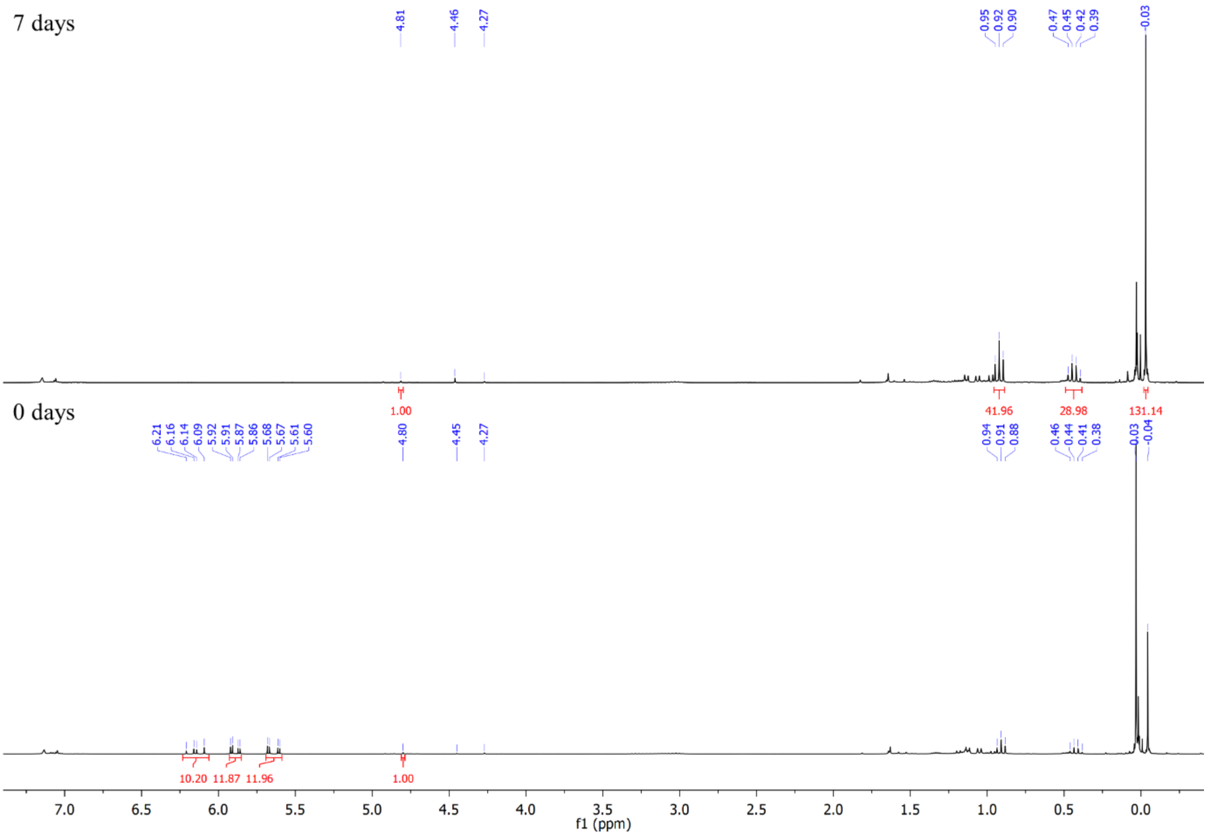

Figure S9: Stacked ^{1}H NMR spectra (300 MHz) of catalytic hydrogenation (2 bar) of vinyltrimethylsilane (0.11 mmol) mediated by compound **3** (0.01 mmol) in C_6D_6 (500 μL).

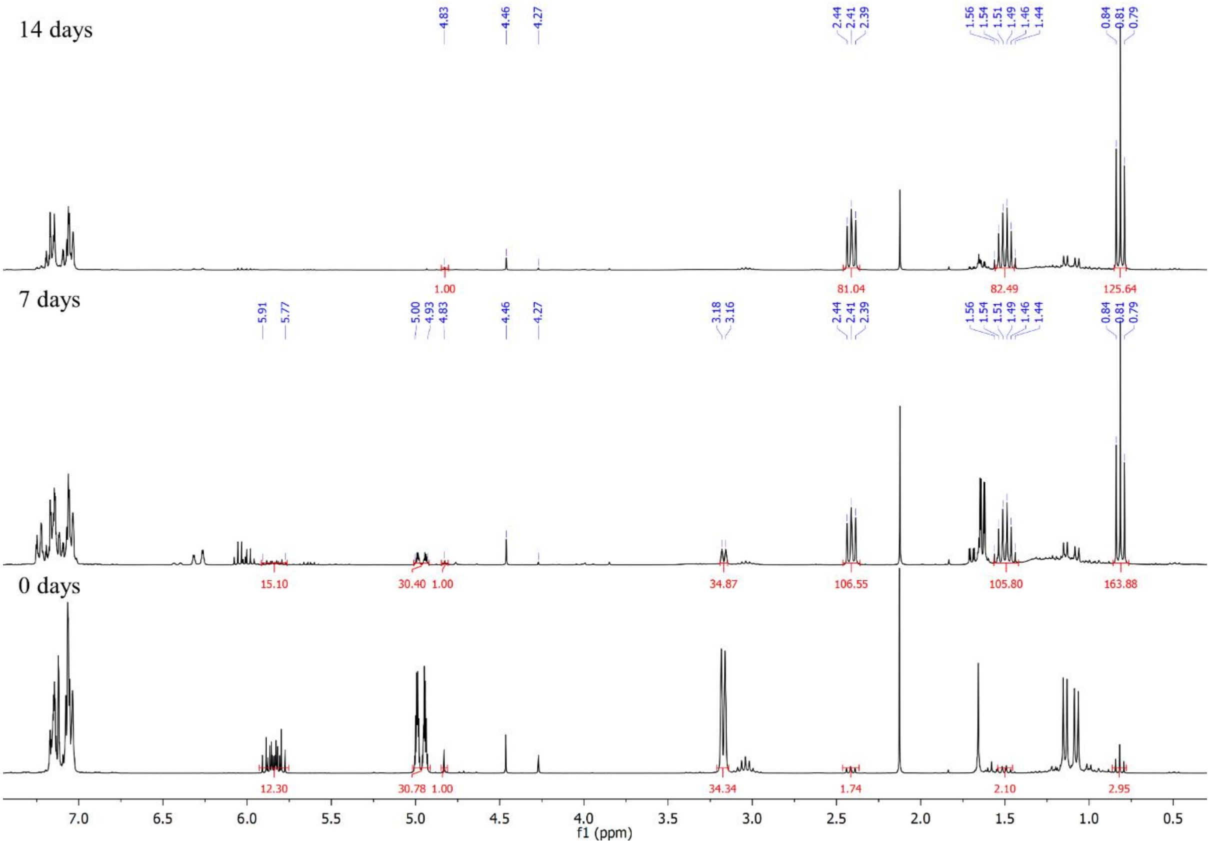

Figure S10: Stacked ^{1}H NMR spectra (300 MHz) of catalytic hydrogenation (2 bar) of allylbenzene (0.11 mmol) mediated by compound **3** (0.01 mmol) in C_6D_6 (500 μL).

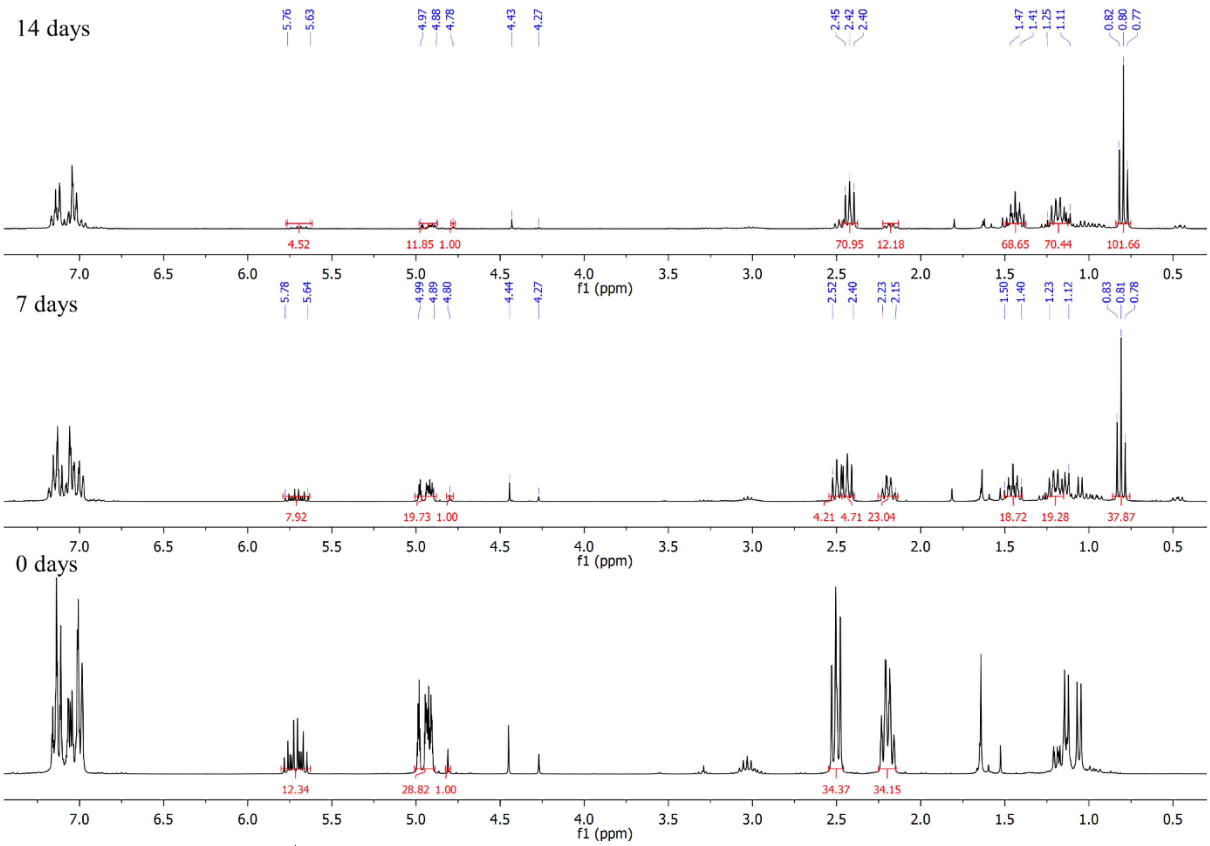

Figure S11: Stacked ^{1}H NMR spectra (300 MHz) of catalytic hydrogenation (2 bar) of 4-phenyl-1-butene (0.11 mmol) mediated by compound **3** (0.01 mmol) in C_6D_6 (500 μL).

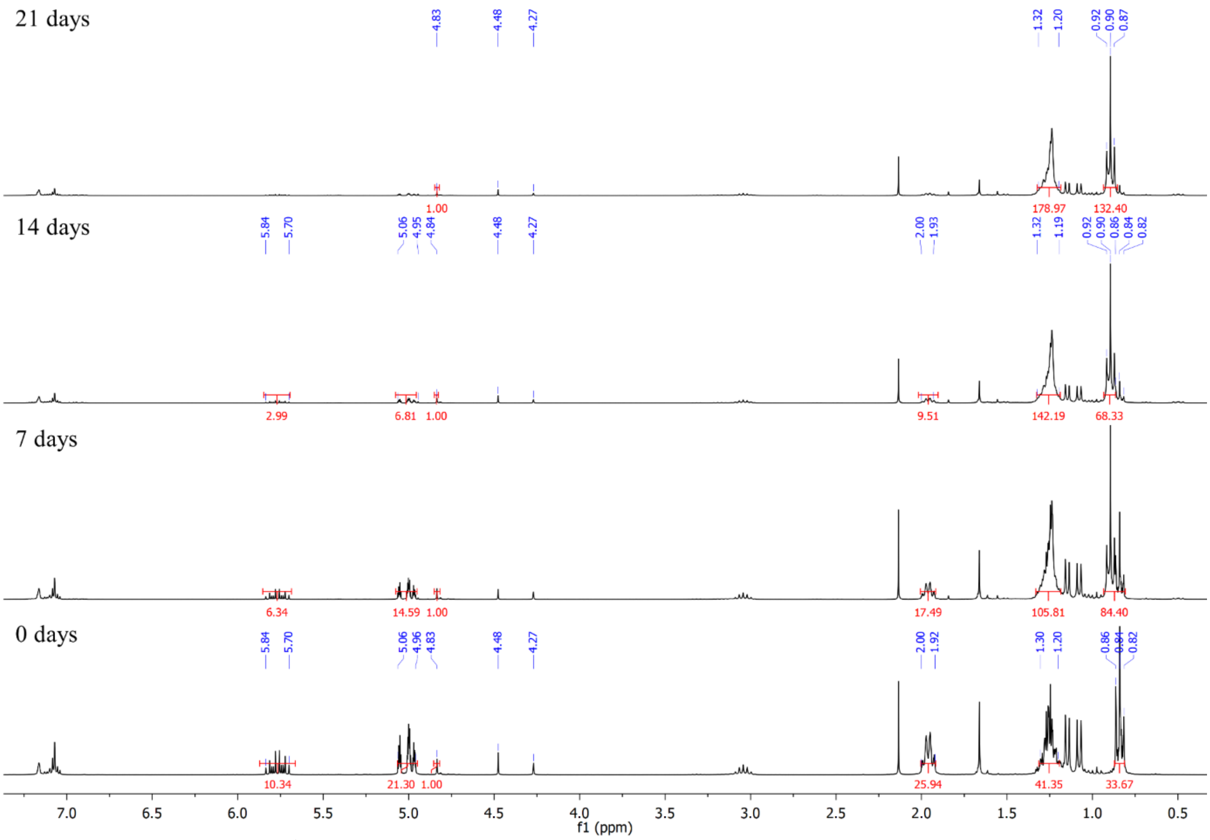

Figure S12: Stacked ^{1}H NMR spectra (300 MHz) of catalytic hydrogenation (2 bar) of 1-hexene (0.11 mmol) mediated by compound **3** (0.01 mmol) in C_6D_6 (500 μL).

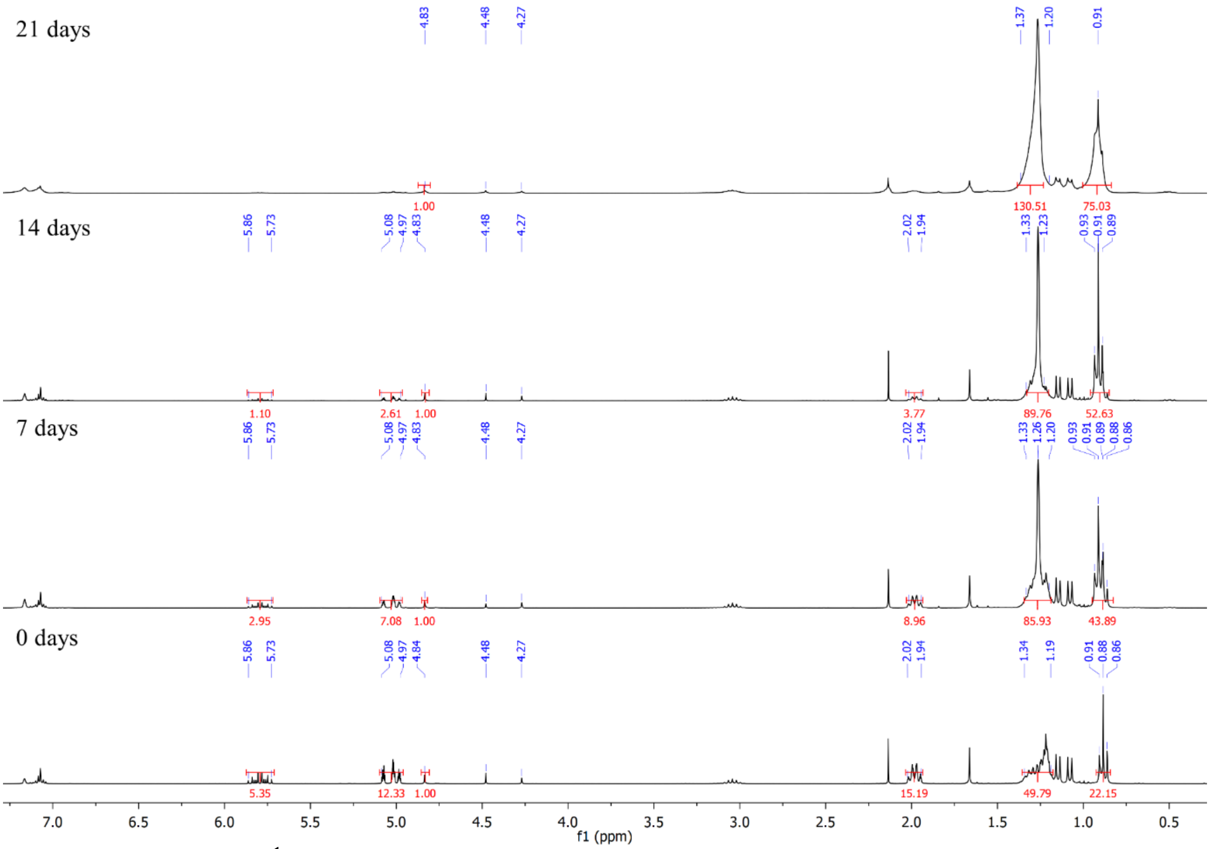

Figure S13: Stacked ^{1}H NMR spectra (300 MHz) of catalytic hydrogenation (2 bar) of 1-octene (0.11 mmol) mediated by compound **3** (0.01 mmol) in C_6D_6 (500 μL).

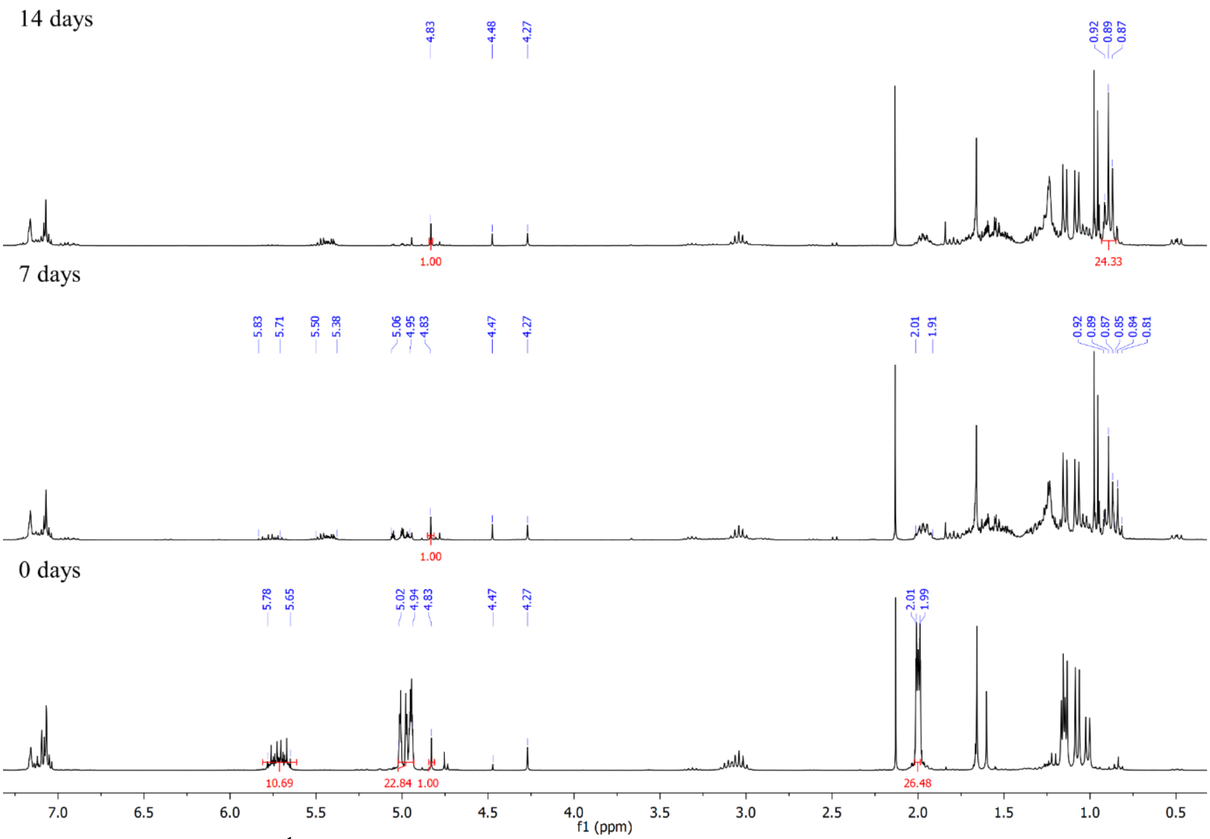

Figure S14: Stacked ^{1}H NMR spectra (300 MHz) of catalytic hydrogenation (2 bar) of 1,5-hexadiene (0.11 mmol) mediated by compound **3** (0.02 mmol) in C_6D_6 (500 μL).

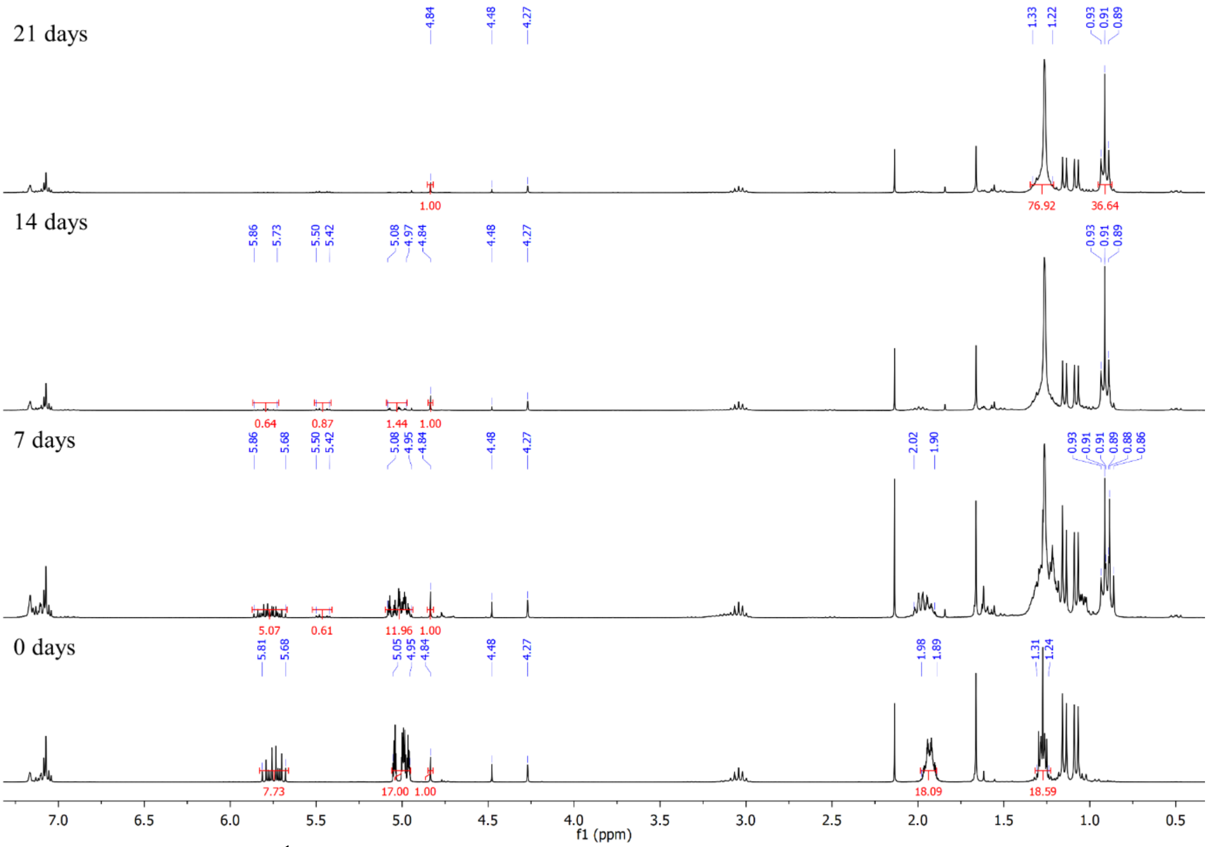

Figure S15: Stacked ^{1}H NMR spectra (300 MHz) of catalytic hydrogenation (2 bar) of 1,7-octadiene (0.11 mmol) mediated by compound **3** (0.02 mmol) in C_6D_6 (500 μL).

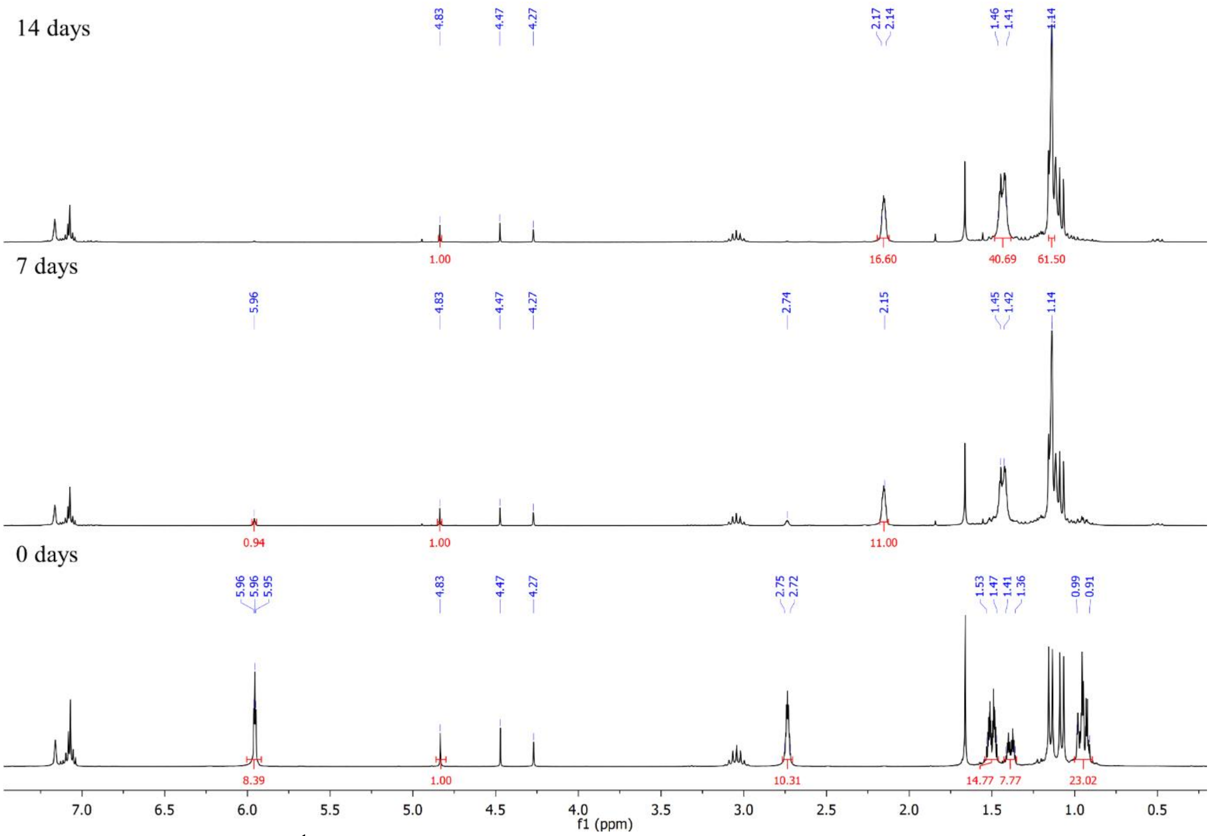

Figure S16: Stacked ^1H NMR spectra (300 MHz) of catalytic hydrogenation (2 bar) of norbornene (0.11 mmol) mediated by compound **3** (0.01 mmol) in C_6D_6 (500 μL).

Cross-over experiments

In a J Youngs NMR tube, C_6D_6 (0.5 mL) was added to compound **9** (39 mg, 0.07 mmol), the solution was subjected to three freeze-pump-thaw degassing cycles and backfilled with an atmosphere of 1-butene.

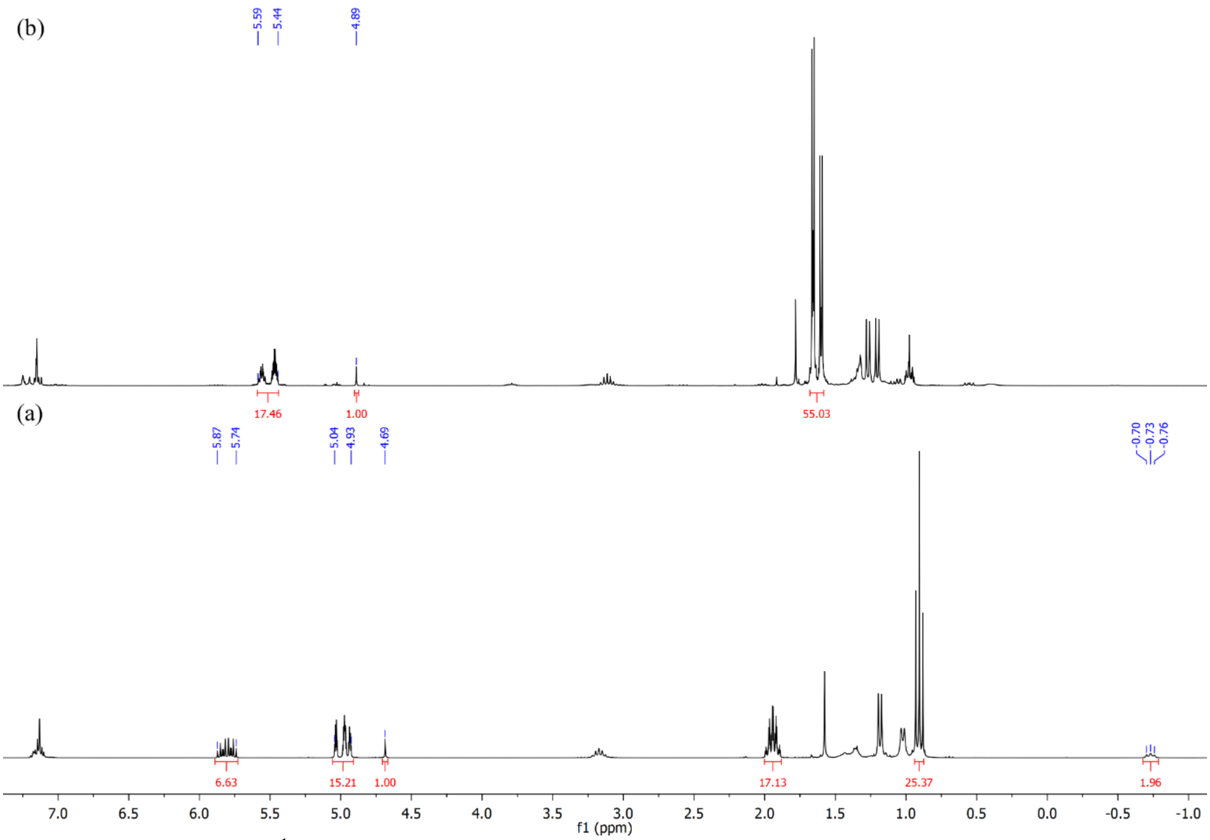

Figure S17: Stacked ^{1}H NMR spectra (300 MHz) of the reaction of compound **9** (39 mg, 0.07 mmol) with 1-butene (1 atm) in C_6D_6 after (a) 15 mins and (b) 28 days.

In a J Youngs NMR tube, C_6D_6 (0.5 mL) was added to a mixture of compound **9** (10 mg, 0.02 mmol) and compound **3** (8 mg, 0.02 mmol).

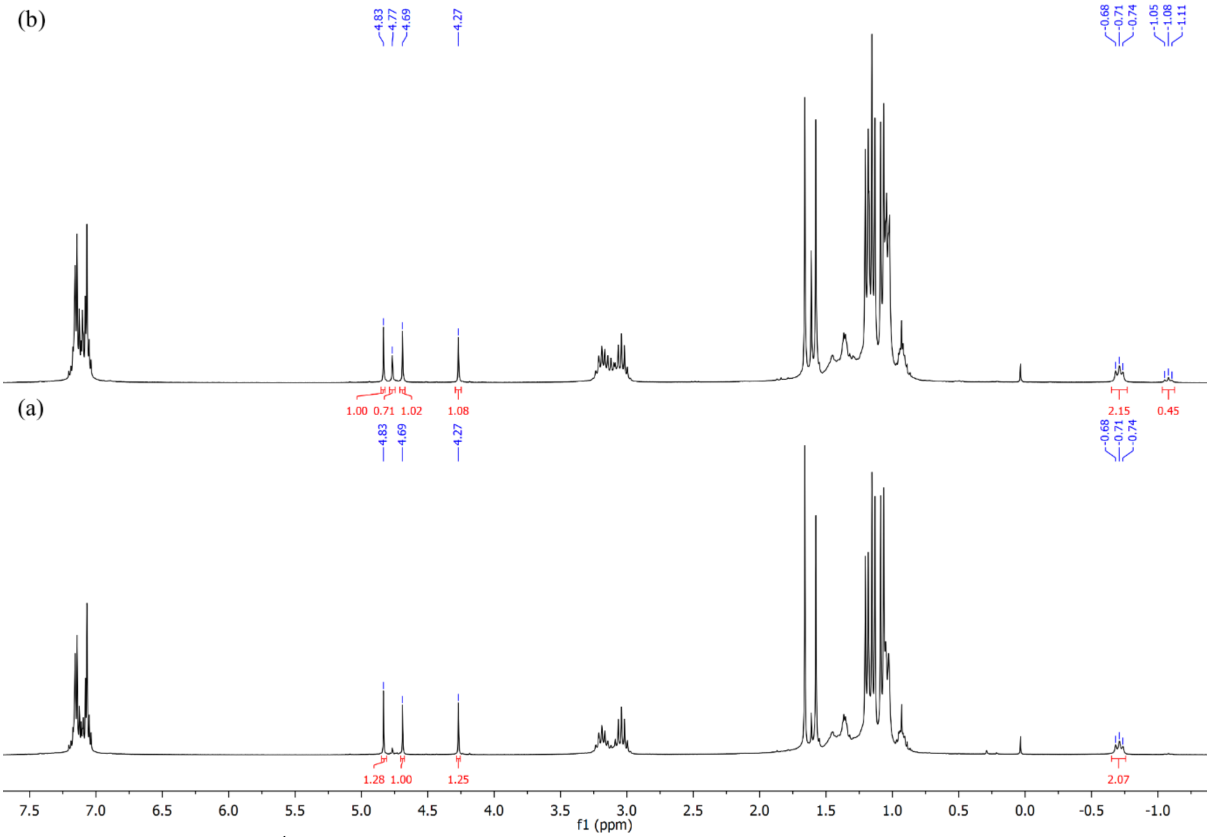

Figure S18: Stacked ^{1}H NMR spectra (300 MHz) of the reaction of compound **9** (10 mg, 0.02 mmol) with compound **3** (8 mg, 0.02 mmol) in C_6D_6 after (a) 15 mins and (b) 1 day. Assignment of major signals: Compound **3**: 4.83 (BDI, 1H), 4.27 (H, 1H); Compound **9** 4.69 (BDI, 1H), −0.71 (α-CH_2, 2H); Compound **6**: 4.77 (BDI methine + Ca*H*, 3H), −1.08 (α-CH_2, 2H).

In a J Youngs NMR tube, C_6D_6 (0.5 mL) was added to a mixture of compound **3** (30 mg, 0.065 mmol) and compound **3**-d_2 (30 mg, 0.065 mmol).

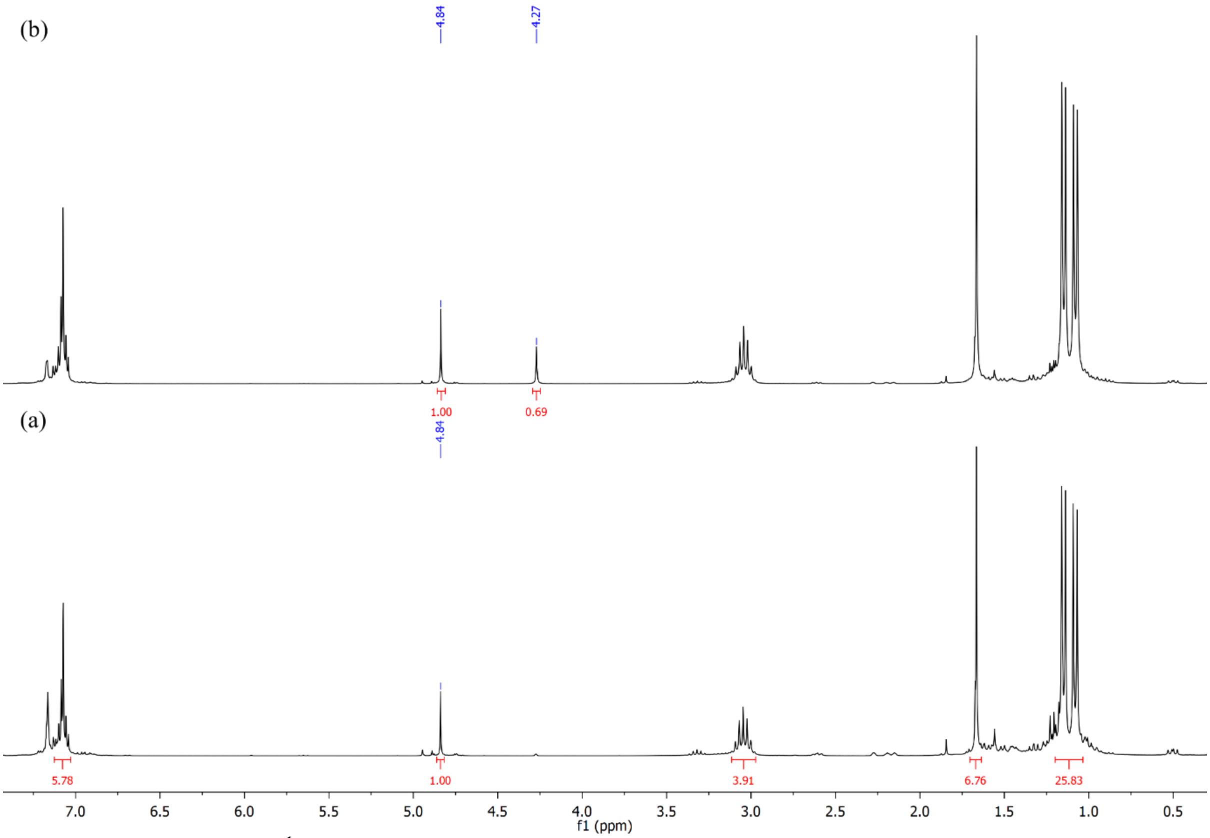

Figure S19: Stacked ^{1}H NMR spectra (300 MHz) of (a) compound **3**-d_2 and (b) the reaction of **3**-d_2 (30 mg, 0.065 mmol) and **3** (30 mg, 0.065 mmol) in C_6D_6.

X-ray Crystallography.

Single Crystal X–ray diffraction data for compound **11** were collected on a SuperNova, EosS2 diffractometer using Cu radiation throughout. The crystals were maintained at 150 K during data collection. Using Olex2,[2] the structures were solved with the olex2.solve[3] structure solution program and refined with the ShelXL[4] refinement package using Least Squares minimization. The asymmetric unit of **11** comprises half of a dimer molecule. The remainder is generated by virtue of a crystallographic inversion centre proximate to Ca1. The hydrogen atoms attached to C30 were located and refined subject to having C-H distances of 0.98 Å. There is no evidence for a locus of electron density in the solvent accessible voids (101 Å^3) of this gross structure.

Table S1: Single Crystal X–ray Data Parameters for compound **11**.

Empirical formula	$C_{35}H_{52}CaN_2$
Formula weight	540.86
Temperature/K	149.99(10)
Crystal system	monoclinic
Space group	*C*2/*c*
a/Å	21.9197(2)
b/Å	14.3822(1)
c/Å	23.0389(2)
α/°	90
β/°	112.7940(10)
γ/°	90
Volume/Å^3	6695.87(11)
Z	8
ρ_{calc}g/cm^3	1.073
μ/mm^{-1}	1.771
F(000)	2368.0
Crystal size/mm^3	0.346 × 0.256 × 0.132
2Θ range for data collection/°	7.544 to 146.246
Index ranges	$-27 \le h \le 27$, $-16 \le k \le 17$, $-26 \le l \le 28$
Reflections collected	41731
Independent reflections	6701 [R_{int} = 0.0206, R_{sigma} = 0.0132]
Data/restraints/parameters	6701/2/369
Goodness-of-fit on F^2	1.044
Final *R* indexes [I>=2σ (I)]	R_1 = 0.0456, wR_2 = 0.1344
Final *R* indexes [all data]	R_1 = 0.0467, wR_2 = 0.1356
Largest diff. peak/hole / e Å^{-3}	0.48/-0.34
Flack parameter	–

.

Computational details

Calculations were carried out using the Gaussian09 package[5] at the DFT level by means of the hybrid density functional B3PW91.[6] A triple-zeta 6-311G basis set augmented by a polarization and diffuse function was used for the Ca atom. Polarized all electron triple-zeta 6-311G(d,p)[7] basis set were used for N, whereas a polarized all electron double-zeta 6-31G(d,p)[8] basis set were used for the C and H atoms. The nature of the optimized stationary point, minima or transition state, has been verified by means of analytical frequency calculation at 298.15 K and 1 atm. The geometry optimizations have been achieved without any geometrical constraints. IRC calculations were carried out in order to confirm the connectivity between reactant(s), transition state and product(s). Energy data are reported in the gas phase. Natural Bonding Analysis (NBO) were carried out to get the Natural Population Analysis (NPA).

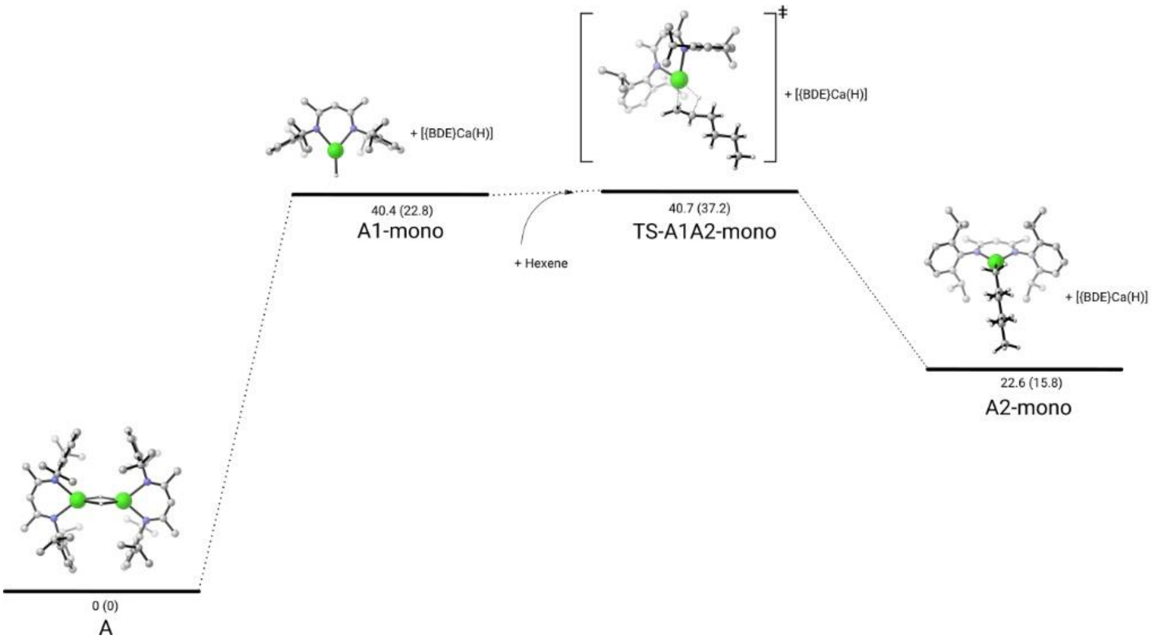

Figure S20: DFT (B3PW91) computed enthalpy reaction profile at room temperature for hexane insertion via initial hydride dimer dissociation. Computed to be endothermic by 40.4 kcal mol^{-1}.

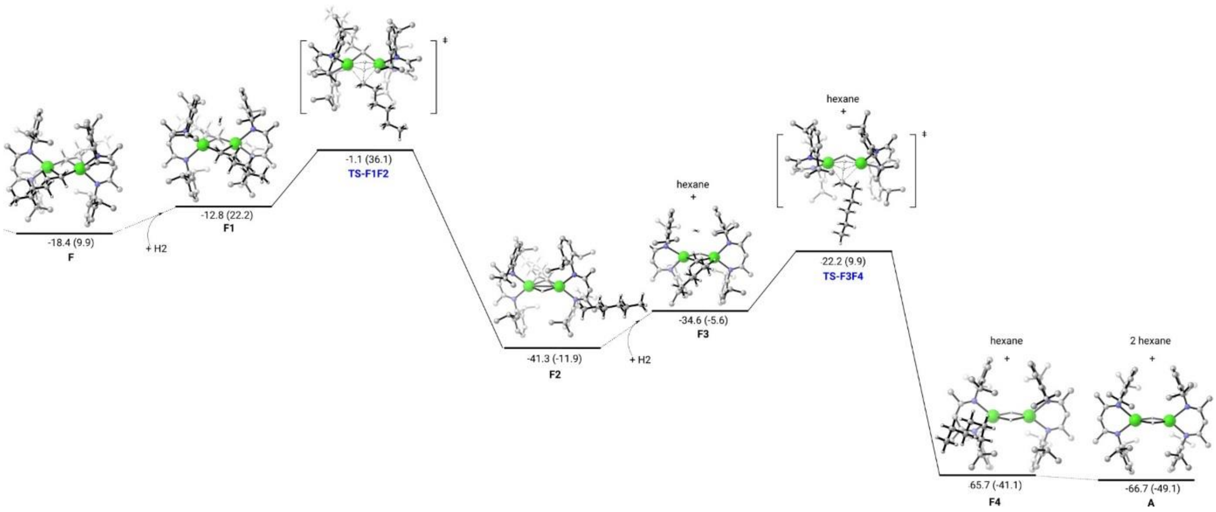

Figure S21: DFT (B3PW91) computed enthalpy reaction profile at room temperature for the direct hydrogenation via 1-hexene insertion of the dicalcium n-hexyl-hydride complex **C** (**6**).

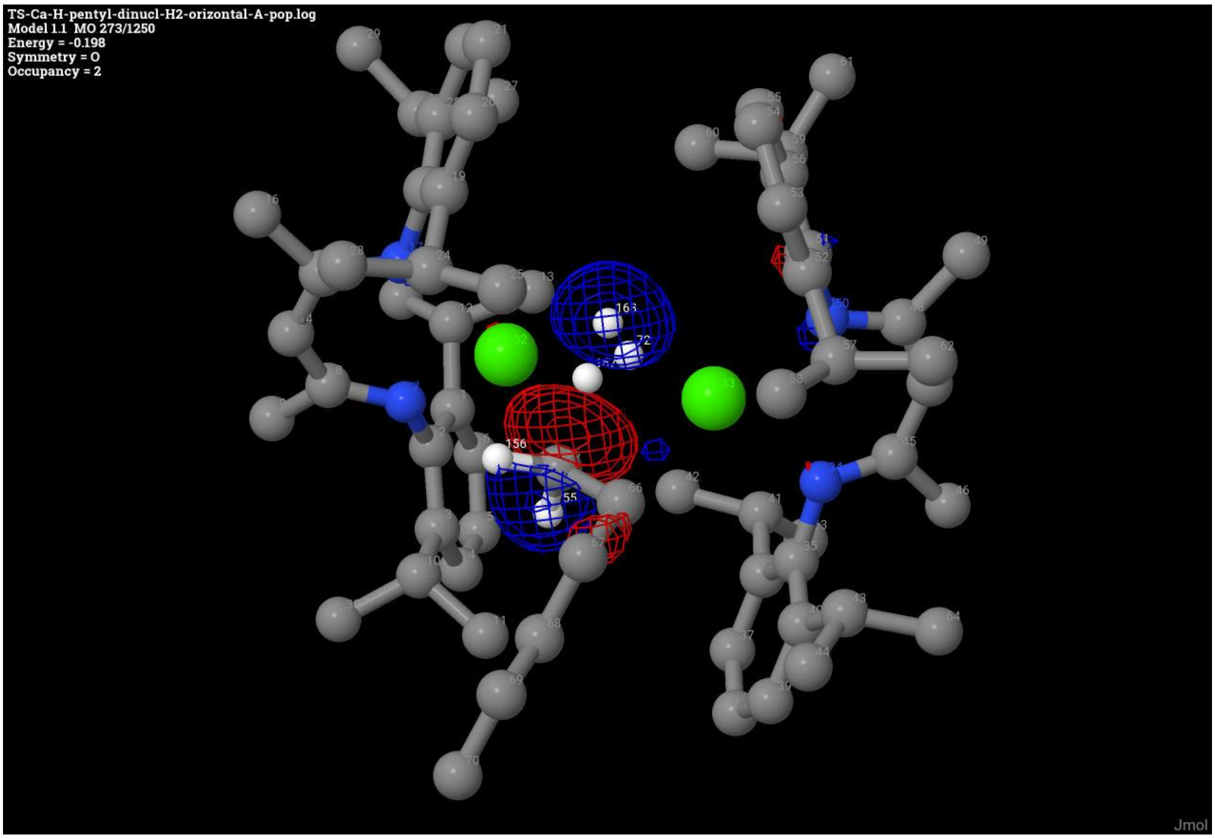

Figure S22: 3D plot of the HOMO-2 orbital at the TS for H_2 activation by the *n*-hexyl-hydride complex, **C** (**6**).

The HOMO-2 orbital indicates a heterolytic rupture of the H167-H168 bond. H168 can be represented schematically by an H^- and H167 by an H^+. The H168 would interact with the two Ca atoms while the H167 would interact with the C65. The NBO displays a strong second order interaction between the sp^3 filled orbital of the C65 and the σ* orbital of the H-H molecule, indicating that the H-H bond is broken by reaction with the hexyl group. By a less strong second order interaction between the σ^*_{H-H} orbital and the two Ca atoms (Ca32 and Ca33) on one side and the H168 atom on the other side, the σ^*_{H-H} orbital redistributes this density to both Ca centers and to the H168 atom. The charge analysis indicates a charge of -1 for C65, a charge of 1.50 and 1.43 for Ca32 and Ca33 respectively, a charge of 0.03 and -0.23 for H167 and H168 and a charge of -0.65 for the hydride ligand H72.

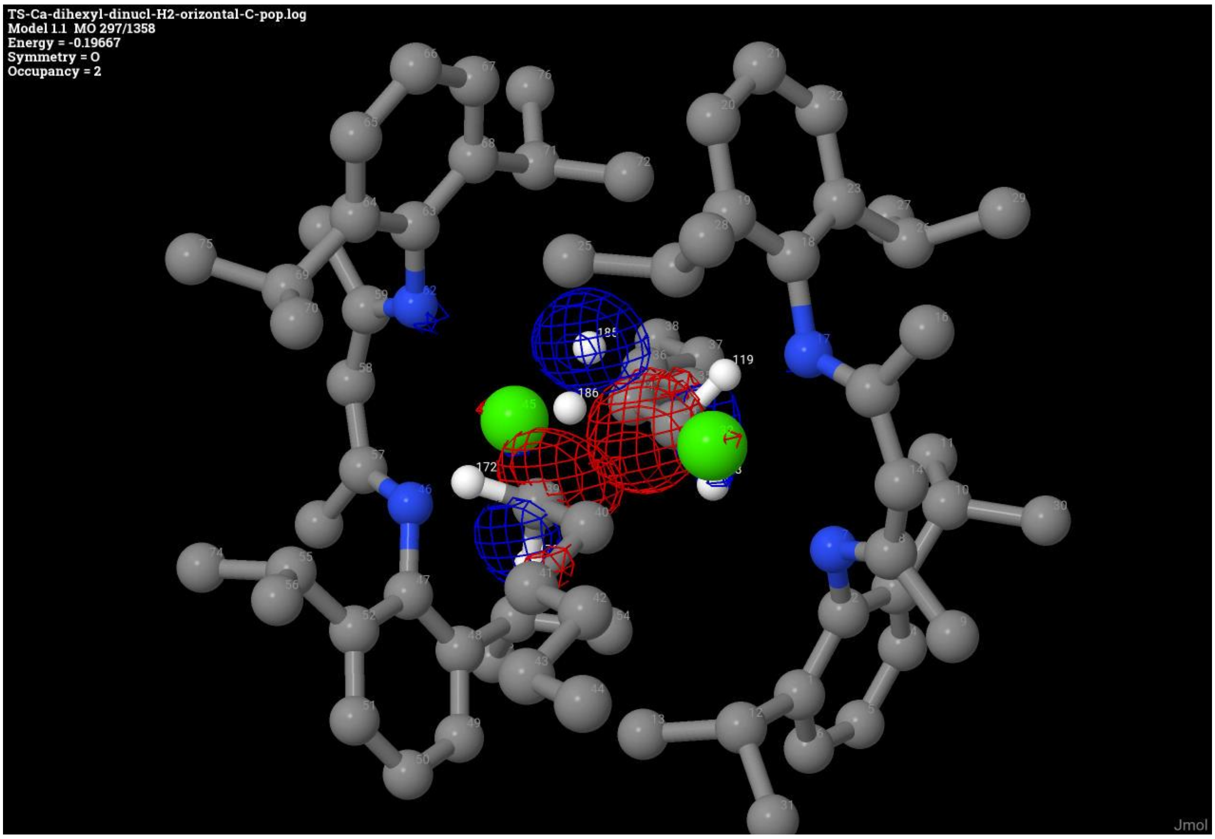

Figure S23: 3D plot of the HOMO-2 orbital at the TS for H_2 activation by the dimeric dicalcium di-*n*-hexyl complex, **F** (**9**).

The HOMO-2 orbital indicates a heterolytic rupture of the H185-H186 bond. H185 can be represented schematically by an H^- and H186 by an H^+. The H185 would interact with the two Ca atoms while the H186 would interact with the C39. The NBO displays a strong second order interaction between the sp^3 filled orbital of the C39 and the σ* orbital of the H-H molecule, indicating that the H-H bond is broken by reaction with the hexyl group. By a less strong second

order interaction between the $\sigma^*_{H\text{-}H}$ orbital and the two Ca atoms (Ca45 and Ca32) on one side and the H185 atom on the other side, the $\sigma^*_{H\text{-}H}$ orbital redistributes this density to both Ca centers and to the H185 atom. The charge analysis indicates a charge of -1.16 and -1 for the C33 and C39 respectively, a charge of 1.50 and 1.51 for the Ca32 and Ca45 respectively and a charge of -0.23 and 0.03 for the H185 and H186 respectively.

Cartesian coordinates of all optimized structures

148

[**A**]

Ca	22.087289	19.587777	7.936914
H	21.114637	19.853472	5.957691
N	22.741374	21.277936	9.451527
N	21.089736	18.780910	9.922578
C	22.835629	22.721020	11.438986
H	22.530411	23.635186	10.918305
H	22.448446	22.758489	12.459702
H	23.929965	22.739309	11.481324
C	22.344619	21.490787	10.702119
C	21.492154	20.639001	11.432092
H	21.262259	20.980114	12.435252
C	20.955020	19.379067	11.102005
C	20.198994	18.674059	12.210181
H	20.717189	17.753375	12.502040
H	20.099881	19.307610	13.094549
H	19.199356	18.375762	11.878231
C	23.680555	22.161609	8.845334
C	25.064058	21.853818	8.899811
C	25.595029	20.676205	9.706912
H	24.740109	20.205191	10.204377
C	26.573442	21.127069	10.802100
H	26.121092	21.868642	11.469527
H	26.885358	20.270086	11.411467
H	27.478166	21.575023	10.373704
C	26.252537	19.617542	8.809472
H	27.125058	20.026972	8.286125
H	26.594991	18.765106	9.409137
H	25.560327	19.238858	8.048541
C	25.963051	22.666734	8.202966
H	27.025982	22.438258	8.242428
C	25.526951	23.760304	7.463989
H	26.241109	24.378341	6.925541
C	24.170415	24.061340	7.423653
H	23.832590	24.920004	6.848450
C	23.230324	23.286479	8.109057
C	21.759168	23.675880	8.054525
H	21.220841	23.037493	8.764228
C	21.529878	25.134026	8.481299
H	20.455872	25.348872	8.535991
H	21.968153	25.345729	9.463008
H	21.968839	25.838709	7.764955
C	21.158972	23.431191	6.662433
H	21.219967	22.376901	6.367554
H	20.100852	23.721383	6.642865
H	21.684453	24.015654	5.897797
C	20.575668	17.465741	9.731196
C	19.260181	17.280325	9.235790
C	18.303627	18.449024	9.041073
H	18.780113	19.340908	9.463202
C	16.975810	18.241963	9.785986
H	16.403808	17.405110	9.367780
H	17.132677	18.035831	10.850714

H	16.351542	19.139826	9.703373	C	24.261224	19.799143	0.243433
C	18.039064	18.723679	7.554061	H	23.714163	20.703983	-0.045114
H	17.354469	19.572858	7.434190	H	24.382709	19.173330	-0.643670
H	18.960937	18.960336	7.009823	H	25.250203	20.128174	0.578562
H	17.584834	17.852604	7.067004	C	20.899327	16.168063	3.580523
C	18.833089	15.984699	8.931032	C	19.506834	16.432746	3.531358
H	17.828783	15.834574	8.541977	C	18.937568	17.596837	2.730752
C	19.662819	14.884917	9.116736	H	19.776386	18.096213	2.233386
H	19.310929	13.885985	8.871569	C	17.971568	17.119830	1.635688
C	20.942735	15.071958	9.626004	H	18.445834	16.394936	0.965188
H	21.586043	14.208925	9.783813	H	17.632263	17.968920	1.029930
C	21.420480	16.346635	9.944065	H	17.081912	16.642319	2.063760
C	22.820676	16.498074	10.522675	C	18.249549	18.630700	3.633812
H	22.958527	17.554108	10.779563	H	17.392894	18.191624	4.159247
C	23.000166	15.684307	11.813493	H	17.877604	19.473773	3.038623
H	22.933845	14.606285	11.623383	H	18.931894	19.029141	4.393521
H	23.985035	15.881057	12.254272	C	18.635314	15.587739	4.224850
H	22.239015	15.935689	12.560345	H	17.565887	15.784681	4.191588
C	23.904810	16.121092	9.502657	C	19.106816	14.501497	4.952684
H	23.833654	16.719869	8.587123	H	18.413704	13.858430	5.489240
H	24.904086	16.273153	9.928939	C	20.471902	14.240346	4.984551
H	23.824122	15.067009	9.210881	H	20.837461	13.386087	5.549573
Ca	22.402006	18.791934	4.507375	C	21.385919	15.050230	4.304414
H	23.370681	18.517261	6.487735	C	22.868381	14.705629	4.352228
N	21.808735	17.088001	2.982890	H	23.385640	15.367838	3.648852
N	23.363713	19.654791	2.526724	C	23.141833	13.259807	3.909980
C	21.768234	15.659193	0.983649	H	24.221861	13.078876	3.851190
H	22.086326	14.750067	1.505055	H	22.708216	13.044670	2.926975
H	22.171210	15.635607	-0.031362	H	22.726327	12.534655	4.619833
H	20.675250	15.616136	0.924297	C	23.462660	14.954235	5.746038
C	22.217206	16.898223	1.732415	H	23.362630	16.001520	6.054481
C	23.042608	17.784082	1.011746	H	24.530722	14.702756	5.760370
H	23.289961	17.455726	0.008488	H	22.960806	14.339681	6.503018
C	23.527650	19.063718	1.347145	C	23.816275	20.992855	2.716015

C	25.122534	21.240556	3.208682
C	26.130877	20.117526	3.409767
H	25.702773	19.206457	2.976781
C	27.457111	20.391289	2.684306
H	27.986748	21.247497	3.118531
H	27.303956	20.602376	1.619988
H	28.119533	19.521023	2.765197
C	26.386481	19.847717	4.899258
H	27.117790	19.039437	5.025798
H	25.471306	19.552205	5.425859
H	26.777444	20.740775	5.400984
C	25.490785	22.555997	3.505565
H	26.486972	22.753832	3.893966
C	24.612449	23.616111	3.312824
H	24.919869	24.631775	3.549220
C	23.341585	23.368152	2.807104
H	22.660641	24.200601	2.643359
C	22.920827	22.070970	2.499549
C	21.526404	21.854498	1.927712
H	21.430543	20.790383	1.685476
C	21.309933	22.642441	0.626518
H	21.333665	23.724809	0.801505
H	20.332086	22.400160	0.192853
H	22.077582	22.411214	-0.120168
C	20.432033	22.201839	2.947413
H	20.530781	21.617303	3.869480
H	19.437890	22.005861	2.527093
H	20.472791	23.261855	3.225884

166

[**B**]

C	-1.811310	-2.813250	2.652569
C	-2.282045	-1.493331	2.868220
C	-3.662007	-1.198141	2.725278
C	-4.536461	-2.224738	2.356770
C	-4.079913	-3.517485	2.128314
C	-2.727140	-3.800926	2.278130
N	-1.365539	-0.433711	3.130123
C	-1.028973	-0.142014	4.382070
C	-1.545984	-1.009197	5.512660
C	-4.217136	0.197318	2.978183
C	-4.843974	0.802178	1.713510
C	-0.343609	-3.181721	2.822356
C	0.323819	-3.479535	1.471904
C	-0.221837	0.948642	4.766132
C	0.293044	2.022383	4.009408
C	0.985835	3.113669	4.800684
N	0.197609	2.134941	2.689213
C	0.654011	3.320926	2.042666
C	1.991076	3.417769	1.581644
C	2.369034	4.545934	0.847516
C	1.470617	5.572031	0.578791
C	0.166231	5.478368	1.049764
C	-0.266613	4.367062	1.779266
C	3.021713	2.334978	1.868173
C	3.380365	1.548275	0.599279
C	-1.702743	4.323819	2.283967
C	-2.715708	4.329682	1.129686
C	4.293990	2.899069	2.518511
C	-1.998774	5.474277	3.258538
C	-5.229996	0.208099	4.133290
C	-0.148646	-4.365175	3.782324
Ca	-0.609064	0.561006	1.125210
C	3.665191	-0.447764	5.395283
C	4.363789	-1.345816	4.698584
C	5.825116	-1.637172	4.875002
C	6.101284	-3.088882	5.292242
C	7.591221	-3.402529	5.433824
C	7.863129	-4.845102	5.855107

Ca	-0.640755	-0.025215	-2.349794	H	2.038894	3.193711	4.509036
N	0.117763	0.966066	-4.353866	H	0.531714	4.089465	4.599096
C	-0.213891	0.673971	-5.607317	H	0.938982	2.922881	5.875162
C	-1.023127	-0.413197	-5.993835	H	-0.037514	1.019608	5.832307
C	-1.553066	-1.477592	-5.237290	H	-2.640957	-1.035121	5.512315
N	-1.466307	-1.588314	-3.915244	H	-1.211309	-2.045377	5.394527
C	-1.939337	-2.768168	-3.270477	H	-1.207723	-0.645169	6.485343
C	-3.277803	-2.846200	-2.809600	H	2.577987	1.629973	2.579658
C	-3.672574	-3.968850	-2.075957	H	4.065717	3.500706	3.405546
C	-2.788960	-5.007691	-1.806875	H	4.953900	2.079812	2.826499
C	-1.483334	-4.932889	-2.277831	H	4.856143	3.534900	1.823892
C	-1.034547	-3.828343	-3.007890	H	3.806547	2.206945	-0.166797
C	-4.291900	-1.747055	-3.094980	H	4.118361	0.768712	0.825749
C	-4.645832	-0.962704	-1.823328	H	2.504531	1.057835	0.157760
C	0.402032	-3.805599	-3.512759	H	3.390323	4.622573	0.482347
C	1.414232	-3.805021	-2.357728	H	1.787136	6.442455	0.009367
C	1.028545	2.029332	-4.088124	H	-0.534103	6.286249	0.848426
C	0.549582	3.346731	-3.874425	H	-1.831665	3.385967	2.835363
C	1.458435	4.339127	-3.495615	H	-3.017375	5.386549	3.655968
C	2.812007	4.062597	-3.339661	H	-1.305550	5.476013	4.106795
C	3.276588	2.772290	-3.566072	H	-1.918958	6.449464	2.763057
C	2.409302	1.741198	-3.939028	H	-2.557499	3.488687	0.444978
C	-0.919525	3.706663	-4.051055	H	-3.739463	4.262764	1.517764
C	-1.594747	4.003946	-2.704349	H	-2.644619	5.252289	0.540923
C	2.971884	0.348335	-4.189668	H	-3.378009	0.837432	3.272083
C	3.587562	-0.257686	-2.919968	H	-6.116577	-0.392059	3.895463
C	0.312923	1.537586	-6.736058	H	-4.796648	-0.192828	5.056036
C	3.996896	0.343360	-5.334006	H	-5.567403	1.231812	4.336389
C	-1.117153	4.886759	-5.014560	H	-5.203769	1.819033	1.912696
C	-2.249851	-2.565954	-6.029190	H	-4.125608	0.853658	0.887369
C	-5.568439	-2.290481	-3.754800	H	-5.700105	0.209240	1.369801
C	0.688732	-4.971920	-4.471122	H	-5.596760	-2.006175	2.249471
H	0.514848	-0.432628	-0.510845	H	-4.775016	-4.301760	1.839010
H	-1.766431	0.963534	-0.712351	H	-2.373004	-4.813874	2.102256

H	0.166523	-2.316153	3.260098
H	-0.562870	-5.292905	3.370002
H	0.919519	-4.535051	3.962952
H	-0.631389	-4.189612	4.750208
H	0.282581	-2.618872	0.793973
H	1.380270	-3.740787	1.612903
H	-0.166863	-4.318873	0.965235
H	-3.296388	-2.662530	-5.720148
H	-1.781808	-3.539398	-5.847650
H	-2.223096	-2.362369	-7.102019
H	-1.208241	-0.481434	-7.059962
H	-0.019957	1.172369	-7.710187
H	1.408005	1.560423	-6.728806
H	-0.019223	2.575079	-6.622186
H	-3.831492	-1.045207	-3.799726
H	-5.346500	-2.872842	-4.656175
H	-6.229616	-1.463322	-4.040073
H	-6.131095	-2.939365	-3.073057
H	-5.087342	-1.619342	-1.064326
H	-5.369892	-0.169441	-2.048030
H	-3.764521	-0.489762	-1.374052
H	-4.694717	-4.030934	-1.710319
H	-3.117987	-5.873178	-1.237079
H	-0.794677	-5.750664	-2.076097
H	0.540026	-2.876567	-4.076633
H	1.706838	-4.895751	-4.872142
H	-0.006571	-4.982178	-5.317646
H	0.604959	-5.939585	-3.961767
H	1.263843	-2.953524	-1.684217
H	2.438937	-3.753082	-2.745703
H	1.333377	-4.718888	-1.756678
H	2.137874	-0.293672	-4.493872
H	4.880027	0.943948	-5.084753
H	3.572748	0.746891	-6.259909
H	4.338183	-0.679090	-5.537071
H	3.952661	-1.272963	-3.117891
H	2.861287	-0.313336	-2.100985
H	4.438081	0.337455	-2.566318
H	4.337505	2.559408	-3.453499
H	3.501601	4.850419	-3.046829
H	1.098179	5.350116	-3.320819
H	-1.423135	2.837267	-4.488851
H	-0.708334	5.817446	-4.603490
H	-2.185731	5.051241	-5.198093
H	-0.630824	4.711022	-5.980665
H	-1.549366	3.145884	-2.023371
H	-2.652635	4.255940	-2.850958
H	-1.112761	4.848677	-2.198301
H	2.603264	-0.285152	5.225669
H	4.137389	0.165527	6.161863
H	3.849605	-1.941163	3.940563
H	6.352856	-1.439429	3.928467
H	6.251627	-0.951007	5.619273
H	5.589056	-3.291414	6.243354
H	5.654186	-3.770869	4.553646
H	8.037276	-2.715978	6.167628
H	8.097025	-3.196850	4.479418
H	7.397560	-5.069632	6.822679
H	8.937656	-5.039309	5.949928
H	7.459965	-5.555086	5.122394

166

[TS-**BC**]

C	19.956321	16.515460	9.345596
C	19.813007	17.926225	9.356147
C	18.564547	18.503131	9.014016
C	17.499284	17.666919	8.667433
C	17.635313	16.283910	8.652976
C	18.860306	15.722583	8.990524

N	20.922801	18.774322	9.650092
C	21.122683	19.163793	10.904288
C	20.272705	18.580054	12.018816
C	18.346496	20.007835	9.000619
C	18.083161	20.510606	7.574193
C	21.262076	15.826423	9.711992
C	21.843231	15.064990	8.512533
C	22.050735	20.146385	11.327146
C	22.715568	21.193712	10.649101
C	23.395121	22.201794	11.562499
N	22.787464	21.347290	9.328031
C	23.248050	22.588940	8.797865
C	24.620965	22.822752	8.543134
C	25.013195	24.044332	7.985054
C	24.088260	25.034656	7.685671
C	22.740169	24.799800	7.932533
C	22.295122	23.591333	8.474855
C	25.692286	21.788822	8.850433
C	26.299123	21.228311	7.556702
C	20.811287	23.386501	8.743759
C	19.915516	23.984551	7.652561
C	26.805186	22.341118	9.753891
C	20.404321	23.946088	10.116722
C	17.212733	20.437947	9.943076
C	21.112385	14.876806	10.910465
Ca	22.638585	19.313004	8.071140
C	24.597169	17.935093	8.974653
C	24.754443	17.317029	7.724502
C	26.103674	17.331159	7.028346
C	27.094664	16.364800	7.690194
C	28.453728	16.327663	6.989043
C	29.438641	15.361201	7.644042
Ca	22.829260	18.415768	4.464574
N	23.425470	19.474245	2.445749
C	23.269862	19.050343	1.196104
C	22.687887	17.819982	0.829544
C	22.115687	16.797650	1.610363
N	22.066876	16.784769	2.940951
C	21.413363	15.703867	3.605253
C	20.040185	15.814204	3.941575
C	19.445138	14.793915	4.689586
C	20.165206	13.674261	5.089029
C	21.505980	13.562918	4.738977
C	22.151493	14.559314	4.000519
C	19.197675	17.003268	3.501100
C	18.791783	17.880659	4.694045
C	23.619047	14.380670	3.636393
C	24.512221	14.399376	4.884630
C	23.964202	20.769585	2.703684
C	23.080065	21.857494	2.922989
C	23.614354	23.092631	3.301847
C	24.983393	23.272686	3.460632
C	25.844715	22.208163	3.221397
C	25.363580	20.952962	2.837132
C	21.576618	21.731017	2.713557
C	20.775723	22.118845	3.963880
C	26.353954	19.829175	2.566576
C	27.076473	19.392462	3.847870
C	23.749603	19.909429	0.042851
C	27.378613	20.208748	1.486779
C	21.112405	22.559610	1.504632
C	21.490966	15.654932	0.835178
C	17.952601	16.568723	2.712963
C	23.866930	13.096707	2.830030
H	23.929555	17.966843	6.554762
H	21.820855	19.479357	6.043701
H	24.473328	22.002314	11.595483
H	23.272624	23.225438	11.198989

H	23.012386	22.141848	12.584110
H	22.142600	20.210940	12.406023
H	19.279882	19.046512	12.013535
H	20.114105	17.506258	11.893638
H	20.721266	18.762967	12.998330
H	25.212628	20.960272	9.383416
H	26.400753	22.792351	10.665925
H	27.493554	21.538522	10.045876
H	27.396424	23.109105	9.240985
H	26.762377	22.024635	6.962385
H	27.071301	20.482529	7.780001
H	25.539925	20.755114	6.922349
H	26.068369	24.223824	7.790622
H	24.412920	25.982466	7.262865
H	22.016842	25.575911	7.697161
H	20.638972	22.304474	8.765637
H	19.329527	23.804571	10.286374
H	20.937428	23.449356	10.932619
H	20.616507	25.021130	10.177314
H	20.197563	23.625647	6.657485
H	18.870892	23.705977	7.831611
H	19.952971	25.080663	7.642076
H	19.268889	20.479371	9.353735
H	16.243130	20.042278	9.617053
H	17.384171	20.089513	10.967575
H	17.130505	21.531416	9.967249
H	17.931365	21.595989	7.567373
H	18.924378	20.280586	6.911891
H	17.184467	20.046579	7.149261
H	16.541428	18.111655	8.405793
H	16.793271	15.649702	8.385966
H	18.970534	14.640404	8.983778
H	21.976992	16.604516	9.999374
H	20.430481	14.048280	10.683912
H	22.084263	14.442766	11.175666
H	20.723272	15.392873	11.794429
H	21.967351	15.716001	7.640016
H	22.820135	14.633518	8.763256
H	21.184346	14.245198	8.204373
H	20.407407	15.630296	0.998850
H	21.876625	14.687965	1.172258
H	21.673920	15.750243	-0.237421
H	22.644163	17.646512	-0.239952
H	23.270176	19.618937	-0.895123
H	24.832858	19.796350	-0.086609
H	23.561091	20.970641	0.225262
H	19.811560	17.613612	2.829007
H	18.212069	15.930174	1.861159
H	17.422355	17.448175	2.328182
H	17.249337	16.011467	3.343123
H	18.189178	17.318058	5.416157
H	18.199372	18.739816	4.356381
H	19.662047	18.270128	5.236085
H	18.396140	14.878502	4.962586
H	19.682421	12.889634	5.666306
H	22.064979	12.681166	5.044654
H	23.909618	15.228346	3.005781
H	24.916756	13.039070	2.517882
H	23.245047	13.054665	1.929410
H	23.649503	12.201069	3.424067
H	24.378945	15.322482	5.459755
H	25.570650	14.321883	4.607253
H	24.278744	13.561584	5.552520
H	25.786358	18.967560	2.197368
H	28.038061	21.017297	1.824463
H	26.891608	20.545615	0.565513
H	28.012830	19.347992	1.242141
H	27.749105	18.549250	3.648406

H	26.369881	19.082395	4.625315
H	27.678029	20.210107	4.262846
H	26.916805	22.354825	3.332742
H	25.376370	24.238806	3.766162
H	22.942966	23.929791	3.477041
H	21.359715	20.679953	2.489707
H	21.286333	23.629979	1.669316
H	20.038141	22.418294	1.333977
H	21.639668	22.273955	0.588108
H	21.061995	21.511780	4.829769
H	19.702791	21.974684	3.786801
H	20.927613	23.173016	4.224433
H	24.003352	17.457326	9.751778
H	25.343267	18.651347	9.317228
H	24.231563	16.366621	7.605397
H	25.988910	17.061889	5.969391
H	26.517620	18.346471	7.050476
H	27.227077	16.647523	8.742333
H	26.662981	15.352653	7.698706
H	28.884416	17.339370	6.979552
H	28.313138	16.048300	5.934671
H	29.627357	15.636001	8.689129
H	30.402617	15.354834	7.122184
H	29.049833	14.335333	7.638292

166

[**C**]

C	-2.221494	-2.583016	3.146236
C	-2.575764	-1.213661	3.239439
C	-3.928811	-0.824930	3.064241
C	-4.891753	-1.807801	2.818512
C	-4.551497	-3.153009	2.737296
C	-3.222831	-3.526760	2.895357
N	-1.580284	-0.210750	3.445904
C	-1.294894	0.159579	4.694268
C	-1.914808	-0.598803	5.853086
C	-4.366736	0.631548	3.128863
C	-4.905464	1.109943	1.772179
C	-0.787196	-3.067167	3.301837
C	-0.267858	-3.679696	1.993471
C	-0.483570	1.250847	5.062592
C	0.032378	2.342497	4.325884
C	0.508959	3.488740	5.203935
N	0.073498	2.432668	3.002456
C	0.473796	3.656265	2.386268
C	1.824160	3.860466	2.002610
C	2.168460	5.054134	1.360093
C	1.219977	6.031267	1.083025
C	-0.103111	5.818374	1.450375
C	-0.500199	4.645813	2.100008
C	2.916728	2.843812	2.303621
C	3.782192	2.532869	1.074752
C	-1.961069	4.484228	2.495227
C	-2.888068	4.516338	1.271668
C	3.813471	3.299970	3.466257
C	-2.390453	5.546809	3.518844
C	-5.405635	0.876600	4.233389
C	-0.626657	-4.076639	4.449247
Ca	-0.471908	0.634960	1.530887
C	1.488241	-0.645321	0.595658
C	2.410959	-1.585743	-0.192578
C	3.789751	-1.897082	0.422082
C	3.739331	-2.690094	1.727054
C	5.120852	-2.951349	2.330065
C	5.068621	-3.761321	3.623976
Ca	0.209101	-0.425028	-1.691634
N	0.954115	0.607935	-3.714160
C	0.496637	0.331288	-4.930478
C	-0.299208	-0.781937	-5.271234

C	-0.743775	-1.895350	-4.530238
N	-0.530897	-2.094405	-3.232243
C	-0.957063	-3.306634	-2.617897
C	-2.286309	-3.458032	-2.150285
C	-2.630928	-4.630764	-1.469054
C	-1.707756	-5.646802	-1.261004
C	-0.407575	-5.497992	-1.734150
C	-0.005533	-4.341683	-2.407861
C	-3.357743	-2.400723	-2.380344
C	-3.775633	-1.715066	-1.072218
C	1.415959	-4.222061	-2.943895
C	2.455808	-4.976890	-2.106950
C	1.813995	1.723998	-3.503233
C	1.283817	3.021006	-3.288661
C	2.162626	4.063504	-2.974566
C	3.533683	3.858109	-2.892068
C	4.050195	2.588284	-3.128227
C	3.216228	1.508121	-3.429407
C	-0.201265	3.329048	-3.422496
C	-0.826648	3.745197	-2.084703
C	3.818628	0.143700	-3.734591
C	5.025884	-0.202742	-2.855163
C	0.791628	1.258547	-6.098419
C	4.206094	0.027371	-5.218188
C	-0.459656	4.408673	-4.486081
C	-1.551393	-2.898692	-5.337603
C	-4.592464	-2.981913	-3.087841
C	1.510587	-4.681716	-4.408351
H	2.628157	-1.174841	-1.200618
H	-1.261459	0.686142	-0.493251
H	1.060832	4.247198	4.647410
H	-0.351344	3.976275	5.677852
H	1.143076	3.106654	6.010999
H	-0.356607	1.360848	6.135060
H	-3.001348	-0.455769	5.865777
H	-1.746933	-1.675556	5.760520
H	-1.514468	-0.262213	6.811974
H	2.427208	1.913871	2.617030
H	3.241666	3.462469	4.384576
H	4.581413	2.545096	3.675765
H	4.324271	4.238895	3.219350
H	4.398918	3.392222	0.786733
H	4.464603	1.703215	1.294419
H	3.177853	2.259856	0.204009
H	3.203744	5.222850	1.074497
H	1.510979	6.953444	0.585873
H	-0.846337	6.583130	1.235447
H	-2.070790	3.503815	2.971289
H	-3.421356	5.368595	3.848500
H	-1.744388	5.539187	4.402924
H	-2.349493	6.554577	3.087709
H	-2.637246	3.727640	0.554124
H	-3.931964	4.378330	1.577422
H	-2.822250	5.475716	0.744727
H	-3.483454	1.232870	3.369461
H	-6.337599	0.332284	4.038261
H	-5.035646	0.558640	5.214220
H	-5.653004	1.943350	4.295967
H	-5.188358	2.168278	1.819727
H	-4.160716	0.992945	0.976717
H	-5.795297	0.541449	1.475806
H	-5.930584	-1.511833	2.689337
H	-5.315859	-3.903594	2.550919
H	-2.953308	-4.578159	2.825978
H	-0.165417	-2.195854	3.539710
H	-1.190636	-4.996724	4.254624
H	0.427903	-4.354198	4.565954
H	-0.975482	-3.671789	5.405208

H	-0.339080	-2.972526	1.160299
H	0.782247	-3.976588	2.096458
H	-0.842597	-4.569132	1.712423
H	-1.483091	-3.910166	-4.931098
H	-1.227770	-2.913170	-6.382004
H	-2.610750	-2.613620	-5.327083
H	-0.589542	-0.806893	-6.317329
H	0.959999	0.686552	-7.015694
H	1.657251	1.897906	-5.914871
H	-0.071378	1.911868	-6.277793
H	-2.934473	-1.630609	-3.034703
H	-4.323934	-3.515280	-4.006056
H	-5.291784	-2.178875	-3.350515
H	-5.131528	-3.685830	-2.442513
H	-4.154878	-2.438475	-0.341993
H	-4.566939	-0.979505	-1.262544
H	-2.938218	-1.179870	-0.611310
H	-3.646728	-4.749111	-1.099350
H	-1.998293	-6.553720	-0.736055
H	0.308554	-6.298851	-1.572886
H	1.678675	-3.156282	-2.928119
H	2.545123	-4.606211	-4.766068
H	0.885696	-4.073585	-5.067328
H	1.194614	-5.727811	-4.506941
H	2.370328	-4.750842	-1.038723
H	3.465973	-4.703025	-2.432760
H	2.365144	-6.063372	-2.227827
H	3.036776	-0.603721	-3.549801
H	4.960047	0.779943	-5.480974
H	3.343873	0.171426	-5.875424
H	4.628237	-0.963101	-5.429418
H	5.309395	-1.251409	-3.003449
H	4.813425	-0.054778	-1.791322
H	5.903632	0.404033	-3.108557
H	5.124885	2.434642	-3.078025
H	4.198867	4.683991	-2.652022
H	1.760903	5.058336	-2.797168
H	-0.705858	2.413597	-3.750849
H	-0.056608	5.380396	-4.176257
H	-1.537428	4.535773	-4.644827
H	-0.000569	4.152117	-5.446645
H	-0.762830	2.938136	-1.347005
H	-1.889082	3.984155	-2.218791
H	-0.332093	4.628986	-1.665622
H	1.266669	-1.168936	1.549158
H	2.107908	0.223371	0.900661
H	1.894372	-2.542762	-0.373347
H	4.395818	-2.454146	-0.308226
H	4.313351	-0.945693	0.597384
H	3.121490	-2.154708	2.461354
H	3.237378	-3.653016	1.546062
H	5.619396	-1.989768	2.519881
H	5.746023	-3.476625	1.593614
H	4.476823	-3.245425	4.390417
H	6.071142	-3.928408	4.034590
H	4.609349	-4.743809	3.457982

168

[**C**1] scf done: -4071.120272 / Energies= -4069.557019 / Enthalpies= -4069.556075 / Free Energies= -4069.774684

C	-3.768302	-0.717346	2.744794
C	-2.475630	-1.200899	3.072701
C	-2.191237	-2.584659	2.966008
C	-3.198320	-3.450393	2.528159
C	-4.467925	-2.983703	2.210062
C	-4.742431	-1.625436	2.321110
N	-1.456363	-0.273977	3.445932
C	-1.329377	0.045732	4.733342

C	-2.149792	-0.700738	5.768368
C	-0.825230	-3.156472	3.316882
C	-0.109196	-3.689546	2.068187
C	-4.124995	0.759862	2.841342
C	-4.425944	1.354614	1.457616
C	-0.510031	1.069593	5.242982
C	0.235977	2.081016	4.598861
C	0.659222	3.212609	5.521348
N	0.516618	2.125640	3.301508
C	1.208734	3.259025	2.782802
C	2.612822	3.192884	2.584590
C	3.261337	4.294734	2.017876
C	2.560477	5.434374	1.637555
C	1.185322	5.485335	1.825532
C	0.488797	4.416860	2.397957
C	3.419665	1.968991	3.002674
C	4.604295	1.679434	2.073126
C	-1.019364	4.526472	2.574106
C	-1.741609	4.508416	1.218602
C	3.924105	2.075589	4.451347
C	-1.423903	5.772512	3.375500
C	-0.905959	-4.257594	4.385136
C	-5.299229	1.012356	3.798788
Ca	-0.169463	0.539140	1.626557
Ca	0.174534	-0.523919	-1.680167
N	-0.544994	-2.287427	-3.165521
C	-1.225253	-3.362517	-2.518460
C	-2.616368	-3.267433	-2.264057
C	-3.247806	-4.302973	-1.568124
C	-2.546356	-5.424783	-1.145497
C	-1.185012	-5.520036	-1.413606
C	-0.499833	-4.504902	-2.088517
C	-3.451639	-2.091518	-2.752288
C	-3.988669	-1.249674	-1.587008
C	0.984109	-4.673687	-2.391130
C	1.764896	-5.317303	-1.237213
C	-0.507670	-2.258391	-4.492999
C	-1.184249	-3.345241	-5.314318
C	0.072602	-1.242676	-5.283070
C	0.548799	0.045145	-4.975216
C	0.887011	0.895687	-6.186719
N	0.661805	0.564398	-3.753404
C	1.035761	1.937046	-3.631105
C	2.396772	2.296830	-3.460105
C	2.723019	3.643060	-3.270883
C	1.745033	4.629784	-3.243350
C	0.413727	4.272713	-3.421116
C	0.033976	2.942514	-3.626101
C	3.517295	1.266494	-3.495929
C	4.315776	1.252683	-2.185553
C	-1.437821	2.627810	-3.862941
C	-2.293991	2.922617	-2.622808
C	-1.996216	3.384973	-5.078584
C	4.469048	1.485158	-4.682756
C	-4.607140	-2.551038	-3.655420
C	1.219294	-5.488078	-3.674388
C	1.725190	-0.679668	0.439123
C	2.531468	-1.643549	-0.445042
C	3.981041	-1.960662	-0.027903
C	4.112725	-2.696941	1.304438
C	5.560317	-3.034054	1.667347
C	5.691729	-3.764119	3.002484
H	2.607946	-1.260416	-1.485092
H	-1.147066	0.593413	-0.324906
H	1.519107	3.769116	5.144882
H	-0.169639	3.923558	5.628834
H	0.888604	2.832227	6.521194
H	-0.556501	1.179878	6.321283

H	-3.221018	-0.536664	5.607739	H	0.006902	-2.909737	1.306951
H	-1.988630	-1.780745	5.693542	H	0.890210	-4.062231	2.324752
H	-1.900748	-0.381007	6.782687	H	-0.670657	-4.510325	1.607796
H	2.745364	1.103687	2.961986	H	-1.366006	-4.259197	-4.747222
H	3.102186	2.132132	5.169289	H	-0.588928	-3.586538	-6.200423
H	4.530885	1.198304	4.707949	H	-2.154640	-2.977518	-5.670793
H	4.549921	2.967649	4.579161	H	0.042969	-1.454856	-6.347087
H	5.408274	2.415530	2.195168	H	1.031334	0.277115	-7.075851
H	5.030547	0.697475	2.307334	H	1.782789	1.499922	-6.026232
H	4.307711	1.678466	1.019449	H	0.070239	1.596891	-6.394909
H	4.336796	4.261369	1.867755	H	-2.802086	-1.447852	-3.355647
H	3.085631	6.276779	1.194197	H	-4.251019	-3.170013	-4.485838
H	0.638033	6.374899	1.521786	H	-5.129387	-1.682753	-4.075462
H	-1.354477	3.649836	3.138849	H	-5.344971	-3.139157	-3.096458
H	-2.504705	5.770946	3.561592	H	-4.629057	-1.845936	-0.927116
H	-0.911967	5.814274	4.342859	H	-4.582865	-0.408378	-1.965078
H	-1.185758	6.696347	2.834926	H	-3.179319	-0.837570	-0.974597
H	-1.521213	3.598908	0.648394	H	-4.313191	-4.230515	-1.362980
H	-2.828123	4.561497	1.357863	H	-3.057751	-6.225155	-0.616094
H	-1.440516	5.361614	0.598842	H	-0.642464	-6.405491	-1.092735
H	-3.252468	1.283923	3.246178	H	1.396452	-3.672562	-2.565285
H	-6.223132	0.546087	3.435743	H	2.294327	-5.612988	-3.854954
H	-5.099533	0.615730	4.800275	H	0.787922	-5.002920	-4.553438
H	-5.488689	2.088513	3.894544	H	0.773801	-6.486960	-3.586772
H	-4.637756	2.428096	1.537683	H	1.552467	-4.835824	-0.277190
H	-3.583751	1.222280	0.769087	H	2.842259	-5.244142	-1.426380
H	-5.301834	0.876322	1.002394	H	1.530456	-6.383586	-1.132529
H	-5.736390	-1.259540	2.072818	H	3.054836	0.280566	-3.621335
H	-5.239214	-3.674772	1.878869	H	4.975777	2.455341	-4.612316
H	-2.983008	-4.512313	2.437051	H	3.941370	1.454946	-5.641460
H	-0.220532	-2.340618	3.729724	H	5.241735	0.706740	-4.700631
H	-1.446249	-5.137365	4.015730	H	5.070067	0.456878	-2.201147
H	0.100981	-4.585158	4.670871	H	3.664923	1.087554	-1.320792
H	-1.417612	-3.912054	5.289972	H	4.839431	2.202970	-2.026656

H	3.766243	3.923774	-3.143832
H	2.018758	5.670487	-3.088646
H	-0.351654	5.045740	-3.409721
H	-1.519403	1.556141	-4.075100
H	-2.015902	4.467262	-4.902396
H	-3.025355	3.066378	-5.284357
H	-1.400304	3.209589	-5.980716
H	-1.964418	2.336715	-1.757818
H	-3.345496	2.676030	-2.816728
H	-2.246394	3.984968	-2.353182
H	1.687158	-1.156362	1.441793
H	2.354598	0.221586	0.594119
H	1.992747	-2.600375	-0.527600
H	4.454940	-2.566280	-0.816211
H	4.547343	-1.020004	0.021054
H	3.674320	-2.088340	2.107295
H	3.524410	-3.626468	1.268034
H	6.150941	-2.106918	1.697138
H	6.001825	-3.647759	0.868886
H	5.290153	-3.160029	3.825742
H	6.738537	-3.990476	3.235923
H	5.140860	-4.712945	2.991085
H	1.452419	2.911067	-0.180250
H	0.912567	2.785262	-0.676774

168

[TS-**C1C2**] scf done: -4071.100274 / Energies= -4069.537193 / Enthalpies= -4069.536249 / Free Energies= -4069.749941

C	-3.966611	-0.653117	2.988912
C	-2.654769	-1.093905	3.294910
C	-2.368837	-2.481918	3.346138
C	-3.415329	-3.391670	3.163314
C	-4.711905	-2.965272	2.900427
C	-4.972710	-1.604359	2.796845
N	-1.619802	-0.134289	3.514480
C	-1.439243	0.321664	4.752858
C	-2.276919	-0.221613	5.898285
C	-0.964707	-3.013656	3.602831
C	-0.564258	-4.088148	2.581352
C	-4.308529	0.822721	2.842872
C	-4.672443	1.158816	1.387863
C	-0.559553	1.357569	5.127345
C	0.105041	2.350204	4.375744
C	0.659831	3.481814	5.226461
N	0.213731	2.377799	3.051702
C	0.737004	3.544773	2.418123
C	2.091743	3.583206	2.000329
C	2.557091	4.718543	1.330558
C	1.721422	5.795839	1.058985
C	0.394585	5.749279	1.468193
C	-0.119106	4.642794	2.151669
C	3.058760	2.447318	2.306129
C	3.959420	2.089847	1.117201
C	-1.575715	4.665252	2.595690
C	-2.534974	4.667099	1.396807
C	3.924044	2.758777	3.538686
C	-1.876436	5.859887	3.514270
C	-0.797594	-3.567027	5.026979
C	-5.432214	1.263170	3.792122
Ca	-0.412299	0.590341	1.600256
Ca	0.271301	-0.459609	-1.755128
N	-0.351221	-2.150365	-3.317004
C	-0.505745	-3.443053	-2.738400
C	-1.714378	-3.823326	-2.104427
C	-1.766933	-5.054526	-1.440075
C	-0.673274	-5.909618	-1.407751
C	0.500602	-5.544801	-2.060142

C	0.611344	-4.323535	-2.730009
C	-2.969002	-2.962633	-2.157671
C	-3.404456	-2.480745	-0.768491
C	1.887521	-3.977695	-3.487429
C	3.163993	-4.526229	-2.839181
C	-0.717685	-1.924423	-4.575525
C	-1.498135	-2.970832	-5.353447
C	-0.456938	-0.742948	-5.296737
C	0.293881	0.406507	-4.974202
C	0.477229	1.383737	-6.122692
N	0.806015	0.675985	-3.777201
C	1.620546	1.833747	-3.603452
C	3.031176	1.683559	-3.584840
C	3.826186	2.808323	-3.348104
C	3.261198	4.057607	-3.116461
C	1.878878	4.194417	-3.127751
C	1.038762	3.104498	-3.375411
C	3.682916	0.336035	-3.862269
C	4.924993	0.070632	-3.002472
C	-0.468326	3.318005	-3.377087
C	-0.998463	3.569894	-1.959013
C	-0.898204	4.458553	-4.312069
C	4.032756	0.180404	-5.351208
C	-4.128723	-3.704612	-2.842709
C	1.814548	-4.457271	-4.947095
C	1.440668	-1.145992	0.819746
C	2.471268	-1.680252	-0.176536
C	3.756293	-2.273448	0.422834
C	3.534015	-3.507668	1.295241
C	4.830875	-4.080887	1.869266
C	4.609196	-5.314165	2.742427
H	2.802332	-0.861617	-0.853683
H	-1.264611	0.225347	-0.368574
H	1.367786	4.109970	4.683990
H	-0.157848	4.123934	5.575362
H	1.150612	3.076953	6.117531
H	-0.512981	1.525617	6.199185
H	-3.212831	0.346672	5.969511
H	-2.551972	-1.268864	5.762767
H	-1.753124	-0.109033	6.851465
H	2.456364	1.563617	2.551757
H	3.318576	2.922437	4.434917
H	4.611739	1.928774	3.744075
H	4.526060	3.660389	3.370276
H	4.692043	2.879393	0.911133
H	4.527335	1.178044	1.339123
H	3.376418	1.923455	0.206477
H	3.595407	4.759301	1.012726
H	2.104352	6.667541	0.533978
H	-0.258360	6.594520	1.261254
H	-1.766301	3.752291	3.169370
H	-2.902280	5.799067	3.897910
H	-1.194285	5.893759	4.370149
H	-1.781566	6.812427	2.979016
H	-2.379036	3.795183	0.752597
H	-3.577676	4.656539	1.737489
H	-2.396116	5.561745	0.777964
H	-3.413464	1.397506	3.106067
H	-6.379887	0.765238	3.554264
H	-5.191926	1.035485	4.836264
H	-5.599385	2.344059	3.710648
H	-4.873897	2.231125	1.276741
H	-3.866289	0.888589	0.695552
H	-5.571716	0.616006	1.071979
H	-5.982687	-1.271428	2.567882
H	-5.511264	-3.689805	2.765003
H	-3.208545	-4.457267	3.227117
H	-0.270847	-2.170813	3.497287

H	-1.500756	-4.388902	5.210805
H	0.217979	-3.956831	5.169062
H	-0.970302	-2.800566	5.788114
H	-0.736654	-3.760453	1.550923
H	0.497317	-4.338691	2.688441
H	-1.131573	-5.015142	2.727885
H	-1.372532	-3.974847	-4.942895
H	-1.204667	-2.978037	-6.407236
H	-2.567707	-2.728974	-5.316302
H	-0.839203	-0.753046	-6.312864
H	0.410102	0.873386	-7.087007
H	1.433986	1.908116	-6.063692
H	-0.309616	2.147455	-6.096328
H	-2.740416	-2.075407	-2.759251
H	-3.844379	-4.089889	-3.827407
H	-4.985702	-3.032435	-2.972241
H	-4.467284	-4.555818	-2.239732
H	-3.629032	-3.320676	-0.101881
H	-4.309668	-1.866601	-0.844706
H	-2.632739	-1.867259	-0.292784
H	-2.689421	-5.349749	-0.945385
H	-0.736163	-6.861726	-0.886446
H	1.346671	-6.226616	-2.047866
H	1.962221	-2.883029	-3.513978
H	2.739669	-4.204948	-5.480255
H	0.982784	-3.997212	-5.486335
H	1.685357	-5.546053	-4.989929
H	3.228083	-4.276816	-1.775258
H	4.044907	-4.107841	-3.340013
H	3.232940	-5.616924	-2.932085
H	2.939026	-0.436521	-3.628281
H	4.741555	0.956991	-5.664806
H	3.144680	0.258344	-5.985541
H	4.495749	-0.796305	-5.540090
H	5.254701	-0.967532	-3.128561
H	4.728500	0.240072	-1.938196
H	5.767277	0.710539	-3.291649
H	4.908260	2.706117	-3.343017
H	3.896292	4.919624	-2.927416
H	1.439260	5.171018	-2.939850
H	-0.937574	2.397457	-3.741268
H	-0.550827	5.432214	-3.946794
H	-1.992198	4.505629	-4.374541
H	-0.503315	4.326750	-5.325392
H	-0.771332	2.729082	-1.294175
H	-2.087391	3.703257	-1.974570
H	-0.550430	4.468644	-1.519796
H	0.767813	-1.954253	1.142546
H	1.975792	-0.804732	1.725594
H	2.009525	-2.452691	-0.815607
H	4.452859	-2.523018	-0.390960
H	4.249392	-1.491338	1.018588
H	2.853742	-3.252593	2.119716
H	3.020529	-4.283937	0.707757
H	5.340764	-3.303226	2.456150
H	5.513133	-4.334280	1.044942
H	3.960285	-5.084003	3.596687
H	5.555125	-5.700961	3.138651
H	4.131594	-6.121606	2.173575
H	1.238432	0.229691	0.178342
H	1.210535	1.073019	-0.261147

168

[**C2**] scf done: -4071.174449 / Energies= -4069.606568 / Enthalpies= -4069.605623 / Free Energies= -4069.831178

C	-4.009204	-0.985792	2.857722
C	-2.643784	-1.335261	3.015153
C	-2.224701	-2.674548	2.813346

C	-3.175233	-3.626247	2.432569
C	-4.512825	-3.288025	2.261382
C	-4.919474	-1.976998	2.479602
N	-1.686267	-0.313850	3.280482
C	-1.352543	-0.030891	4.535587
C	-1.938125	-0.855784	5.664247
C	-0.775741	-3.101585	3.007066
C	-0.106025	-3.452262	1.670889
C	-4.510705	0.430451	3.107672
C	-5.076476	1.071008	1.831898
C	-0.493875	1.016369	4.924398
C	0.091276	2.050773	4.167527
C	0.834350	3.106992	4.961173
N	0.017097	2.161493	2.844493
C	0.553752	3.317007	2.205394
C	1.901471	3.332269	1.766128
C	2.362850	4.439896	1.049133
C	1.534839	5.522218	0.773764
C	0.218222	5.505977	1.219044
C	-0.295795	4.419392	1.933021
C	2.853383	2.179523	2.055414
C	3.164518	1.376327	0.784451
C	-1.740903	4.461018	2.411615
C	-2.729777	4.513072	1.237539
C	4.157164	2.649563	2.718106
C	-1.991402	5.634490	3.371261
C	-0.641506	-4.274123	3.990615
C	-5.555431	0.476736	4.233245
Ca	-0.876447	0.638290	1.273658
Ca	-0.917051	0.115721	-2.221643
N	-1.910237	-1.273538	-3.854581
C	-2.606945	-2.348185	-3.230989
C	-3.958619	-2.189736	-2.834327
C	-4.569304	-3.213908	-2.104456

C	-3.885280	-4.379099	-1.777208
C	-2.567235	-4.537174	-2.189939
C	-1.908146	-3.542126	-2.917457
C	-4.761059	-0.945996	-3.191967
C	-5.082584	-0.100483	-1.951553
C	-0.472436	-3.773069	-3.370420
C	0.495120	-3.874671	-2.182429
C	-1.908232	-1.160378	-5.178663
C	-2.718713	-2.134123	-6.010441
C	-1.189769	-0.186969	-5.900537
C	-0.280576	0.794762	-5.459326
C	0.404985	1.594832	-6.548835
N	0.013879	1.042201	-4.186716
C	1.013453	2.006470	-3.867577
C	2.355471	1.588512	-3.679128
C	3.299721	2.527644	-3.253673
C	2.948663	3.852016	-3.017483
C	1.633613	4.256629	-3.216265
C	0.651532	3.359213	-3.645339
C	2.795632	0.154396	-3.942786
C	3.252903	-0.549166	-2.656437
C	-0.769672	3.857664	-3.870651
C	-1.464897	4.203573	-2.545997
C	-0.819018	5.059893	-4.825705
C	3.897886	0.078688	-5.010420
C	-6.054135	-1.291612	-3.946648
C	-0.348179	-5.015622	-4.265225
C	5.118888	-3.695145	2.592890
C	6.389444	-2.958984	2.168576
C	7.409717	-2.751257	3.293542
C	8.010124	-4.039983	3.859542
C	9.084924	-3.790372	4.919256
C	9.680843	-5.076983	5.486934
H	6.112317	-1.977245	1.760597

H	-1.972958	1.164436	-0.572997
H	1.883953	3.160284	4.652300
H	0.408003	4.099872	4.781992
H	0.798813	2.902407	6.033554
H	-0.314121	1.080364	5.991661
H	-3.031557	-0.787028	5.664500
H	-1.694442	-1.916536	5.544112
H	-1.570798	-0.522742	6.637551
H	2.353218	1.504735	2.759442
H	3.966422	3.244978	3.618124
H	4.767960	1.785848	3.006968
H	4.759458	3.263209	2.037736
H	3.634374	2.009101	0.022319
H	3.848684	0.548189	1.008414
H	2.260190	0.946307	0.337701
H	3.392958	4.455719	0.701652
H	1.915684	6.375993	0.218689
H	-0.426763	6.357175	1.011458
H	-1.932007	3.536531	2.967600
H	-3.018850	5.602450	3.753970
H	-1.311419	5.609163	4.229824
H	-1.855384	6.599824	2.868784
H	-2.607422	3.657524	0.563483
H	-3.763076	4.509246	1.605809
H	-2.593220	5.424384	0.642848
H	-3.653692	1.032183	3.429802
H	-6.465243	-0.070888	3.959148
H	-5.172356	0.038544	5.161296
H	-5.844295	1.513751	4.443684
H	-5.402148	2.099470	2.030312
H	-4.334237	1.101435	1.026114
H	-5.944401	0.513942	1.459114
H	-5.968551	-1.716160	2.357393
H	-5.235248	-4.044054	1.964178

H	-2.860954	-4.654342	2.268355
H	-0.233673	-2.250475	3.434724
H	-1.096392	-5.189423	3.593645
H	0.416812	-4.489700	4.180480
H	-1.120771	-4.057820	4.951955
H	-0.107260	-2.604126	0.976243
H	0.937515	-3.751664	1.829412
H	-0.623886	-4.282954	1.176647
H	-2.461378	-3.169621	-5.764765
H	-2.558400	-1.978866	-7.079757
H	-3.788803	-2.022100	-5.802713
H	-1.317200	-0.235180	-6.976149
H	0.069329	1.290069	-7.542634
H	1.491965	1.469475	-6.495853
H	0.210356	2.666057	-6.429047
H	-4.143113	-0.334223	-3.859103
H	-5.859446	-1.913652	-4.827369
H	-6.554668	-0.375395	-4.282295
H	-6.760129	-1.836357	-3.308387
H	-5.684014	-0.669721	-1.232732
H	-5.651518	0.794978	-2.231886
H	-4.174300	0.229060	-1.433544
H	-5.603137	-3.096346	-1.788004
H	-4.379046	-5.162170	-1.207378
H	-2.037494	-5.455146	-1.944583
H	-0.172477	-2.906351	-3.969539
H	0.679200	-5.117933	-4.635365
H	-1.015108	-4.958425	-5.132422
H	-0.595116	-5.932828	-3.717104
H	0.464834	-2.979081	-1.551003
H	1.525659	-4.003322	-2.535867
H	0.252525	-4.733720	-1.545409
H	1.927801	-0.391496	-4.328945
H	4.820749	0.564978	-4.672006

H	3.592423	0.562399	-5.944739
H	4.139371	-0.967489	-5.234706
H	3.531769	-1.589223	-2.866371
H	2.467022	-0.557264	-1.892198
H	4.128209	-0.051415	-2.221508
H	4.331435	2.214590	-3.108296
H	3.697401	4.566924	-2.685410
H	1.362653	5.294130	-3.035964
H	-1.335572	3.044240	-4.338792
H	-0.336603	5.942992	-4.390141
H	-1.859951	5.328417	-5.042591
H	-0.319028	4.845570	-5.776874
H	-1.528510	3.337357	-1.876722
H	-2.487239	4.557926	-2.728569
H	-0.923990	4.991579	-2.008865
H	5.330726	-4.718570	2.922350
H	4.620298	-3.176114	3.421632
H	6.869155	-3.504902	1.343182
H	8.225969	-2.117825	2.916701
H	6.937989	-2.182900	4.109724
H	7.217533	-4.663206	4.296951
H	8.442466	-4.630445	3.037143
H	8.655032	-3.193546	5.736513
H	9.885892	-3.175238	4.484488
H	8.908882	-5.695943	5.960843
H	10.447583	-4.866388	6.241414
H	10.147302	-5.679613	4.697684
H	4.405226	-3.758365	1.763305
H	0.169145	-0.427881	-0.371359

184

[**D**]

C	2.206047	2.655963	-3.102787
C	2.434382	1.285276	-3.378574
C	3.765266	0.799854	-3.476612
C	4.826838	1.686091	-3.276163
C	4.607292	3.025528	-2.975008
C	3.303688	3.497657	-2.892357
N	1.355978	0.359127	-3.499564
C	0.798912	0.146450	-4.690183
C	1.114726	1.082571	-5.843884
C	4.059590	-0.646300	-3.848181
C	4.456827	-0.770929	-5.328133
C	0.807458	3.249743	-3.042984
C	0.642386	4.448628	-3.989910
C	-0.094065	-0.900013	-5.006231
C	-0.492767	-2.069166	-4.314890
C	-1.215238	-3.083187	-5.187036
N	-0.290390	-2.313010	-3.025303
C	-0.604563	-3.588264	-2.471499
C	-1.925082	-3.926329	-2.082436
C	-2.154988	-5.170281	-1.484522
C	-1.125405	-6.076144	-1.268184
C	0.168554	-5.738739	-1.649527
C	0.454140	-4.507568	-2.244946
C	-3.109051	-2.991694	-2.285183
C	-4.241098	-3.650166	-3.089206
C	1.873338	-4.196822	-2.696508
C	2.117355	-4.706108	-4.126027
C	-3.649441	-2.478948	-0.942711
C	2.948593	-4.742203	-1.749829
C	5.138269	-1.281126	-2.962433
C	0.425375	3.651265	-1.613097
Ca	0.323554	-0.539985	-1.548333
C	-1.410226	0.733084	-0.106712
C	-2.029289	0.848862	-1.508351
C	-3.407776	1.516166	-1.582845
C	-3.977289	1.625296	-2.996634
C	-5.357476	2.281721	-3.047929

C	-5.920550	2.389788	-4.463217	C	2.268697	-1.623749	1.736234
Ca	0.054211	0.321896	1.939806	C	3.528902	-1.596272	0.911479
N	0.240569	2.375550	3.176757	C	4.575636	-2.486410	1.600943
C	0.624812	3.623324	2.600881	C	5.917009	-2.553937	0.876209
C	1.980741	3.915234	2.313774	C	6.939595	-3.413656	1.616702
C	2.302986	5.141433	1.722379	H	1.221547	-0.805518	0.335342
C	1.324809	6.078497	1.417048	H	2.007376	3.203842	5.338683
C	-0.006425	5.781699	1.687322	H	0.686818	4.312422	4.991695
C	-0.382150	4.565715	2.265115	H	0.593722	3.234408	6.402102
C	3.104006	2.934303	2.611805	H	-0.046149	1.201317	6.292431
C	4.241755	3.561422	3.431265	H	-1.092602	-0.560170	6.957455
C	-1.850502	4.291608	2.554508	H	-2.689668	-0.038295	6.418639
C	-2.275253	4.887066	3.906497	H	-2.073185	-1.604131	5.899509
C	0.269347	2.235716	4.499479	H	2.681412	2.117745	3.207505
C	0.915910	3.309262	5.360857	H	3.869110	4.046595	4.339611
C	-0.248999	1.138013	5.228157	H	4.965792	2.792395	3.726948
C	-1.159839	0.105234	4.900313	H	4.787503	4.317210	2.854005
C	-1.783386	-0.572360	6.109536	H	4.061309	3.110339	0.655255
N	-1.515996	-0.259139	3.672069	H	4.476903	1.628888	1.532411
C	-2.643041	-1.119196	3.508455	H	2.893493	1.793621	0.747876
C	-3.952988	-0.572893	3.505001	H	3.344135	5.367013	1.503107
C	-5.042248	-1.419862	3.281126	H	1.595691	7.031562	0.969053
C	-4.875063	-2.783071	3.070736	H	-0.772063	6.512884	1.442310
C	-3.592178	-3.314728	3.083278	H	-1.966360	3.203776	2.624483
C	-2.468139	-2.510051	3.297741	H	-3.336459	4.686681	4.100012
C	-4.221967	0.907986	3.730456	H	-1.699208	4.462768	4.734601
C	-5.141214	1.150864	4.937703	H	-2.130544	5.974840	3.915718
C	-1.100939	-3.175740	3.336414	H	-2.468617	4.423858	0.460886
C	-0.970815	-4.150812	4.517306	H	-3.804619	4.447327	1.625130
C	-0.777763	-3.896752	2.021231	H	-2.815893	5.888537	1.396985
C	-4.817295	1.559947	2.474381	H	-3.264476	1.397264	3.934468
C	3.662862	2.331145	1.314921	H	-6.149965	0.758324	4.761002
C	-2.781245	4.793202	1.443511	H	-4.757600	0.673124	5.845483
C	2.144601	-0.948376	2.912419	H	-5.236522	2.225610	5.134581

H	-5.012844	2.624718	2.649968
H	-4.137299	1.473368	1.620803
H	-5.768806	1.090397	2.195947
H	-6.045767	-0.999951	3.275546
H	-5.736498	-3.425149	2.903833
H	-3.456463	-4.383384	2.931851
H	-0.355404	-2.387715	3.487351
H	-1.679517	-4.982284	4.423102
H	0.039629	-4.576283	4.554380
H	-1.163962	-3.656323	5.474913
H	-0.814346	-3.217861	1.161910
H	0.221323	-4.347435	2.060252
H	-1.489532	-4.704324	1.819688
H	-1.118084	-4.100323	-4.800986
H	-0.837122	-3.056016	-6.212555
H	-2.285899	-2.847201	-5.226456
H	-0.442475	-0.874276	-6.033907
H	0.858386	0.635819	-6.807382
H	2.169730	1.368043	-5.856222
H	0.537602	2.009549	-5.739096
H	-2.759427	-2.125878	-2.858192
H	-3.882731	-4.057104	-4.040466
H	-5.028967	-2.918254	-3.304631
H	-4.703815	-4.472933	-2.531494
H	-4.025323	-3.302124	-0.324500
H	-4.476067	-1.775798	-1.099877
H	-2.877228	-1.965085	-0.361223
H	-3.166844	-5.432620	-1.184350
H	-1.328142	-7.039850	-0.807380
H	0.972236	-6.451225	-1.484795
H	1.973338	-3.104698	-2.725616
H	3.136465	-4.467471	-4.454876
H	1.420227	-4.253213	-4.837508
H	1.991535	-5.794969	-4.175242

H	2.757203	-4.454732	-0.710083
H	3.932647	-4.353563	-2.035501
H	3.014504	-5.836131	-1.788068
H	3.131914	-1.212231	-3.708892
H	5.367734	-0.196057	-5.537329
H	3.668836	-0.403150	-5.992514
H	4.652524	-1.818714	-5.588012
H	5.233144	-2.349954	-3.189125
H	4.899534	-1.175793	-1.899496
H	6.124042	-0.830101	-3.128178
H	5.847603	1.321434	-3.361993
H	5.447019	3.697954	-2.816771
H	3.130048	4.547932	-2.669516
H	0.107398	2.474483	-3.374456
H	1.247338	5.303743	-3.666413
H	-0.403902	4.777149	-4.007371
H	0.940609	4.201771	-5.014648
H	0.475201	2.800874	-0.924239
H	-0.597059	4.046226	-1.580176
H	1.094267	4.425590	-1.222466
H	1.446099	-1.259662	3.687706
H	2.852492	-0.168075	3.186338
H	1.636990	-2.495520	1.576932
H	3.333477	-1.954897	-0.105487
H	3.912652	-0.573562	0.825127
H	4.730800	-2.114154	2.621956
H	4.169783	-3.503276	1.711204
H	6.314269	-1.536288	0.751538
H	5.769500	-2.950120	-0.137751
H	7.135458	-3.018900	2.621358
H	7.895247	-3.450907	1.081319
H	6.581443	-4.444245	1.731356
H	-2.164173	0.217481	0.523401
H	-1.379234	1.765413	0.295896

H	-2.143024	-0.148385	-1.978411
H	-1.360126	1.415013	-2.182686
H	-4.102026	0.950208	-0.945810
H	-3.333100	2.517443	-1.135526
H	-3.281110	2.197544	-3.628771
H	-4.036934	0.622399	-3.446400
H	-5.298093	3.283538	-2.598797
H	-6.053621	1.708825	-2.418582
H	-5.261537	2.985332	-5.107317
H	-6.908403	2.864931	-4.468244
H	-6.025097	1.399868	-4.924628

184

[TS-**DE**]

C	24.456856	21.899686	3.145105
C	24.685240	20.528724	2.871514
C	26.015761	20.042877	2.771150
C	27.077485	20.929506	2.970665
C	26.858062	22.268860	3.271877
C	25.554712	22.741468	3.354537
N	23.603932	19.601922	2.752542
C	23.049385	19.389866	1.559029
C	23.366860	20.326015	0.405941
C	26.310992	18.596983	2.399029
C	26.708167	18.473215	0.918914
C	23.058492	22.493903	3.204403
C	22.893750	23.692665	2.257301
C	22.157375	18.343961	1.241497
C	21.758789	17.175866	1.933788
C	21.037181	16.160783	1.062533
N	21.960755	16.935234	3.225096
C	21.647020	15.656743	3.778158
C	20.326262	15.318257	4.165202
C	20.096358	14.073820	4.762204
C	21.126181	13.168312	4.978456
C	22.420030	13.505627	4.597101
C	22.705842	14.737315	4.002403
C	19.142043	16.252432	3.962218
C	18.010207	15.593570	3.158168
C	24.125325	15.046997	3.550783
C	24.368626	14.537681	2.121112
C	18.601570	16.764908	5.304722
C	25.200247	14.501370	4.497619
C	27.390162	17.962612	3.284502
C	22.676921	22.895768	4.634247
Ca	22.585805	18.695218	4.701758
C	20.860720	19.963183	6.132816
C	20.227271	20.088505	4.735114
C	18.847345	20.758239	4.667573
C	18.275868	20.868654	3.252999
C	16.895047	21.525169	3.200767
C	16.331936	21.633135	1.785075
Ca	22.314316	19.563285	8.215377
N	22.487817	21.623030	9.424399
C	22.875342	22.867926	8.848467
C	24.231804	23.158920	8.561085
C	24.554036	24.385149	7.969482
C	23.576288	25.322726	7.663877
C	22.244776	25.026419	7.933762
C	21.869031	23.810707	8.511690
C	25.354556	22.177348	8.859385
C	26.492733	22.804565	9.678314
C	20.400939	23.536042	8.801614
C	19.976143	24.131041	10.153717
C	22.519473	21.480168	10.746788
C	23.168583	22.552272	11.607902
C	21.998780	20.384403	11.475164
C	21.090881	19.349916	11.147273
C	20.469270	18.670550	12.356496

N	20.730828	18.987351	9.919686	H	26.723480	20.868815	7.778973
C	19.607267	18.125177	9.756003	H	25.142195	21.042367	6.994293
C	18.297020	18.670587	9.751997	H	25.595424	24.610711	7.751051
C	17.208038	17.823598	9.528051	H	23.847688	26.276080	7.216676
C	17.376017	16.460386	9.317782	H	21.479466	25.758207	7.689192
C	18.659294	15.929394	9.330426	H	20.285957	22.448179	8.872463
C	19.783360	16.734207	9.545101	H	18.915071	23.930157	10.347638
C	18.028909	20.151513	9.977752	H	20.552811	23.706377	10.981114
C	17.109953	20.394784	11.184991	H	20.120603	25.218860	10.163234
C	21.151496	16.070014	9.583977	H	19.782556	23.668279	6.707848
C	21.280875	15.093628	10.764087	H	18.446383	23.691146	7.872373
C	21.473090	15.347789	8.268894	H	19.434944	25.132416	7.643919
C	17.433787	20.803928	8.721880	H	18.986735	20.639916	10.181846
C	25.914096	21.576061	7.562109	H	16.101020	20.002444	11.008648
C	19.469786	24.037099	7.690798	H	17.494036	19.916751	12.092352
C	24.355895	18.323125	9.124794	H	17.015111	21.469532	11.382215
C	24.461654	17.659471	7.889467	H	17.238289	21.868746	8.897286
C	25.784811	17.643759	7.139781	H	18.113919	20.717731	7.868292
C	26.817826	16.759833	7.851389	H	16.482406	20.334350	8.442927
C	28.164349	16.690542	7.129817	H	16.204224	18.242984	9.522976
C	29.190226	15.830292	7.865516	H	16.514790	15.817749	9.151592
H	23.617042	18.262148	6.773990	H	18.795367	14.860615	9.179764
H	24.259798	22.444972	11.584941	H	21.897614	16.857769	9.733591
H	22.940302	23.556119	11.239811	H	20.572037	14.262099	10.669817
H	22.847106	22.477178	12.649377	H	22.291447	14.668540	10.801039
H	22.205272	20.445085	12.538873	H	21.088293	15.587813	11.721938
H	21.160620	18.683073	13.203902	H	21.433873	16.026589	7.409232
H	19.562263	19.203039	12.666440	H	22.473342	14.899993	8.305836
H	20.181113	17.638329	12.146333	H	20.761942	14.539076	8.068842
H	24.932933	21.357835	9.451079	H	21.134647	15.143808	1.448660
H	26.120123	23.289302	10.586907	H	21.415272	16.188205	0.037080
H	27.216175	22.035085	9.973962	H	19.966493	16.396618	1.023301
H	27.038683	23.560785	9.101526	H	21.810102	18.369066	0.213538
H	26.315109	22.354783	6.903213	H	23.110489	19.879155	-0.557414

H	24.421885	20.611085	0.393736
H	22.789965	21.253128	0.510637
H	19.491096	17.118312	3.388841
H	18.368565	15.186226	2.207076
H	17.222425	16.325443	2.942494
H	17.547377	14.771121	3.716110
H	18.226262	15.941892	5.923294
H	17.774608	17.467660	5.147735
H	19.373459	17.278553	5.886877
H	19.084650	13.811283	5.062370
H	20.923685	12.204837	5.439693
H	23.223764	12.793388	4.761911
H	24.227092	16.139138	3.520985
H	25.387864	14.775742	1.792482
H	23.671743	14.990560	1.409306
H	24.242433	13.448913	2.072139
H	25.009562	14.788779	5.537369
H	26.184233	14.890039	4.212123
H	25.265923	13.407513	4.458621
H	25.383907	18.029701	2.537482
H	27.618999	19.048210	0.710059
H	25.920352	18.840839	0.254183
H	26.904248	17.425554	0.659127
H	27.485052	16.893883	3.057698
H	27.152790	18.067382	4.347664
H	28.375654	18.413704	3.117733
H	28.098082	20.564743	2.884730
H	27.697872	22.940994	3.430425
H	25.381347	23.791734	3.577176
H	22.358064	21.718978	2.872757
H	23.498726	24.547682	2.580730
H	21.847543	24.021295	2.239964
H	23.191837	23.445734	1.232551
H	22.729194	22.045918	5.323796
H	21.654052	23.289308	4.668329
H	23.344935	23.670714	5.024881
H	23.816880	17.866116	9.952294
H	25.099937	19.070571	9.396781
H	23.944337	16.698704	7.832975
H	25.632085	17.280934	6.113215
H	26.175127	18.665770	7.058184
H	26.963791	17.136339	8.871769
H	26.411377	15.742832	7.960004
H	28.562297	17.708120	7.005261
H	28.017379	16.295517	6.114922
H	29.388517	16.223242	8.870346
H	30.144528	15.794716	7.327293
H	28.833307	14.799160	7.979528
H	20.114927	19.446469	6.769453
H	20.906667	20.991700	6.540091
H	20.109743	19.093225	4.262191
H	20.893299	20.656742	4.059898
H	18.153794	20.191684	5.304585
H	18.923446	21.758969	5.115551
H	18.971874	21.441038	2.620850
H	18.216042	19.865860	2.803061
H	16.954026	22.527074	3.649586
H	16.198665	20.952353	3.829814
H	16.990501	22.228940	1.140791
H	15.344165	22.108309	1.780876
H	16.226896	20.643328	1.323571

184

[**E**]

C	2.057307	2.899381	-3.099994
C	2.366577	1.531067	-3.301168
C	3.724322	1.114805	-3.335124
C	4.729993	2.067207	-3.149718
C	4.430045	3.407028	-2.929257

C	3.101737	3.810179	-2.906809
N	1.340456	0.544388	-3.414740
C	0.773819	0.314301	-4.596754
C	1.048998	1.241322	-5.770690
C	4.099183	-0.329898	-3.631561
C	4.321738	-0.544733	-5.137694
C	0.628183	3.420266	-3.097722
C	0.425609	4.572444	-4.093916
C	-0.111269	-0.745667	-4.897460
C	-0.457510	-1.938853	-4.223261
C	-1.232426	-2.925991	-5.082943
N	-0.174067	-2.226308	-2.956120
C	-0.390450	-3.544151	-2.452166
C	-1.650826	-3.945161	-1.944319
C	-1.778516	-5.215892	-1.371914
C	-0.705352	-6.093702	-1.306165
C	0.524041	-5.704919	-1.827448
C	0.707866	-4.444322	-2.401588
C	-2.880848	-3.053162	-2.016136
C	-4.047841	-3.735524	-2.746938
C	2.050537	-4.081632	-3.019815
C	2.103219	-4.493948	-4.500304
C	-3.329830	-2.593421	-0.623333
C	3.251988	-4.677959	-2.277116
C	5.326640	-0.814972	-2.850705
C	0.189049	3.853310	-1.693440
Ca	0.340219	-0.421394	-1.450484
C	-1.679004	0.868821	-0.359682
C	-2.261136	0.947308	-1.779619
C	-3.661495	1.566959	-1.901111
C	-4.196999	1.624476	-3.332537
C	-5.592185	2.242773	-3.441742
C	-6.118160	2.295368	-4.874752
Ca	-0.327987	0.589443	1.729002

N	0.340871	2.379699	3.158724
C	0.714436	3.630809	2.579636
C	2.039129	3.876371	2.138572
C	2.339066	5.111763	1.554317
C	1.373749	6.098456	1.399434
C	0.072732	5.846553	1.819399
C	-0.282134	4.626561	2.401671
C	3.152154	2.846613	2.265823
C	4.345534	3.365272	3.083926
C	-1.717242	4.418012	2.865510
C	-2.023830	5.241627	4.126309
C	0.472123	2.215698	4.474240
C	1.211694	3.259755	5.293000
C	-0.024197	1.128642	5.225487
C	-0.939404	0.094090	4.925722
C	-1.492413	-0.610255	6.152956
N	-1.367315	-0.238323	3.712224
C	-2.421182	-1.193044	3.582966
C	-3.769422	-0.753235	3.568997
C	-4.786625	-1.693690	3.381944
C	-4.506064	-3.045556	3.223160
C	-3.183617	-3.470884	3.243155
C	-2.127109	-2.570261	3.414897
C	-4.147969	0.707242	3.770392
C	-5.020871	0.902886	5.019729
C	-0.704343	-3.111470	3.439919
C	-0.455331	-4.025863	4.650209
C	-0.357754	-3.857497	2.144235
C	-4.852596	1.288014	2.536004
C	3.640153	2.386445	0.884442
C	-2.739626	4.729931	1.764156
C	1.568191	-0.763336	0.839804
C	2.602309	-1.534147	0.007350
C	3.894303	-1.967231	0.727424

C	3.688529	-2.969973	1.861496	H	-5.788598	0.757224	2.323411
C	4.991624	-3.378297	2.551518	H	-5.822028	-1.360064	3.371270
C	4.784938	-4.390650	3.676389	H	-5.312522	-3.762377	3.088893
H	2.928360	-0.928915	-0.860076	H	-2.962489	-4.529509	3.126611
H	2.293444	3.151687	5.153275	H	-0.024120	-2.256872	3.532770
H	0.952288	4.274686	4.980129	H	-1.096775	-4.914859	4.614443
H	0.999460	3.154892	6.359385	H	0.586600	-4.368086	4.660579
H	0.232900	1.179594	6.278327	H	-0.649810	-3.513109	5.597657
H	-0.752811	-0.622939	6.958374	H	-0.509873	-3.231717	1.258668
H	-2.371905	-0.070751	6.525602	H	0.689826	-4.178428	2.154316
H	-1.808182	-1.633376	5.942526	H	-0.976660	-4.752694	2.017626
H	2.741734	1.975503	2.790520	H	-1.131103	-3.952425	-4.724809
H	4.042542	3.734233	4.069176	H	-0.907034	-2.880755	-6.125877
H	5.081382	2.565600	3.232161	H	-2.300169	-2.673167	-5.062611
H	4.854972	4.188170	2.568194	H	-0.498634	-0.703243	-5.910944
H	4.092790	3.213228	0.326101	H	0.942151	0.715747	-6.723064
H	4.395542	1.597552	0.982888	H	2.047498	1.681314	-5.719614
H	2.821655	1.995559	0.271530	H	0.329321	2.069629	-5.768500
H	3.355486	5.304337	1.218630	H	-2.610757	-2.161281	-2.591140
H	1.632061	7.055983	0.953562	H	-3.750649	-4.099364	-3.736089
H	-0.684360	6.617833	1.698109	H	-4.879147	-3.031793	-2.876355
H	-1.824453	3.360572	3.131685	H	-4.429762	-4.592352	-2.179571
H	-3.050811	5.059203	4.466173	H	-3.625266	-3.443038	0.002062
H	-1.347537	4.987061	4.948934	H	-4.188897	-1.915772	-0.694800
H	-1.921381	6.316034	3.929086	H	-2.530628	-2.064956	-0.092992
H	-2.550707	4.140284	0.860409	H	-2.744198	-5.523079	-0.976674
H	-3.753963	4.500182	2.110805	H	-0.825891	-7.078196	-0.860624
H	-2.724811	5.788976	1.480556	H	1.358246	-6.400096	-1.789697
H	-3.221684	1.271652	3.922641	H	2.137513	-2.989186	-2.984337
H	-5.993354	0.407464	4.911050	H	3.070920	-4.224068	-4.941028
H	-4.541869	0.494766	5.915966	H	1.320570	-4.004003	-5.086099
H	-5.210371	1.969498	5.191509	H	1.974604	-5.578726	-4.603573
H	-5.101090	2.344052	2.696487	H	3.210435	-4.477358	-1.201391
H	-4.223356	1.220926	1.642025	H	4.182410	-4.249162	-2.666876

H	3.320559	-5.764087	-2.414048
H	3.244367	-0.951005	-3.339252
H	5.137447	0.091565	-5.503539
H	3.424493	-0.309634	-5.717556
H	4.589450	-1.588738	-5.342723
H	5.448157	-1.896935	-2.978188
H	5.237697	-0.606723	-1.779296
H	6.251984	-0.346737	-3.207689
H	5.770513	1.755651	-3.181896
H	5.227838	4.131541	-2.784274
H	2.865367	4.858880	-2.742239
H	-0.023348	2.598462	-3.412699
H	0.968864	5.473296	-3.785410
H	-0.636726	4.838048	-4.155985
H	0.771054	4.305881	-5.098496
H	0.256523	3.026313	-0.978330
H	-0.850401	4.203217	-1.701280
H	0.815096	4.666215	-1.310246
H	1.224696	-1.455193	1.635524
H	2.136192	0.018064	1.388286
H	2.137022	-2.439803	-0.419636
H	4.589354	-2.400713	-0.007133
H	4.387030	-1.067484	1.125340
H	3.002837	-2.546907	2.608536
H	3.193073	-3.869565	1.464363
H	5.484180	-2.480747	2.952717
H	5.682967	-3.795445	1.805083
H	4.127037	-3.986985	4.456156
H	5.734289	-4.663982	4.151426
H	4.323860	-5.312359	3.299880
H	-2.436395	0.334775	0.255202
H	-1.697719	1.910134	0.031522
H	-2.311818	-0.058622	-2.232390
H	-1.586719	1.524308	-2.433528
H	-4.356264	0.993163	-1.270205
H	-3.634464	2.581634	-1.476960
H	-3.498171	2.197421	-3.960964
H	-4.217901	0.608434	-3.755305
H	-5.571326	3.258464	-3.021266
H	-6.291321	1.670154	-2.815392
H	-5.458579	2.891964	-5.517295
H	-7.118612	2.741050	-4.920203
H	-6.181227	1.290366	-5.310466

184

[**F**]

Ca	19.228654	12.998280	8.885079
N	17.229352	14.232036	9.294274
N	17.754398	11.114680	8.996304
C	15.186891	14.608790	10.615289
H	14.759721	15.263017	9.849170
H	14.378823	14.036123	11.075575
H	15.620681	15.264657	11.378782
C	16.251820	13.701097	10.025687
C	16.086036	12.326908	10.307898
H	15.248645	12.121151	10.965524
C	16.671975	11.157366	9.768427
C	15.956422	9.869044	10.138741
H	16.496832	9.353269	10.941359
H	14.940886	10.064384	10.490856
H	15.909270	9.177964	9.292810
C	17.137210	15.600498	8.895073
C	17.677716	16.636757	9.696570
C	18.302270	16.367410	11.057087
H	18.094547	15.322086	11.312944
C	17.696500	17.238884	12.167924
H	16.604301	17.159657	12.198209
H	18.087746	16.933269	13.145825
H	17.946841	18.297708	12.033002

C	19.825376	16.552594	11.016305
H	20.093899	17.583143	10.759419
H	20.273878	16.321120	11.990058
H	20.295276	15.902111	10.269772
C	17.639188	17.952522	9.223713
H	18.060234	18.747081	9.835294
C	17.068563	18.267022	7.996795
H	17.040946	19.297343	7.650414
C	16.525353	17.249467	7.221676
H	16.065860	17.495246	6.266822
C	16.549022	15.916806	7.642784
C	15.906603	14.856694	6.759009
H	16.108221	13.881156	7.213857
C	14.380766	15.030919	6.693963
H	14.109948	15.992115	6.239658
H	13.928623	14.235946	6.088184
H	13.925357	14.993647	7.689215
C	16.499430	14.844717	5.343655
H	17.579604	14.666374	5.362726
H	16.036034	14.052728	4.743384
H	16.325433	15.793291	4.821429
C	18.113753	9.886071	8.362366
C	17.718539	9.673111	7.015144
C	16.856211	10.682594	6.270892
H	16.967765	11.645566	6.782883
C	15.368584	10.302450	6.342013
H	15.012800	10.260184	7.376063
H	14.755692	11.037870	5.806206
H	15.194699	9.320322	5.884594
C	17.283411	10.875459	4.810651
H	17.086619	9.984258	4.202704
H	16.721753	11.701405	4.360316
H	18.350489	11.108872	4.730392
C	18.095273	8.487972	6.377467

H	17.781604	8.312919	5.351816
C	18.860059	7.525344	7.026459
H	19.142183	6.609427	6.512795
C	19.259500	7.747706	8.337595
H	19.863515	6.998960	8.844806
C	18.897784	8.910316	9.026865
C	19.379680	9.082860	10.459498
H	18.890676	9.973557	10.869502
C	19.005124	7.894691	11.358729
H	19.540592	6.983979	11.065729
H	19.271747	8.108465	12.400812
H	17.933326	7.672886	11.319541
C	20.896705	9.314395	10.504124
H	21.194208	10.177058	9.898993
H	21.235040	9.490725	11.532356
H	21.441246	8.446263	10.115926
C	21.364164	13.134431	10.369903
H	21.935147	14.039546	10.666760
H	22.105238	12.311659	10.439122
C	20.339937	12.893312	11.491500
H	19.597479	13.710980	11.514188
H	19.756825	11.973959	11.295398
C	20.903956	12.762011	12.914096
H	21.483988	13.667326	13.144783
H	21.621564	11.929025	12.931742
C	19.837401	12.547316	13.989185
H	19.253712	11.645209	13.750337
H	19.122222	13.383683	13.964526
C	20.407528	12.416644	15.402794
H	21.116995	11.577135	15.430643
H	20.993287	13.316156	15.640980
C	19.332594	12.211549	16.468431
H	18.752868	11.300207	16.275867
H	19.770191	12.122300	17.469480

H	18.628121	13.052414	16.486557
Ca	22.472553	13.625481	8.153301
N	24.471996	12.391944	7.744113
N	23.946614	15.509236	8.042066
C	26.514520	12.015386	6.423119
H	26.941313	11.360699	7.189044
H	27.322848	12.588141	5.963389
H	26.080866	11.360022	5.659102
C	25.449521	12.922973	7.012760
C	25.615209	14.297184	6.730607
H	26.452630	14.503040	6.073048
C	25.029116	15.466659	7.270049
C	25.744604	16.755049	6.899847
H	25.204160	17.270905	6.097306
H	26.760141	16.559785	6.547689
H	25.791757	17.446030	7.745858
C	24.564218	11.023500	8.143358
C	24.023841	9.987158	7.341880
C	23.399431	10.256352	5.981263
H	23.607004	11.301708	5.725414
C	24.005560	9.384960	4.870555
H	25.097746	9.464410	4.840448
H	23.614408	9.690452	3.892578
H	23.755422	8.326088	5.005487
C	21.876351	10.070878	6.021775
H	21.607990	9.040286	6.278656
H	21.427993	10.302212	5.047922
H	21.406172	10.721297	6.768188
C	24.062423	8.671424	7.814824
H	23.641491	7.876797	7.203251
C	24.632980	8.357035	9.041801
H	24.660639	7.326737	9.388246
C	25.176086	9.374670	9.816890
H	25.635535	9.128979	10.771787

C	25.152361	10.707303	9.395697
C	25.794688	11.767514	10.279420
H	25.592989	12.743011	9.824520
C	27.320540	11.593421	10.344454
H	27.591445	10.632247	10.798754
H	27.772619	12.388434	10.950228
H	27.775926	11.630731	9.349193
C	25.201867	11.779515	11.694776
H	24.121673	11.957738	11.675699
H	25.665183	12.571593	12.294993
H	25.375973	10.830993	12.217060
C	23.587048	16.737823	8.675920
C	23.981957	16.950803	10.023230
C	24.844093	15.941300	10.767676
H	24.732349	14.978254	10.255858
C	26.331798	16.321112	10.696469
H	26.687599	16.363043	9.662413
H	26.944530	15.585695	11.232466
H	26.505875	17.303316	11.153653
C	24.416880	15.748782	12.227958
H	24.614016	16.639987	12.835791
H	24.978259	14.922692	12.678375
H	23.349720	15.515764	12.308293
C	23.605075	18.135950	10.660807
H	23.918508	18.311020	11.686525
C	22.840408	19.098552	10.011633
H	22.558168	20.014479	10.525216
C	22.441216	18.876142	8.700428
H	21.837265	19.624851	8.193089
C	22.803096	17.713528	8.011254
C	22.321420	17.540909	6.578554
H	22.810565	16.650253	6.168629
C	22.695961	18.729105	5.679352
H	22.160336	19.639755	5.972255

H	22.429514	18.515276	4.637235
H	23.767725	18.951049	5.718685
C	20.804423	17.309223	6.533734
H	20.506925	16.446532	7.138829
H	20.466237	17.132860	5.505458
H	20.259748	18.177301	6.921865
C	20.337083	13.489115	6.668462
H	19.766183	12.583926	6.371686
H	19.595944	14.311818	6.599141
C	21.361320	13.730200	5.546870
H	22.103832	12.912570	5.524287
H	21.944366	14.649612	5.742898
C	20.797339	13.861322	4.124244
H	20.217327	12.955970	3.893648
H	20.079723	14.694299	4.106475
C	21.863922	14.075898	3.049159
H	22.447622	14.978018	3.287930
H	22.579084	13.239519	3.073913
C	21.293831	14.206439	1.635523
H	20.584405	15.045979	1.607564
H	20.708034	13.306928	1.397426
C	22.368798	14.411361	0.569887
H	22.948566	15.322695	0.762361
H	21.931227	14.500521	-0.431181
H	23.073230	13.570459	0.551869

186

[**F**1] scf done: -4306.903730 / Energies= -4305.158315 / Enthalpies= -4305.157371 / Free Energies= -4305.393277

C	1.973309	4.359591	-0.301677
C	2.816718	3.364311	0.251387
C	3.316387	3.520670	1.571356
C	2.951688	4.652992	2.305206
C	2.111411	5.623947	1.772683
C	1.631389	5.469485	0.478166
N	3.140035	2.176474	-0.472624
C	4.220638	2.174509	-1.254078
C	4.919956	3.486082	-1.568117
C	4.282986	2.519201	2.189281
C	3.822488	2.027109	3.567679
C	1.432359	4.268234	-1.720223
C	-0.081687	4.020198	-1.727175
C	4.813388	1.037205	-1.836889
C	4.624911	-0.352166	-1.643389
C	5.746547	-1.196620	-2.229248
N	3.600806	-0.916803	-1.017070
C	3.582319	-2.321955	-0.774281
C	3.206922	-3.249858	-1.778880
C	3.102305	-4.603648	-1.441136
C	3.360370	-5.056073	-0.154414
C	3.744273	-4.144340	0.823143
C	3.864261	-2.781300	0.540334
C	2.917314	-2.842345	-3.218052
C	1.456753	-3.112641	-3.609489
C	4.359599	-1.823788	1.615144
C	3.938922	-2.213232	3.036395
C	3.852634	-3.550334	-4.212303
C	5.888220	-1.672572	1.543954
C	5.702528	3.101556	2.283257
C	1.763498	5.513685	-2.556923
Ca	1.621363	0.330096	-0.397660
C	0.499610	0.038989	1.864053
C	-0.528579	-0.167934	2.990237
C	0.017892	-0.157606	4.425411
C	-1.050857	-0.359597	5.500776
C	-0.497183	-0.344910	6.926811
C	-1.573570	-0.539759	7.992851
C	-0.536319	0.213301	-1.842960

C	0.482674	0.522159	-2.954145
C	-0.080133	0.663240	-4.376100
C	0.980954	0.940614	-5.442231
C	0.408325	1.093281	-6.852602
C	1.478009	1.351293	-7.911978
Ca	-1.631901	-0.238102	0.406520
N	-3.654897	0.986277	0.777407
C	-3.741207	2.333255	0.310709
C	-3.242329	3.413754	1.080267
C	-3.277390	4.704115	0.540991
C	-3.805104	4.950838	-0.720142
C	-4.311411	3.890535	-1.463017
C	-4.290397	2.580973	-0.975200
C	-2.680572	3.222613	2.480833
C	-1.167310	3.475317	2.521210
C	-4.909504	1.470379	-1.813150
C	-4.338438	1.415354	-3.236150
C	-4.656425	0.486820	1.501064
C	-4.833518	-0.870585	1.837572
C	-4.214139	-2.062018	1.391764
C	-4.967090	-3.321461	1.791966
C	-5.736757	1.416429	2.027086
N	-3.090499	-2.137379	0.688674
C	-2.644849	-3.391085	0.173975
C	-2.861092	-3.672037	-1.201234
C	-2.350240	-4.855883	-1.740149
C	-1.635463	-5.757991	-0.960101
C	-1.433307	-5.481024	0.385215
C	-1.927327	-4.313126	0.976286
C	-3.691527	-2.739921	-2.071835
C	-3.216308	-2.668323	-3.527262
C	-1.669630	-4.091107	2.460569
C	-0.172204	-3.922251	2.757371
C	-3.385293	4.108881	3.519835

C	-6.440641	1.600471	-1.862087
C	-5.178466	-3.127842	-2.022501
C	-2.242026	-5.228872	3.321459
H	-6.046812	2.139894	1.268303
H	-6.615056	0.860949	2.363769
H	-5.356884	1.994617	2.877931
H	-5.705685	-1.052163	2.457788
H	-4.732438	-3.580833	2.831692
H	-6.047572	-3.161353	1.734602
H	-4.703308	-4.177230	1.167518
H	-2.858327	2.179228	2.765166
H	-4.473006	3.983543	3.493579
H	-3.036833	3.860748	4.529806
H	-3.172200	5.171333	3.352846
H	-0.930403	4.510834	2.253310
H	-0.768233	3.290654	3.525938
H	-0.623872	2.831370	1.821450
H	-2.891149	5.534019	1.128492
H	-3.830240	5.962474	-1.118007
H	-4.741457	4.084500	-2.443113
H	-4.676556	0.519936	-1.321299
H	-6.741871	2.540414	-2.341201
H	-6.878651	0.775536	-2.437575
H	-6.881400	1.581238	-0.859976
H	-3.251815	1.282036	-3.228301
H	-4.778519	0.576560	-3.789001
H	-4.560560	2.328779	-3.801013
H	-3.610317	-1.733318	-1.642884
H	-5.570291	-3.094554	-1.001534
H	-5.777265	-2.443441	-2.636163
H	-5.325056	-4.144787	-2.407604
H	-3.414876	-3.599838	-4.071078
H	-3.749113	-1.869537	-4.055197
H	-2.143056	-2.460882	-3.596158

H	-2.516553	-5.079471	-2.790299
H	-1.243345	-6.672543	-1.398177
H	-0.878207	-6.189102	0.996388
H	-2.175198	-3.164679	2.754816
H	-1.710344	-6.170888	3.141415
H	-2.136153	-4.991402	4.387127
H	-3.302366	-5.404215	3.114391
H	0.262499	-3.088585	2.197072
H	-0.010463	-3.732742	3.825656
H	0.389118	-4.824791	2.488607
H	1.016615	0.993692	2.091141
H	1.281604	-0.735489	2.012641
H	-1.311365	0.610677	2.942660
H	-1.063977	-1.127054	2.858761
H	0.535256	0.797896	4.594157
H	0.785922	-0.939394	4.514184
H	-1.570729	-1.313803	5.325112
H	-1.818320	0.423445	5.403471
H	0.265063	-1.130971	7.027160
H	0.024678	0.606872	7.101996
H	-2.091099	-1.498399	7.862582
H	-1.146967	-0.526528	9.002507
H	-2.330323	0.252883	7.940677
H	5.761541	-2.209495	-1.822495
H	6.717308	-0.727707	-2.042801
H	5.627918	-1.271205	-3.317124
H	5.679744	1.265398	-2.449859
H	4.327272	4.067525	-2.284379
H	5.907463	3.320252	-2.004689
H	5.029764	4.107290	-0.674937
H	3.092909	-1.764294	-3.304405
H	4.906889	-3.411138	-3.954213
H	3.694614	-3.163275	-5.226447
H	3.659041	-4.629363	-4.239438
H	1.222304	-4.182074	-3.552202
H	1.269534	-2.784586	-4.639313
H	0.754061	-2.589982	-2.954299
H	2.809759	-5.318160	-2.207105
H	3.270572	-6.112739	0.085282
H	3.959609	-4.502431	1.825864
H	3.933197	-0.835928	1.398982
H	6.378885	-2.638758	1.715589
H	6.243544	-0.970881	2.308707
H	6.211393	-1.298440	0.568192
H	2.862119	-2.400650	3.107283
H	4.187341	-1.405124	3.733309
H	4.462074	-3.111212	3.386900
H	4.327798	1.652568	1.521092
H	6.084436	3.397841	1.300977
H	6.395114	2.361628	2.703230
H	5.724234	3.986855	2.931014
H	3.793379	2.840543	4.302730
H	4.516474	1.268814	3.950411
H	2.824258	1.579903	3.522362
H	3.341530	4.781131	3.312606
H	1.839784	6.498004	2.359704
H	0.981323	6.233020	0.056502
H	1.917351	3.411393	-2.201495
H	1.237817	6.400783	-2.184249
H	1.454403	5.364734	-3.598651
H	2.835532	5.738291	-2.548297
H	-0.350166	3.119399	-1.164780
H	-0.453415	3.898842	-2.751778
H	-0.624446	4.855972	-1.272040
H	-1.070170	-0.707485	-2.159111
H	-1.307719	1.008202	-1.903763
H	1.262902	-0.260498	-2.993624
H	1.023830	1.460995	-2.732710

H -0.626615 -0.256703 -4.629061
H -0.826498 1.470739 -4.377562
H 1.536922 1.853015 -5.177456
H 1.721087 0.126037 -5.439137
H -0.321273 1.915761 -6.859538
H -0.155507 0.186239 -7.114184
H 2.037130 2.270057 -7.695118
H 1.038273 1.457248 -8.910469
H 2.201381 0.527399 -7.952343
H 0.587201 -2.818115 -0.209663
H 0.233450 -2.362357 -0.679232

186

[TS-**F1F2**] scf done: -4306.884899 / Energies= -4305.139672 / Enthalpies= -4305.138728 / Free Energies= -4305.371088

C 23.070572 17.592384 8.415191
C 23.836882 16.508846 8.912464
C 24.335684 16.556967 10.241534
C 24.053824 17.673375 11.033873
C 23.289950 18.730960 10.554573
C 22.806145 18.680480 9.254087
N 24.081893 15.339666 8.126991
C 25.162719 15.305030 7.343801
C 25.942132 16.582503 7.082837
C 25.210223 15.449262 10.812485
C 24.740304 14.978626 12.194670
C 22.523650 17.627264 6.996201
C 20.989557 17.581168 6.988714
C 25.681865 14.160883 6.708289
C 25.442211 12.774483 6.876048
C 26.541387 11.910449 6.276090
N 24.410080 12.234050 7.509006
C 24.373435 10.821674 7.732065
C 23.816798 9.944458 6.766387
C 23.745312 8.578975 7.057734
C 24.207880 8.065320 8.262836
C 24.760172 8.929019 9.200402
C 24.852840 10.303529 8.960938
C 23.328565 10.422391 5.406390
C 21.861279 10.046649 5.154314
C 25.520377 11.189290 10.002687
C 24.911585 11.017773 11.400506
C 24.206503 9.885295 4.263963
C 27.036184 10.938318 10.047520
C 26.684544 15.880308 10.869961
C 23.005571 18.858867 6.213160
Ca 22.499614 13.549945 8.133936
C 21.259358 13.598375 10.326814
C 20.246289 13.353828 11.458574
C 20.760265 13.564631 12.890265
C 19.707747 13.327854 13.974534
C 20.238724 13.525030 15.395710
C 19.178431 13.298864 16.471625
C 20.176702 13.798214 6.575785
C 21.252852 14.001879 5.504080
C 20.746485 14.496164 4.141948
C 21.852884 14.688329 3.103901
C 21.338105 15.169618 1.746002
C 22.448935 15.360180 0.715289
Ca 19.179123 12.929360 8.921306
N 17.113641 14.121117 9.181740
C 17.009362 15.437729 8.638692
C 17.391870 16.583338 9.379971
C 17.344144 17.837421 8.760317
C 16.924482 17.984099 7.445313
C 16.538803 16.858538 6.725760
C 16.573085 15.582801 7.294178

C	17.843057	16.513284	10.831738	H	17.089056	18.445235	11.532706
C	19.315864	16.918136	10.984432	H	19.471548	17.962328	10.689616
C	16.084164	14.385353	6.492298	H	19.640934	16.815560	12.026813
C	16.442317	14.456938	5.003570	H	19.975413	16.303321	10.364090
C	16.135186	13.630549	9.936099	H	17.638647	18.718002	9.326738
C	16.025595	12.292014	10.378858	H	16.891801	18.968353	6.984498
C	16.686690	11.101814	10.004906	H	16.199895	16.976316	5.700154
C	16.024020	9.823698	10.486111	H	16.566328	13.494498	6.912812
C	15.001020	14.527313	10.407159	H	14.026295	15.076999	6.288717
N	17.791988	11.024207	9.265599	H	14.221786	13.323533	6.092872
C	18.214545	9.740037	8.799864	H	14.291077	14.048426	7.707257
C	17.806366	9.302690	7.514600	H	17.509946	14.647639	4.852808
C	18.258869	8.067226	7.041888	H	16.192208	13.509408	4.512750
C	19.104940	7.266656	7.799018	H	15.881036	15.242075	4.482653
C	19.507752	7.703683	9.054722	H	16.702502	11.081269	7.150063
C	19.073973	8.925280	9.577802	H	15.017541	9.270254	7.422981
C	16.874840	10.128052	6.638752	H	14.842728	10.072294	5.853132
C	17.499059	10.432241	5.269618	H	15.610248	8.478461	5.955630
C	19.561448	9.345341	10.955835	H	17.659323	9.517014	4.687048
C	21.049507	9.721138	10.914689	H	16.840280	11.083176	4.682427
C	16.966140	17.377837	11.751677	H	18.469517	10.929884	5.371187
C	14.567335	14.197263	6.659242	H	17.939063	7.725090	6.059919
C	15.508981	9.447155	6.460429	H	19.448352	6.309751	7.413498
C	19.318057	8.274709	12.029928	H	20.175119	7.080751	9.645587
H	14.851255	15.383099	9.745645	H	18.995061	10.236811	11.249308
H	14.063962	13.968971	10.482286	H	19.921989	7.377442	11.850697
H	15.229851	14.918215	11.406543	H	19.593469	8.661042	13.018794
H	15.172504	12.127749	11.028923	H	18.268287	7.963292	12.068157
H	16.698709	9.252580	11.132243	H	21.248066	10.499826	10.169743
H	15.106128	10.030374	11.040694	H	21.387026	10.083892	11.893622
H	15.780590	9.171699	9.640844	H	21.666425	8.856843	10.643311
H	17.741853	15.473511	11.162177	H	21.646387	14.627254	10.462471
H	15.903098	17.139351	11.644531	H	22.128354	12.945291	10.563777
H	17.247387	17.224418	12.800715	H	19.365263	14.010460	11.336130

H	19.853422	12.320815	11.411372
H	21.151977	14.588526	12.972627
H	21.617381	12.895478	13.054693
H	19.304567	12.308290	13.877556
H	18.856202	14.005636	13.810490
H	21.083557	12.841845	15.563962
H	20.647397	14.541310	15.491145
H	18.774855	12.279885	16.421926
H	19.588521	13.445664	17.477513
H	18.337169	13.992646	16.350510
H	26.354040	10.843085	6.395685
H	27.502297	12.147740	6.746338
H	26.646718	12.131855	5.207777
H	26.549031	14.365388	6.087430
H	25.402973	17.214177	6.367262
H	26.929591	16.371171	6.666252
H	26.063953	17.172558	7.995302
H	23.405880	11.515533	5.393360
H	25.257650	10.162513	4.388485
H	23.863334	10.280987	3.299863
H	24.155344	8.791032	4.210384
H	21.735143	8.960836	5.069206
H	21.509325	10.491029	4.214619
H	21.215018	10.390902	5.966928
H	23.321124	7.902949	6.319193
H	24.145826	6.998955	8.465844
H	25.138837	8.527067	10.137330
H	25.369011	12.230095	9.696360
H	27.254429	9.910827	10.364264
H	27.520967	11.618909	10.758359
H	27.497477	11.087577	9.065863
H	23.835880	11.223272	11.399151
H	25.387038	11.704427	12.110459
H	25.054868	10.000943	11.784676
H	25.144723	14.596145	10.128300
H	27.066354	16.150621	9.880180
H	27.309145	15.067373	11.260563
H	26.813651	16.749070	11.527580
H	24.827193	15.769680	12.949098
H	25.355136	14.137975	12.537075
H	23.696679	14.649267	12.170832
H	24.445603	17.717406	12.047255
H	23.079530	19.589404	11.187824
H	22.214708	19.509935	8.872731
H	22.895040	16.736245	6.477065
H	22.597005	19.784312	6.636240
H	22.673793	18.801250	5.169215
H	24.096592	18.949655	6.219735
H	20.604159	16.720289	7.543598
H	20.602781	17.526894	5.964226
H	20.565660	18.477409	7.456076
H	19.337509	13.255141	6.105200
H	19.764167	14.775036	6.877139
H	21.795728	13.055269	5.333738
H	22.012299	14.730899	5.843499
H	20.007649	13.774943	3.763757
H	20.205382	15.441867	4.286695
H	22.592161	15.408480	3.486062
H	22.395942	13.740683	2.969541
H	20.795001	16.116215	1.880004
H	20.601446	14.449098	1.362641
H	23.183390	16.100280	1.056552
H	22.050250	15.705503	-0.245516
H	22.986624	14.421185	0.534513
H	20.984440	11.779701	7.800610
H	20.668576	12.567174	7.349127

186

[**F2**] scf done: -4306.954184 / Energies= -4305.203734 / Enthalpies= -4305.202790 / Free Energies= -4305.447600

C	0.889270	4.255377	-0.294140
C	1.928388	3.448980	0.233796
C	2.491137	3.754421	1.501426
C	1.996496	4.851897	2.211427
C	0.963953	5.635665	1.707989
C	0.420084	5.331988	0.465791
N	2.362229	2.278233	-0.452718
C	3.330188	2.355549	-1.360894
C	3.855681	3.712336	-1.792304
C	3.654215	2.954841	2.075510
C	3.421906	2.517610	3.527877
C	0.282869	3.999025	-1.667130
C	-1.168354	3.508705	-1.569240
C	3.931168	1.251784	-1.999662
C	3.795197	-0.136131	-1.790422
C	4.737435	-1.001972	-2.606506
N	2.918864	-0.704329	-0.967187
C	2.959865	-2.111282	-0.742810
C	2.239623	-2.999317	-1.580804
C	2.223252	-4.361353	-1.262913
C	2.913002	-4.859261	-0.164154
C	3.632024	-3.985195	0.643681
C	3.665477	-2.612364	0.382835
C	1.500328	-2.522519	-2.823704
C	-0.021811	-2.640754	-2.664453
C	4.498305	-1.702306	1.276416
C	4.203749	-1.898831	2.769574
C	1.957506	-3.270942	-4.085808
C	6.001081	-1.884651	1.008687
C	4.972576	3.738120	1.966239
C	0.361453	5.240332	-2.569719
Ca	1.068393	0.361244	0.069609
C	0.271997	-0.104870	2.436568
C	-0.535182	-0.461339	3.696499
C	0.235669	-0.455237	5.024802
C	-0.614810	-0.815898	6.243765
C	0.165808	-0.808044	7.559440
C	-0.691537	-1.167965	8.771162
C	1.926012	1.275696	-7.012878
C	3.415529	1.404902	-7.324739
C	3.737345	1.258271	-8.812672
C	5.227513	1.384577	-9.131588
C	5.550889	1.237740	-10.619249
C	7.041204	1.366774	-10.927222
Ca	-2.055427	-0.446782	1.304320
N	-4.046833	0.658192	1.999179
C	-4.202672	2.044055	1.685661
C	-3.713032	3.052939	2.553815
C	-3.834645	4.392932	2.172629
C	-4.429164	4.757026	0.970895
C	-4.906548	3.764611	0.123846
C	-4.803056	2.410020	0.453828
C	-3.048322	2.734742	3.885303
C	-1.554901	3.092258	3.854112
C	-5.353467	1.378045	-0.520025
C	-4.643041	1.452638	-1.879156
C	-4.990429	0.049007	2.712174
C	-5.078399	-1.341640	2.950951
C	-4.443143	-2.453733	2.354038
C	-5.080654	-3.795993	2.662414
C	-6.118348	0.871350	3.307387
N	-3.390159	-2.406441	1.541079
C	-3.005133	-3.593436	0.843775
C	-3.537493	-3.840214	-0.447489
C	-3.116924	-4.972559	-1.151076

C	-2.185948	-5.855196	-0.617191	H	-4.780630	2.433215	-2.350651
C	-1.663507	-5.606161	0.645660	H	-4.752215	-2.094250	-0.398387
C	-2.057796	-4.492840	1.394640	H	-6.313768	-4.050423	-0.414054
C	-4.562843	-2.919163	-1.093298	H	-6.637404	-2.936538	-1.753634
C	-4.029900	-2.317698	-2.401440	H	-5.787105	-4.460301	-2.052587
C	-1.446949	-4.289727	2.772875	H	-3.791038	-3.098726	-3.133297
C	0.047695	-3.953129	2.669396	H	-4.779719	-1.659197	-2.855304
C	-3.725952	3.441146	5.069965	H	-3.120401	-1.731238	-2.232203
C	-6.873065	1.517209	-0.698973	H	-3.531009	-5.166642	-2.138131
C	-5.900017	-3.634800	-1.339201	H	-1.872300	-6.731517	-1.179363
C	-1.646390	-5.503360	3.693753	H	-0.934116	-6.295288	1.065196
H	-6.684927	1.373273	2.515305	H	-1.957377	-3.438422	3.237973
H	-6.808023	0.250710	3.883338	H	-1.097982	-6.379785	3.328802
H	-5.731930	1.660986	3.958659	H	-1.274818	-5.280327	4.701282
H	-5.892139	-1.614628	3.613120	H	-2.701233	-5.786297	3.777527
H	-4.348483	-4.492690	3.082313	H	0.224715	-3.071997	2.043239
H	-5.909955	-3.695800	3.365934	H	0.472439	-3.756000	3.661388
H	-5.459518	-4.260952	1.745793	H	0.608645	-4.781282	2.221888
H	-3.137657	1.654945	4.051285	H	0.724426	0.890378	2.632061
H	-4.799599	3.229795	5.116937	H	1.137512	-0.800584	2.421845
H	-3.275750	3.113964	6.014974	H	-1.386591	0.233252	3.818967
H	-3.607152	4.529490	5.009772	H	-0.988298	-1.463936	3.592738
H	-1.411331	4.169990	3.716159	H	0.680664	0.540402	5.165918
H	-1.065429	2.805724	4.792982	H	1.079233	-1.155849	4.941684
H	-1.032183	2.592007	3.032354	H	-1.063136	-1.810269	6.095680
H	-3.457834	5.167848	2.836292	H	-1.459057	-0.113798	6.320864
H	-4.521131	5.805504	0.697836	H	1.008005	-1.510861	7.484132
H	-5.375309	4.046038	-0.816590	H	0.612893	0.185373	7.708527
H	-5.162210	0.386111	-0.096461	H	-1.122564	-2.171540	8.667498
H	-7.137282	2.483946	-1.144867	H	-0.105746	-1.151552	9.697501
H	-7.254855	0.730510	-1.361072	H	-1.523679	-0.463013	8.890912
H	-7.401481	1.437611	0.257261	H	5.074506	-1.876529	-2.044292
H	-3.566029	1.279321	-1.779041	H	5.610676	-0.435250	-2.938910
H	-5.045537	0.698432	-2.565054	H	4.222157	-1.376847	-3.499455

H	4.684692	1.518798	-2.733122
H	3.188251	4.149295	-2.545479
H	4.850847	3.631113	-2.236474
H	3.895610	4.416199	-0.956982
H	1.736608	-1.461818	-2.965901
H	3.045000	-3.232447	-4.212443
H	1.493547	-2.830552	-4.976564
H	1.666266	-4.327582	-4.053624
H	-0.324987	-3.676748	-2.474639
H	-0.528894	-2.301807	-3.576444
H	-0.393285	-2.027302	-1.835847
H	1.663186	-5.046190	-1.895213
H	2.895760	-5.923474	0.058276
H	4.182869	-4.377814	1.495278
H	4.244319	-0.667867	1.017769
H	6.317763	-2.910727	1.233975
H	6.589975	-1.205263	1.637328
H	6.254178	-1.679064	-0.036283
H	3.139451	-1.771131	2.993690
H	4.763856	-1.167621	3.364705
H	4.499688	-2.895896	3.117132
H	3.761860	2.049939	1.466492
H	5.203827	3.998949	0.928635
H	5.807394	3.143538	2.357566
H	4.924575	4.670492	2.542630
H	3.361172	3.375126	4.208543
H	4.252872	1.889250	3.870470
H	2.497639	1.940330	3.634121
H	2.432094	5.100617	3.176343
H	0.590566	6.482998	2.277992
H	-0.383918	5.948326	0.070627
H	0.867702	3.205499	-2.146359
H	-0.280488	6.048435	-2.199304
H	0.024171	4.993410	-3.583666
H	1.381911	5.633332	-2.634679
H	-1.240649	2.556904	-1.030890
H	-1.587385	3.350873	-2.570771
H	-1.802899	4.234599	-1.048109
H	1.538685	0.298222	-7.326433
H	1.343008	2.044195	-7.535643
H	3.975061	0.647278	-6.757465
H	3.780412	2.379556	-6.969735
H	3.370598	0.283281	-9.168060
H	3.177919	2.016750	-9.380931
H	5.594167	2.359549	-8.776015
H	5.786687	0.626075	-8.562976
H	4.992033	1.995275	-11.187296
H	5.186227	0.263187	-10.974533
H	7.427389	2.345098	-10.615094
H	7.240880	1.257365	-11.999424
H	7.622776	0.599853	-10.400691
H	-1.010239	0.056210	-0.552206
H	1.731123	1.382335	-5.939364

168

[**F3**] scf done: -4071.120272 / Energies= -4069.557019 / Enthalpies= -4069.556075 / Free Energies= -4069.774684

C	-3.768302	-0.717346	2.744794
C	-2.475630	-1.200899	3.072701
C	-2.191237	-2.584659	2.966008
C	-3.198320	-3.450393	2.528159
C	-4.467925	-2.983703	2.210062
C	-4.742431	-1.625436	2.321110
N	-1.456363	-0.273977	3.445932
C	-1.329377	0.045732	4.733342
C	-2.149792	-0.700738	5.768368
C	-0.825230	-3.156472	3.316882
C	-0.109196	-3.689546	2.068187

C	-4.124995	0.759862	2.841342
C	-4.425944	1.354614	1.457616
C	-0.510031	1.069593	5.242982
C	0.235977	2.081016	4.598861
C	0.659222	3.212609	5.521348
N	0.516618	2.125640	3.301508
C	1.208734	3.259025	2.782802
C	2.612822	3.192884	2.584590
C	3.261337	4.294734	2.017876
C	2.560477	5.434374	1.637555
C	1.185322	5.485335	1.825532
C	0.488797	4.416860	2.397957
C	3.419665	1.968991	3.002674
C	4.604295	1.679434	2.073126
C	-1.019364	4.526472	2.574106
C	-1.741609	4.508416	1.218602
C	3.924105	2.075589	4.451347
C	-1.423903	5.772512	3.375500
C	-0.905959	-4.257594	4.385136
C	-5.299229	1.012356	3.798788
Ca	-0.169463	0.539140	1.626557
Ca	0.174534	-0.523919	-1.680167
N	-0.544994	-2.287427	-3.165521
C	-1.225253	-3.362517	-2.518460
C	-2.616368	-3.267433	-2.264057
C	-3.247806	-4.302973	-1.568124
C	-2.546356	-5.424783	-1.145497
C	-1.185012	-5.520036	-1.413606
C	-0.499833	-4.504902	-2.088517
C	-3.451639	-2.091518	-2.752288
C	-3.988669	-1.249674	-1.587008
C	0.984109	-4.673687	-2.391130
C	1.764896	-5.317303	-1.237213
C	-0.507670	-2.258391	-4.492999
C	-1.184249	-3.345241	-5.314318
C	0.072602	-1.242676	-5.283070
C	0.548799	0.045145	-4.975216
C	0.887011	0.895687	-6.186719
N	0.661805	0.564398	-3.753404
C	1.035761	1.937046	-3.631105
C	2.396772	2.296830	-3.460105
C	2.723019	3.643060	-3.270883
C	1.745033	4.629784	-3.243350
C	0.413727	4.272713	-3.421116
C	0.033976	2.942514	-3.626101
C	3.517295	1.266494	-3.495929
C	4.315776	1.252683	-2.185553
C	-1.437821	2.627810	-3.862941
C	-2.293991	2.922617	-2.622808
C	-1.996216	3.384973	-5.078584
C	4.469048	1.485158	-4.682756
C	-4.607140	-2.551038	-3.655420
C	1.219294	-5.488078	-3.674388
C	1.725190	-0.679668	0.439123
C	2.531468	-1.643549	-0.445042
C	3.981041	-1.960662	-0.027903
C	4.112725	-2.696941	1.304438
C	5.560317	-3.034054	1.667347
C	5.691729	-3.764119	3.002484
H	2.607946	-1.260416	-1.485092
H	-1.147066	0.593413	-0.324906
H	1.519107	3.769116	5.144882
H	-0.169639	3.923558	5.628834
H	0.888604	2.832227	6.521194
H	-0.556501	1.179878	6.321283
H	-3.221018	-0.536664	5.607739
H	-1.988630	-1.780745	5.693542
H	-1.900748	-0.381007	6.782687

H	2.745364	1.103687	2.961986
H	3.102186	2.132132	5.169289
H	4.530885	1.198304	4.707949
H	4.549921	2.967649	4.579161
H	5.408274	2.415530	2.195168
H	5.030547	0.697475	2.307334
H	4.307711	1.678466	1.019449
H	4.336796	4.261369	1.867755
H	3.085631	6.276779	1.194197
H	0.638033	6.374899	1.521786
H	-1.354477	3.649836	3.138849
H	-2.504705	5.770946	3.561592
H	-0.911967	5.814274	4.342859
H	-1.185758	6.696347	2.834926
H	-1.521213	3.598908	0.648394
H	-2.828123	4.561497	1.357863
H	-1.440516	5.361614	0.598842
H	-3.252468	1.283923	3.246178
H	-6.223132	0.546087	3.435743
H	-5.099533	0.615730	4.800275
H	-5.488689	2.088513	3.894544
H	-4.637756	2.428096	1.537683
H	-3.583751	1.222280	0.769087
H	-5.301834	0.876322	1.002394
H	-5.736390	-1.259540	2.072818
H	-5.239214	-3.674772	1.878869
H	-2.983008	-4.512313	2.437051
H	-0.220532	-2.340618	3.729724
H	-1.446249	-5.137365	4.015730
H	0.100981	-4.585158	4.670871
H	-1.417612	-3.912054	5.289972
H	0.006902	-2.909737	1.306951
H	0.890210	-4.062231	2.324752
H	-0.670657	-4.510325	1.607796
H	-1.366006	-4.259197	-4.747222
H	-0.588928	-3.586538	-6.200423
H	-2.154640	-2.977518	-5.670793
H	0.042969	-1.454856	-6.347087
H	1.031334	0.277115	-7.075851
H	1.782789	1.499922	-6.026232
H	0.070239	1.596891	-6.394909
H	-2.802086	-1.447852	-3.355647
H	-4.251019	-3.170013	-4.485838
H	-5.129387	-1.682753	-4.075462
H	-5.344971	-3.139157	-3.096458
H	-4.629057	-1.845936	-0.927116
H	-4.582865	-0.408378	-1.965078
H	-3.179319	-0.837570	-0.974597
H	-4.313191	-4.230515	-1.362980
H	-3.057751	-6.225155	-0.616094
H	-0.642464	-6.405491	-1.092735
H	1.396452	-3.672562	-2.565285
H	2.294327	-5.612988	-3.854954
H	0.787922	-5.002920	-4.553438
H	0.773801	-6.486960	-3.586772
H	1.552467	-4.835824	-0.277190
H	2.842259	-5.244142	-1.426380
H	1.530456	-6.383586	-1.132529
H	3.054836	0.280566	-3.621335
H	4.975777	2.455341	-4.612316
H	3.941370	1.454946	-5.641460
H	5.241735	0.706740	-4.700631
H	5.070067	0.456878	-2.201147
H	3.664923	1.087554	-1.320792
H	4.839431	2.202970	-2.026656
H	3.766243	3.923774	-3.143832
H	2.018758	5.670487	-3.088646
H	-0.351654	5.045740	-3.409721

H	-1.519403	1.556141	-4.075100
H	-2.015902	4.467262	-4.902396
H	-3.025355	3.066378	-5.284357
H	-1.400304	3.209589	-5.980716
H	-1.964418	2.336715	-1.757818
H	-3.345496	2.676030	-2.816728
H	-2.246394	3.984968	-2.353182
H	1.687158	-1.156362	1.441793
H	2.354598	0.221586	0.594119
H	1.992747	-2.600375	-0.527600
H	4.454940	-2.566280	-0.816211
H	4.547343	-1.020004	0.021054
H	3.674320	-2.088340	2.107295
H	3.524410	-3.626468	1.268034
H	6.150941	-2.106918	1.697138
H	6.001825	-3.647759	0.868886
H	5.290153	-3.160029	3.825742
H	6.738537	-3.990476	3.235923
H	5.140860	-4.712945	2.991085
H	1.452419	2.911067	-0.180250
H	0.912567	2.785262	-0.676774

168

[TS-**F3F4**] scf done: -4071.100274 / Energies= -4069.537193 / Enthalpies= -4069.536249 / Free Energies= -4069.749941

C	-3.966611	-0.653117	2.988912
C	-2.654769	-1.093905	3.294910
C	-2.368837	-2.481918	3.346138
C	-3.415329	-3.391670	3.163314
C	-4.711905	-2.965272	2.900427
C	-4.972710	-1.604359	2.796845
N	-1.619802	-0.134289	3.514480
C	-1.439243	0.321664	4.752858
C	-2.276919	-0.221613	5.898285
C	-0.964707	-3.013656	3.602831
C	-0.564258	-4.088148	2.581352
C	-4.308529	0.822721	2.842872
C	-4.672443	1.158816	1.387863
C	-0.559553	1.357569	5.127345
C	0.105041	2.350204	4.375744
C	0.659831	3.481814	5.226461
N	0.213731	2.377799	3.051702
C	0.737004	3.544773	2.418123
C	2.091743	3.583206	2.000329
C	2.557091	4.718543	1.330558
C	1.721422	5.795839	1.058985
C	0.394585	5.749279	1.468193
C	-0.119106	4.642794	2.151669
C	3.058760	2.447318	2.306129
C	3.959420	2.089847	1.117201
C	-1.575715	4.665252	2.595690
C	-2.534974	4.667099	1.396807
C	3.924044	2.758777	3.538686
C	-1.876436	5.859887	3.514270
C	-0.797594	-3.567027	5.026979
C	-5.432214	1.263170	3.792122
Ca	-0.412299	0.590341	1.600256
Ca	0.271301	-0.459609	-1.755128
N	-0.351221	-2.150365	-3.317004
C	-0.505745	-3.443053	-2.738400
C	-1.714378	-3.823326	-2.104427
C	-1.766933	-5.054526	-1.440075
C	-0.673274	-5.909618	-1.407751
C	0.500602	-5.544801	-2.060142
C	0.611344	-4.323535	-2.730009
C	-2.969002	-2.962633	-2.157671
C	-3.404456	-2.480745	-0.768491

C	1.887521	-3.977695	-3.487429
C	3.163993	-4.526229	-2.839181
C	-0.717685	-1.924423	-4.575525
C	-1.498135	-2.970832	-5.353447
C	-0.456938	-0.742948	-5.296737
C	0.293881	0.406507	-4.974202
C	0.477229	1.383737	-6.122692
N	0.806015	0.675985	-3.777201
C	1.620546	1.833747	-3.603452
C	3.031176	1.683559	-3.584840
C	3.826186	2.808323	-3.348104
C	3.261198	4.057607	-3.116461
C	1.878878	4.194417	-3.127751
C	1.038762	3.104498	-3.375411
C	3.682916	0.336035	-3.862269
C	4.924993	0.070632	-3.002472
C	-0.468326	3.318005	-3.377087
C	-0.998463	3.569894	-1.959013
C	-0.898204	4.458553	-4.312069
C	4.032756	0.180404	-5.351208
C	-4.128723	-3.704612	-2.842709
C	1.814548	-4.457271	-4.947095
C	1.440668	-1.145992	0.819746
C	2.471268	-1.680252	-0.176536
C	3.756293	-2.273448	0.422834
C	3.534015	-3.507668	1.295241
C	4.830875	-4.080887	1.869266
C	4.609196	-5.314165	2.742427
H	2.802332	-0.861617	-0.853683
H	-1.264611	0.225347	-0.368574
H	1.367786	4.109970	4.683990
H	-0.157848	4.123934	5.575362
H	1.150612	3.076953	6.117531
H	-0.512981	1.525617	6.199185
H	-3.212831	0.346672	5.969511
H	-2.551972	-1.268864	5.762767
H	-1.753124	-0.109033	6.851465
H	2.456364	1.563617	2.551757
H	3.318576	2.922437	4.434917
H	4.611739	1.928774	3.744075
H	4.526060	3.660389	3.370276
H	4.692043	2.879393	0.911133
H	4.527335	1.178044	1.339123
H	3.376418	1.923455	0.206477
H	3.595407	4.759301	1.012726
H	2.104352	6.667541	0.533978
H	-0.258360	6.594520	1.261254
H	-1.766301	3.752291	3.169370
H	-2.902280	5.799067	3.897910
H	-1.194285	5.893759	4.370149
H	-1.781566	6.812427	2.979016
H	-2.379036	3.795183	0.752597
H	-3.577676	4.656539	1.737489
H	-2.396116	5.561745	0.777964
H	-3.413464	1.397506	3.106067
H	-6.379887	0.765238	3.554264
H	-5.191926	1.035485	4.836264
H	-5.599385	2.344059	3.710648
H	-4.873897	2.231125	1.276741
H	-3.866289	0.888589	0.695552
H	-5.571716	0.616006	1.071979
H	-5.982687	-1.271428	2.567882
H	-5.511264	-3.689805	2.765003
H	-3.208545	-4.457267	3.227117
H	-0.270847	-2.170813	3.497287
H	-1.500756	-4.388902	5.210805
H	0.217979	-3.956831	5.169062
H	-0.970302	-2.800566	5.788114

H	-0.736654	-3.760453	1.550923
H	0.497317	-4.338691	2.688441
H	-1.131573	-5.015142	2.727885
H	-1.372532	-3.974847	-4.942895
H	-1.204667	-2.978037	-6.407236
H	-2.567707	-2.728974	-5.316302
H	-0.839203	-0.753046	-6.312864
H	0.410102	0.873386	-7.087007
H	1.433986	1.908116	-6.063692
H	-0.309616	2.147455	-6.096328
H	-2.740416	-2.075407	-2.759251
H	-3.844379	-4.089889	-3.827407
H	-4.985702	-3.032435	-2.972241
H	-4.467284	-4.555818	-2.239732
H	-3.629032	-3.320676	-0.101881
H	-4.309668	-1.866601	-0.844706
H	-2.632739	-1.867259	-0.292784
H	-2.689421	-5.349749	-0.945385
H	-0.736163	-6.861726	-0.886446
H	1.346671	-6.226616	-2.047866
H	1.962221	-2.883029	-3.513978
H	2.739669	-4.204948	-5.480255
H	0.982784	-3.997212	-5.486335
H	1.685357	-5.546053	-4.989929
H	3.228083	-4.276816	-1.775258
H	4.044907	-4.107841	-3.340013
H	3.232940	-5.616924	-2.932085
H	2.939026	-0.436521	-3.628281
H	4.741555	0.956991	-5.664806
H	3.144680	0.258344	-5.985541
H	4.495749	-0.796305	-5.540090
H	5.254701	-0.967532	-3.128561
H	4.728500	0.240072	-1.938196
H	5.767277	0.710539	-3.291649
H	4.908260	2.706117	-3.343017
H	3.896292	4.919624	-2.927416
H	1.439260	5.171018	-2.939850
H	-0.937574	2.397457	-3.741268
H	-0.550827	5.432214	-3.946794
H	-1.992198	4.505629	-4.374541
H	-0.503315	4.326750	-5.325392
H	-0.771332	2.729082	-1.294175
H	-2.087391	3.703257	-1.974570
H	-0.550430	4.468644	-1.519796
H	0.767813	-1.954253	1.142546
H	1.975792	-0.804732	1.725594
H	2.009525	-2.452691	-0.815607
H	4.452859	-2.523018	-0.390960
H	4.249392	-1.491338	1.018588
H	2.853742	-3.252593	2.119716
H	3.020529	-4.283937	0.707757
H	5.340764	-3.303226	2.456150
H	5.513133	-4.334280	1.044942
H	3.960285	-5.084003	3.596687
H	5.555125	-5.700961	3.138651
H	4.131594	-6.121606	2.173575
H	1.238432	0.229691	0.178342
H	1.210535	1.073019	-0.261147

168

[**F4**] scf done: -4071.174449 / Energies= -4069.606568 / Enthalpies= -4069.605623 / Free Energies= -4069.831178

C	-4.009204	-0.985792	2.857722
C	-2.643784	-1.335261	3.015153
C	-2.224701	-2.674548	2.813346
C	-3.175233	-3.626247	2.432569
C	-4.512825	-3.288025	2.261382
C	-4.919474	-1.976998	2.479602

N	-1.686267	-0.313850	3.280482
C	-1.352543	-0.030891	4.535587
C	-1.938125	-0.855784	5.664247
C	-0.775741	-3.101585	3.007066
C	-0.106025	-3.452262	1.670889
C	-4.510705	0.430451	3.107672
C	-5.076476	1.071008	1.831898
C	-0.493875	1.016369	4.924398
C	0.091276	2.050773	4.167527
C	0.834350	3.106992	4.961173
N	0.017097	2.161493	2.844493
C	0.553752	3.317007	2.205394
C	1.901471	3.332269	1.766128
C	2.362850	4.439896	1.049133
C	1.534839	5.522218	0.773764
C	0.218222	5.505977	1.219044
C	-0.295795	4.419392	1.933021
C	2.853383	2.179523	2.055414
C	3.164518	1.376327	0.784451
C	-1.740903	4.461018	2.411615
C	-2.729777	4.513072	1.237539
C	4.157164	2.649563	2.718106
C	-1.991402	5.634490	3.371261
C	-0.641506	-4.274123	3.990615
C	-5.555431	0.476736	4.233245
Ca	-0.876447	0.638290	1.273658
Ca	-0.917051	0.115721	-2.221643
N	-1.910237	-1.273538	-3.854581
C	-2.606945	-2.348185	-3.230989
C	-3.958619	-2.189736	-2.834327
C	-4.569304	-3.213908	-2.104456
C	-3.885280	-4.379099	-1.777208
C	-2.567235	-4.537174	-2.189939
C	-1.908146	-3.542126	-2.917457
C	-4.761059	-0.945996	-3.191967
C	-5.082584	-0.100483	-1.951553
C	-0.472436	-3.773069	-3.370420
C	0.495120	-3.874671	-2.182429
C	-1.908232	-1.160378	-5.178663
C	-2.718713	-2.134123	-6.010441
C	-1.189769	-0.186969	-5.900537
C	-0.280576	0.794762	-5.459326
C	0.404985	1.594832	-6.548835
N	0.013879	1.042201	-4.186716
C	1.013453	2.006470	-3.867577
C	2.355471	1.588512	-3.679128
C	3.299721	2.527644	-3.253673
C	2.948663	3.852016	-3.017483
C	1.633613	4.256629	-3.216265
C	0.651532	3.359213	-3.645339
C	2.795632	0.154396	-3.942786
C	3.252903	-0.549166	-2.656437
C	-0.769672	3.857664	-3.870651
C	-1.464897	4.203573	-2.545997
C	-0.819018	5.059893	-4.825705
C	3.897886	0.078688	-5.010420
C	-6.054135	-1.291612	-3.946648
C	-0.348179	-5.015622	-4.265225
C	5.118888	-3.695145	2.592890
C	6.389444	-2.958984	2.168576
C	7.409717	-2.751257	3.293542
C	8.010124	-4.039983	3.859542
C	9.084924	-3.790372	4.919256
C	9.680843	-5.076983	5.486934
H	6.112317	-1.977245	1.760597
H	-1.972958	1.164436	-0.572997
H	1.883953	3.160284	4.652300
H	0.408003	4.099872	4.781992

H	0.798813	2.902407	6.033554	H	0.416812	-4.489700	4.180480
H	-0.314121	1.080364	5.991661	H	-1.120771	-4.057820	4.951955
H	-3.031557	-0.787028	5.664500	H	-0.107260	-2.604126	0.976243
H	-1.694442	-1.916536	5.544112	H	0.937515	-3.751664	1.829412
H	-1.570798	-0.522742	6.637551	H	-0.623886	-4.282954	1.176647
H	2.353218	1.504735	2.759442	H	-2.461378	-3.169621	-5.764765
H	3.966422	3.244978	3.618124	H	-2.558400	-1.978866	-7.079757
H	4.767960	1.785848	3.006968	H	-3.788803	-2.022100	-5.802713
H	4.759458	3.263209	2.037736	H	-1.317200	-0.235180	-6.976149
H	3.634374	2.009101	0.022319	H	0.069329	1.290069	-7.542634
H	3.848684	0.548189	1.008414	H	1.491965	1.469475	-6.495853
H	2.260190	0.946307	0.337701	H	0.210356	2.666057	-6.429047
H	3.392958	4.455719	0.701652	H	-4.143113	-0.334223	-3.859103
H	1.915684	6.375993	0.218689	H	-5.859446	-1.913652	-4.827369
H	-0.426763	6.357175	1.011458	H	-6.554668	-0.375395	-4.282295
H	-1.932007	3.536531	2.967600	H	-6.760129	-1.836357	-3.308387
H	-3.018850	5.602450	3.753970	H	-5.684014	-0.669721	-1.232732
H	-1.311419	5.609163	4.229824	H	-5.651518	0.794978	-2.231886
H	-1.855384	6.599824	2.868784	H	-4.174300	0.229060	-1.433544
H	-2.607422	3.657524	0.563483	H	-5.603137	-3.096346	-1.788004
H	-3.763076	4.509246	1.605809	H	-4.379046	-5.162170	-1.207378
H	-2.593220	5.424384	0.642848	H	-2.037494	-5.455146	-1.944583
H	-3.653692	1.032183	3.429802	H	-0.172477	-2.906351	-3.969539
H	-6.465243	-0.070888	3.959148	H	0.679200	-5.117933	-4.635365
H	-5.172356	0.038544	5.161296	H	-1.015108	-4.958425	-5.132422
H	-5.844295	1.513751	4.443684	H	-0.595116	-5.932828	-3.717104
H	-5.402148	2.099470	2.030312	H	0.464834	-2.979081	-1.551003
H	-4.334237	1.101435	1.026114	H	1.525659	-4.003322	-2.535867
H	-5.944401	0.513942	1.459114	H	0.252525	-4.733720	-1.545409
H	-5.968551	-1.716160	2.357393	H	1.927801	-0.391496	-4.328945
H	-5.235248	-4.044054	1.964178	H	4.820749	0.564978	-4.672006
H	-2.860954	-4.654342	2.268355	H	3.592423	0.562399	-5.944739
H	-0.233673	-2.250475	3.434724	H	4.139371	-0.967489	-5.234706
H	-1.096392	-5.189423	3.593645	H	3.531769	-1.589223	-2.866371

H 2.467022 -0.557264 -1.892198
H 4.128209 -0.051415 -2.221508
H 4.331435 2.214590 -3.108296
H 3.697401 4.566924 -2.685410
H 1.362653 5.294130 -3.035964
H -1.335572 3.044240 -4.338792
H -0.336603 5.942992 -4.390141
H -1.859951 5.328417 -5.042591
H -0.319028 4.845570 -5.776874
H -1.528510 3.337357 -1.876722
H -2.487239 4.557926 -2.728569
H -0.923990 4.991579 -2.008865
H 5.330726 -4.718570 2.922350
H 4.620298 -3.176114 3.421632
H 6.869155 -3.504902 1.343182
H 8.225969 -2.117825 2.916701
H 6.937989 -2.182900 4.109724
H 7.217533 -4.663206 4.296951
H 8.442466 -4.630445 3.037143
H 8.655032 -3.193546 5.736513
H 9.885892 -3.175238 4.484488
H 8.908882 -5.695943 5.960843
H 10.447583 -4.866388 6.241414
H 10.147302 -5.679613 4.697684
H 4.405226 -3.758365 1.763305
H 0.169145 -0.427881 -0.371359

18

[Hexene]

C 21.277124 13.336836 5.541683
C 20.985690 13.275225 4.071232
C 22.047551 13.986662 3.220210
C 21.775655 13.896538 1.718092
C 22.833305 14.604485 0.873881
C 20.492300 13.900249 6.460605
H 20.763682 13.918784 7.513228
H 19.543202 14.362729 6.194081
H 22.220988 12.887179 5.857885
H 20.931358 12.221713 3.754490
H 19.997568 13.711596 3.871689
H 22.101549 15.041617 3.523370
H 23.036469 13.556352 3.437517
H 20.786686 14.325938 1.503089
H 21.717576 12.838933 1.423038
H 22.892368 15.670786 1.124612
H 22.610019 14.526365 -0.196352
H 23.827670 14.171388 1.038678

20

[hexane] scf done: -236.997222 / Energies= -236.798164 / Enthalpies= -236.797219 / Free Energies= -236.838809

C 4.839661 -3.514738 2.443914
C 6.083296 -2.727327 2.032233
C 7.112334 -2.534420 3.151929
C 7.756976 -3.826377 3.658916
C 8.839421 -3.586721 4.713141
C 9.481855 -4.876147 5.220441
H 5.774080 -1.738717 1.666165
H 5.084471 -4.543049 2.732562
H 4.337104 -3.041493 3.297087
H 6.566820 -3.227445 1.180529
H 7.905003 -1.863189 2.790582
H 6.635637 -2.012320 3.995545
H 6.988814 -4.489018 4.081804
H 8.194294 -4.371651 2.808419
H 8.404350 -3.036009 5.559489
H 9.615193 -2.930834 4.292494
H 8.736075 -5.536542 5.680081
H 10.253527 -4.672427 5.971787

H 9.953971 -5.432690 4.401232
H 4.116958 -3.568666 1.621549

148
[A]
Ca 22.087289 19.587777 7.936914
H 21.114637 19.853472 5.957691
N 22.741374 21.277936 9.451527
N 21.089736 18.780910 9.922578
C 22.835629 22.721020 11.438986
H 22.530411 23.635186 10.918305
H 22.448446 22.758489 12.459702
H 23.929965 22.739309 11.481324
C 22.344619 21.490787 10.702119
C 21.492154 20.639001 11.432092
H 21.262259 20.980114 12.435252
C 20.955020 19.379067 11.102005
C 20.198994 18.674059 12.210181
H 20.717189 17.753375 12.502040
H 20.099881 19.307610 13.094549
H 19.199356 18.375762 11.878231
C 23.680555 22.161609 8.845334
C 25.064058 21.853818 8.899811
C 25.595029 20.676205 9.706912
H 24.740109 20.205191 10.204377
C 26.573442 21.127069 10.802100
H 26.121092 21.868642 11.469527
H 26.885358 20.270086 11.411467
H 27.478166 21.575023 10.373704
C 26.252537 19.617542 8.809472
H 27.125058 20.026972 8.286125
H 26.594991 18.765106 9.409137
H 25.560327 19.238858 8.048541
C 25.963051 22.666734 8.202966
H 27.025982 22.438258 8.242428
C 25.526951 23.760304 7.463989
H 26.241109 24.378341 6.925541
C 24.170415 24.061340 7.423653
H 23.832590 24.920004 6.848450
C 23.230324 23.286479 8.109057
C 21.759168 23.675880 8.054525
H 21.220841 23.037493 8.764228
C 21.529878 25.134026 8.481299
H 20.455872 25.348872 8.535991
H 21.968153 25.345729 9.463008
H 21.968839 25.838709 7.764955
C 21.158972 23.431191 6.662433
H 21.219967 22.376901 6.367554
H 20.100852 23.721383 6.642865
H 21.684453 24.015654 5.897797
C 20.575668 17.465741 9.731196
C 19.260181 17.280325 9.235790
C 18.303627 18.449024 9.041073
H 18.780113 19.340908 9.463202
C 16.975810 18.241963 9.785986
H 16.403808 17.405110 9.367780
H 17.132677 18.035831 10.850714
H 16.351542 19.139826 9.703373
C 18.039064 18.723679 7.554061
H 17.354469 19.572858 7.434190
H 18.960937 18.960336 7.009823
H 17.584834 17.852604 7.067004
C 18.833089 15.984699 8.931032
H 17.828783 15.834574 8.541977
C 19.662819 14.884917 9.116736
H 19.310929 13.885985 8.871569
C 20.942735 15.071958 9.626004
H 21.586043 14.208925 9.783813

C	21.420480	16.346635	9.944065	H	17.081912	16.642319	2.063760
C	22.820676	16.498074	10.522675	C	18.249549	18.630700	3.633812
H	22.958527	17.554108	10.779563	H	17.392894	18.191624	4.159247
C	23.000166	15.684307	11.813493	H	17.877604	19.473773	3.038623
H	22.933845	14.606285	11.623383	H	18.931894	19.029141	4.393521
H	23.985035	15.881057	12.254272	C	18.635314	15.587739	4.224850
H	22.239015	15.935689	12.560345	H	17.565887	15.784681	4.191588
C	23.904810	16.121092	9.502657	C	19.106816	14.501497	4.952684
H	23.833654	16.719869	8.587123	H	18.413704	13.858430	5.489240
H	24.904086	16.273153	9.928939	C	20.471902	14.240346	4.984551
H	23.824122	15.067009	9.210881	H	20.837461	13.386087	5.549573
Ca	22.402006	18.791934	4.507375	C	21.385919	15.050230	4.304414
H	23.370681	18.517261	6.487735	C	22.868381	14.705629	4.352228
N	21.808735	17.088001	2.982890	H	23.385640	15.367838	3.648852
N	23.363713	19.654791	2.526724	C	23.141833	13.259807	3.909980
C	21.768234	15.659193	0.983649	H	24.221861	13.078876	3.851190
H	22.086326	14.750067	1.505055	H	22.708216	13.044670	2.926975
H	22.171210	15.635607	-0.031362	H	22.726327	12.534655	4.619833
H	20.675250	15.616136	0.924297	C	23.462660	14.954235	5.746038
C	22.217206	16.898223	1.732415	H	23.362630	16.001520	6.054481
C	23.042608	17.784082	1.011746	H	24.530722	14.702756	5.760370
H	23.289961	17.455726	0.008488	H	22.960806	14.339681	6.503018
C	23.527650	19.063718	1.347145	C	23.816275	20.992855	2.716015
C	24.261224	19.799143	0.243433	C	25.122534	21.240556	3.208682
H	23.714163	20.703983	-0.045114	C	26.130877	20.117526	3.409767
H	24.382709	19.173330	-0.643670	H	25.702773	19.206457	2.976781
H	25.250203	20.128174	0.578562	C	27.457111	20.391289	2.684306
C	20.899327	16.168063	3.580523	H	27.986748	21.247497	3.118531
C	19.506834	16.432746	3.531358	H	27.303956	20.602376	1.619988
C	18.937568	17.596837	2.730752	H	28.119533	19.521023	2.765197
H	19.776386	18.096213	2.233386	C	26.386481	19.847717	4.899258
C	17.971568	17.119830	1.635688	H	27.117790	19.039437	5.025798
H	18.445834	16.394936	0.965188	H	25.471306	19.552205	5.425859
H	17.632263	17.968920	1.029930	H	26.777444	20.740775	5.400984

C	25.490785	22.555997	3.505565
H	26.486972	22.753832	3.893966
C	24.612449	23.616111	3.312824
H	24.919869	24.631775	3.549220
C	23.341585	23.368152	2.807104
H	22.660641	24.200601	2.643359
C	22.920827	22.070970	2.499549
C	21.526404	21.854498	1.927712
H	21.430543	20.790383	1.685476
C	21.309933	22.642441	0.626518
H	21.333665	23.724809	0.801505
H	20.332086	22.400160	0.192853
H	22.077582	22.411214	-0.120168
C	20.432033	22.201839	2.947413
H	20.530781	21.617303	3.869480
H	19.437890	22.005861	2.527093
H	20.472791	23.261855	3.225884

166

[B]

C	-1.811310	-2.813250	2.652569
C	-2.282045	-1.493331	2.868220
C	-3.662007	-1.198141	2.725278
C	-4.536461	-2.224738	2.356770
C	-4.079913	-3.517485	2.128314
C	-2.727140	-3.800926	2.278130
N	-1.365539	-0.433711	3.130123
C	-1.028973	-0.142014	4.382070
C	-1.545984	-1.009197	5.512660
C	-4.217136	0.197318	2.978183
C	-4.843974	0.802178	1.713510
C	-0.343609	-3.181721	2.822356
C	0.323819	-3.479535	1.471904
C	-0.221837	0.948642	4.766132
C	0.293044	2.022383	4.009408
C	0.985835	3.113669	4.800684
N	0.197609	2.134941	2.689213
C	0.654011	3.320926	2.042666
C	1.991076	3.417769	1.581644
C	2.369034	4.545934	0.847516
C	1.470617	5.572031	0.578791
C	0.166231	5.478368	1.049764
C	-0.266613	4.367062	1.779266
C	3.021713	2.334978	1.868173
C	3.380365	1.548275	0.599279
C	-1.702743	4.323819	2.283967
C	-2.715708	4.329682	1.129686
C	4.293990	2.899069	2.518511
C	-1.998774	5.474277	3.258538
C	-5.229996	0.208099	4.133290
C	-0.148646	-4.365175	3.782324
Ca	-0.609064	0.561006	1.125210
C	3.665191	-0.447764	5.395283
C	4.363789	-1.345816	4.698584
C	5.825116	-1.637172	4.875002
C	6.101284	-3.088882	5.292242
C	7.591221	-3.402529	5.433824
C	7.863129	-4.845102	5.855107
Ca	-0.640755	-0.025215	-2.349794
N	0.117763	0.966066	-4.353866
C	-0.213891	0.673971	-5.607317
C	-1.023127	-0.413197	-5.993835
C	-1.553066	-1.477592	-5.237290
N	-1.466307	-1.588314	-3.915244
C	-1.939337	-2.768168	-3.270477
C	-3.277803	-2.846200	-2.809600
C	-3.672574	-3.968850	-2.075957
C	-2.788960	-5.007691	-1.806875
C	-1.483334	-4.932889	-2.277831

C	-1.034547	-3.828343	-3.007890
C	-4.291900	-1.747055	-3.094980
C	-4.645832	-0.962704	-1.823328
C	0.402032	-3.805599	-3.512759
C	1.414232	-3.805021	-2.357728
C	1.028545	2.029332	-4.088124
C	0.549582	3.346731	-3.874425
C	1.458435	4.339127	-3.495615
C	2.812007	4.062597	-3.339661
C	3.276588	2.772290	-3.566072
C	2.409302	1.741198	-3.939028
C	-0.919525	3.706663	-4.051055
C	-1.594747	4.003946	-2.704349
C	2.971884	0.348335	-4.189668
C	3.587562	-0.257686	-2.919968
C	0.312923	1.537586	-6.736058
C	3.996896	0.343360	-5.334006
C	-1.117153	4.886759	-5.014560
C	-2.249851	-2.565954	-6.029190
C	-5.568439	-2.290481	-3.754800
C	0.688732	-4.971920	-4.471122
H	0.514848	-0.432628	-0.510845
H	-1.766431	0.963534	-0.712351
H	2.038894	3.193711	4.509036
H	0.531714	4.089465	4.599096
H	0.938982	2.922881	5.875162
H	-0.037514	1.019608	5.832307
H	-2.640957	-1.035121	5.512315
H	-1.211309	-2.045377	5.394527
H	-1.207723	-0.645169	6.485343
H	2.577987	1.629973	2.579658
H	4.065717	3.500706	3.405546
H	4.953900	2.079812	2.826499
H	4.856143	3.534900	1.823892
H	3.806547	2.206945	-0.166797
H	4.118361	0.768712	0.825749
H	2.504531	1.057835	0.157760
H	3.390323	4.622573	0.482347
H	1.787136	6.442455	0.009367
H	-0.534103	6.286249	0.848426
H	-1.831665	3.385967	2.835363
H	-3.017375	5.386549	3.655968
H	-1.305550	5.476013	4.106795
H	-1.918958	6.449464	2.763057
H	-2.557499	3.488687	0.444978
H	-3.739463	4.262764	1.517764
H	-2.644619	5.252289	0.540923
H	-3.378009	0.837432	3.272083
H	-6.116577	-0.392059	3.895463
H	-4.796648	-0.192828	5.056036
H	-5.567403	1.231812	4.336389
H	-5.203769	1.819033	1.912696
H	-4.125608	0.853658	0.887369
H	-5.700105	0.209240	1.369801
H	-5.596760	-2.006175	2.249471
H	-4.775016	-4.301760	1.839010
H	-2.373004	-4.813874	2.102256
H	0.166523	-2.316153	3.260098
H	-0.562870	-5.292905	3.370002
H	0.919519	-4.535051	3.962952
H	-0.631389	-4.189612	4.750208
H	0.282581	-2.618872	0.793973
H	1.380270	-3.740787	1.612903
H	-0.166863	-4.318873	0.965235
H	-3.296388	-2.662530	-5.720148
H	-1.781808	-3.539398	-5.847650
H	-2.223096	-2.362369	-7.102019
H	-1.208241	-0.481434	-7.059962

H	-0.019957	1.172369	-7.710187
H	1.408005	1.560423	-6.728806
H	-0.019223	2.575079	-6.622186
H	-3.831492	-1.045207	-3.799726
H	-5.346500	-2.872842	-4.656175
H	-6.229616	-1.463322	-4.040073
H	-6.131095	-2.939365	-3.073057
H	-5.087342	-1.619342	-1.064326
H	-5.369892	-0.169441	-2.048030
H	-3.764521	-0.489762	-1.374052
H	-4.694717	-4.030934	-1.710319
H	-3.117987	-5.873178	-1.237079
H	-0.794677	-5.750664	-2.076097
H	0.540026	-2.876567	-4.076633
H	1.706838	-4.895751	-4.872142
H	-0.006571	-4.982178	-5.317646
H	0.604959	-5.939585	-3.961767
H	1.263843	-2.953524	-1.684217
H	2.438937	-3.753082	-2.745703
H	1.333377	-4.718888	-1.756678
H	2.137874	-0.293672	-4.493872
H	4.880027	0.943948	-5.084753
H	3.572748	0.746891	-6.259909
H	4.338183	-0.679090	-5.537071
H	3.952661	-1.272963	-3.117891
H	2.861287	-0.313336	-2.100985
H	4.438081	0.337455	-2.566318
H	4.337505	2.559408	-3.453499
H	3.501601	4.850419	-3.046829
H	1.098179	5.350116	-3.320819
H	-1.423135	2.837267	-4.488851
H	-0.708334	5.817446	-4.603490
H	-2.185731	5.051241	-5.198093
H	-0.630824	4.711022	-5.980665
H	-1.549366	3.145884	-2.023371
H	-2.652635	4.255940	-2.850958
H	-1.112761	4.848677	-2.198301
H	2.603264	-0.285152	5.225669
H	4.137389	0.165527	6.161863
H	3.849605	-1.941163	3.940563
H	6.352856	-1.439429	3.928467
H	6.251627	-0.951007	5.619273
H	5.589056	-3.291414	6.243354
H	5.654186	-3.770869	4.553646
H	8.037276	-2.715978	6.167628
H	8.097025	-3.196850	4.479418
H	7.397560	-5.069632	6.822679
H	8.937656	-5.039309	5.949928
H	7.459965	-5.555086	5.122394

166

[TS-BC]

C	19.956321	16.515460	9.345596
C	19.813007	17.926225	9.356147
C	18.564547	18.503131	9.014016
C	17.499284	17.666919	8.667433
C	17.635313	16.283910	8.652976
C	18.860306	15.722583	8.990524
N	20.922801	18.774322	9.650092
C	21.122683	19.163793	10.904288
C	20.272705	18.580054	12.018816
C	18.346496	20.007835	9.000619
C	18.083161	20.510606	7.574193
C	21.262076	15.826423	9.711992
C	21.843231	15.064990	8.512533
C	22.050735	20.146385	11.327146
C	22.715568	21.193712	10.649101
C	23.395121	22.201794	11.562499
N	22.787464	21.347290	9.328031

C	23.248050	22.588940	8.797865	C	18.791783	17.880659	4.694045
C	24.620965	22.822752	8.543134	C	23.619047	14.380670	3.636393
C	25.013195	24.044332	7.985054	C	24.512221	14.399376	4.884630
C	24.088260	25.034656	7.685671	C	23.964202	20.769585	2.703684
C	22.740169	24.799800	7.932533	C	23.080065	21.857494	2.922989
C	22.295122	23.591333	8.474855	C	23.614354	23.092631	3.301847
C	25.692286	21.788822	8.850433	C	24.983393	23.272686	3.460632
C	26.299123	21.228311	7.556702	C	25.844715	22.208163	3.221397
C	20.811287	23.386501	8.743759	C	25.363580	20.952962	2.837132
C	19.915516	23.984551	7.652561	C	21.576618	21.731017	2.713557
C	26.805186	22.341118	9.753891	C	20.775723	22.118845	3.963880
C	20.404321	23.946088	10.116722	C	26.353954	19.829175	2.566576
C	17.212733	20.437947	9.943076	C	27.076473	19.392462	3.847870
C	21.112385	14.876806	10.910465	C	23.749603	19.909429	0.042851
Ca	22.638585	19.313004	8.071140	C	27.378613	20.208748	1.486779
C	24.597169	17.935093	8.974653	C	21.112405	22.559610	1.504632
C	24.754443	17.317029	7.724502	C	21.490966	15.654932	0.835178
C	26.103674	17.331159	7.028346	C	17.952601	16.568723	2.712963
C	27.094664	16.364800	7.690194	C	23.866930	13.096707	2.830030
C	28.453728	16.327663	6.989043	H	23.929555	17.966843	6.554762
C	29.438641	15.361201	7.644042	H	21.820855	19.479357	6.043701
Ca	22.829260	18.415768	4.464574	H	24.473328	22.002314	11.595483
N	23.425470	19.474245	2.445749	H	23.272624	23.225438	11.198989
C	23.269862	19.050343	1.196104	H	23.012386	22.141848	12.584110
C	22.687887	17.819982	0.829544	H	22.142600	20.210940	12.406023
C	22.115687	16.797650	1.610363	H	19.279882	19.046512	12.013535
N	22.066876	16.784769	2.940951	H	20.114105	17.506258	11.893638
C	21.413363	15.703867	3.605253	H	20.721266	18.762967	12.998330
C	20.040185	15.814204	3.941575	H	25.212628	20.960272	9.383416
C	19.445138	14.793915	4.689586	H	26.400753	22.792351	10.665925
C	20.165206	13.674261	5.089029	H	27.493554	21.538522	10.045876
C	21.505980	13.562918	4.738977	H	27.396424	23.109105	9.240985
C	22.151493	14.559314	4.000519	H	26.762377	22.024635	6.962385
C	19.197675	17.003268	3.501100	H	27.071301	20.482529	7.780001

H	25.539925	20.755114	6.922349	H	23.561091	20.970641	0.225262
H	26.068369	24.223824	7.790622	H	19.811560	17.613612	2.829007
H	24.412920	25.982466	7.262865	H	18.212069	15.930174	1.861159
H	22.016842	25.575911	7.697161	H	17.422355	17.448175	2.328182
H	20.638972	22.304474	8.765637	H	17.249337	16.011467	3.343123
H	19.329527	23.804571	10.286374	H	18.189178	17.318058	5.416157
H	20.937428	23.449356	10.932619	H	18.199372	18.739816	4.356381
H	20.616507	25.021130	10.177314	H	19.662047	18.270128	5.236085
H	20.197563	23.625647	6.657485	H	18.396140	14.878502	4.962586
H	18.870892	23.705977	7.831611	H	19.682421	12.889634	5.666306
H	19.952971	25.080663	7.642076	H	22.064979	12.681166	5.044654
H	19.268889	20.479371	9.353735	H	23.909618	15.228346	3.005781
H	16.243130	20.042278	9.617053	H	24.916756	13.039070	2.517882
H	17.384171	20.089513	10.967575	H	23.245047	13.054665	1.929410
H	17.130505	21.531416	9.967249	H	23.649503	12.201069	3.424067
H	17.931365	21.595989	7.567373	H	24.378945	15.322482	5.459755
H	18.924378	20.280586	6.911891	H	25.570650	14.321883	4.607253
H	17.184467	20.046579	7.149261	H	24.278744	13.561584	5.552520
H	16.541428	18.111655	8.405793	H	25.786358	18.967560	2.197368
H	16.793271	15.649702	8.385966	H	28.038061	21.017297	1.824463
H	18.970534	14.640404	8.983778	H	26.891608	20.545615	0.565513
H	21.976992	16.604516	9.999374	H	28.012830	19.347992	1.242141
H	20.430481	14.048280	10.683912	H	27.749105	18.549250	3.648406
H	22.084263	14.442766	11.175666	H	26.369881	19.082395	4.625315
H	20.723272	15.392873	11.794429	H	27.678029	20.210107	4.262846
H	21.967351	15.716001	7.640016	H	26.916805	22.354825	3.332742
H	22.820135	14.633518	8.763256	H	25.376370	24.238806	3.766162
H	21.184346	14.245198	8.204373	H	22.942966	23.929791	3.477041
H	20.407407	15.630296	0.998850	H	21.359715	20.679953	2.489707
H	21.876625	14.687965	1.172258	H	21.286333	23.629979	1.669316
H	21.673920	15.750243	-0.237421	H	20.038141	22.418294	1.333977
H	22.644163	17.646512	-0.239952	H	21.639668	22.273955	0.588108
H	23.270176	19.618937	-0.895123	H	21.061995	21.511780	4.829769
H	24.832858	19.796350	-0.086609	H	19.702791	21.974684	3.786801

H	20.927613	23.173016	4.224433
H	24.003352	17.457326	9.751778
H	25.343267	18.651347	9.317228
H	24.231563	16.366621	7.605397
H	25.988910	17.061889	5.969391
H	26.517620	18.346471	7.050476
H	27.227077	16.647523	8.742333
H	26.662981	15.352653	7.698706
H	28.884416	17.339370	6.979552
H	28.313138	16.048300	5.934671
H	29.627357	15.636001	8.689129
H	30.402617	15.354834	7.122184
H	29.049833	14.335333	7.638292

166

[C]

C	-2.221494	-2.583016	3.146236
C	-2.575764	-1.213661	3.239439
C	-3.928811	-0.824930	3.064241
C	-4.891753	-1.807801	2.818512
C	-4.551497	-3.153009	2.737296
C	-3.222831	-3.526760	2.895357
N	-1.580284	-0.210750	3.445904
C	-1.294894	0.159579	4.694268
C	-1.914808	-0.598803	5.853086
C	-4.366736	0.631548	3.128863
C	-4.905464	1.109943	1.772179
C	-0.787196	-3.067167	3.301837
C	-0.267858	-3.679696	1.993471
C	-0.483570	1.250847	5.062592
C	0.032378	2.342497	4.325884
C	0.508959	3.488740	5.203935
N	0.073498	2.432668	3.002456
C	0.473796	3.656265	2.386268
C	1.824160	3.860466	2.002610
C	2.168460	5.054134	1.360093
C	1.219977	6.031267	1.083025
C	-0.103111	5.818374	1.450375
C	-0.500199	4.645813	2.100008
C	2.916728	2.843812	2.303621
C	3.782192	2.532869	1.074752
C	-1.961069	4.484228	2.495227
C	-2.888068	4.516338	1.271668
C	3.813471	3.299970	3.466257
C	-2.390453	5.546809	3.518844
C	-5.405635	0.876600	4.233389
C	-0.626657	-4.076639	4.449247
Ca	-0.471908	0.634960	1.530887
C	1.488241	-0.645321	0.595658
C	2.410959	-1.585743	-0.192578
C	3.789751	-1.897082	0.422082
C	3.739331	-2.690094	1.727054
C	5.120852	-2.951349	2.330065
C	5.068621	-3.761321	3.623976
Ca	0.209101	-0.425028	-1.691634
N	0.954115	0.607935	-3.714160
C	0.496637	0.331288	-4.930478
C	-0.299208	-0.781937	-5.271234
C	-0.743775	-1.895350	-4.530238
N	-0.530897	-2.094405	-3.232243
C	-0.957063	-3.306634	-2.617897
C	-2.286309	-3.458032	-2.150285
C	-2.630928	-4.630764	-1.469054
C	-1.707756	-5.646802	-1.261004
C	-0.407575	-5.497992	-1.734150
C	-0.005533	-4.341683	-2.407861
C	-3.357743	-2.400723	-2.380344
C	-3.775633	-1.715066	-1.072218
C	1.415959	-4.222061	-2.943895

C	2.455808	-4.976890	-2.106950	H	1.510979	6.953444	0.585873
C	1.813995	1.723998	-3.503233	H	-0.846337	6.583130	1.235447
C	1.283817	3.021006	-3.288661	H	-2.070790	3.503815	2.971289
C	2.162626	4.063504	-2.974566	H	-3.421356	5.368595	3.848500
C	3.533683	3.858109	-2.892068	H	-1.744388	5.539187	4.402924
C	4.050195	2.588284	-3.128227	H	-2.349493	6.554577	3.087709
C	3.216228	1.508121	-3.429407	H	-2.637246	3.727640	0.554124
C	-0.201265	3.329048	-3.422496	H	-3.931964	4.378330	1.577422
C	-0.826648	3.745197	-2.084703	H	-2.822250	5.475716	0.744727
C	3.818628	0.143700	-3.734591	H	-3.483454	1.232870	3.369461
C	5.025884	-0.202742	-2.855163	H	-6.337599	0.332284	4.038261
C	0.791628	1.258547	-6.098419	H	-5.035646	0.558640	5.214220
C	4.206094	0.027371	-5.218188	H	-5.653004	1.943350	4.295967
C	-0.459656	4.408673	-4.486081	H	-5.188358	2.168278	1.819727
C	-1.551393	-2.898692	-5.337603	H	-4.160716	0.992945	0.976717
C	-4.592464	-2.981913	-3.087841	H	-5.795297	0.541449	1.475806
C	1.510587	-4.681716	-4.408351	H	-5.930584	-1.511833	2.689337
H	2.628157	-1.174841	-1.200618	H	-5.315859	-3.903594	2.550919
H	-1.261459	0.686142	-0.493251	H	-2.953308	-4.578159	2.825978
H	1.060832	4.247198	4.647410	H	-0.165417	-2.195854	3.539710
H	-0.351344	3.976275	5.677852	H	-1.190636	-4.996724	4.254624
H	1.143076	3.106654	6.010999	H	0.427903	-4.354198	4.565954
H	-0.356607	1.360848	6.135060	H	-0.975482	-3.671789	5.405208
H	-3.001348	-0.455769	5.865777	H	-0.339080	-2.972526	1.160299
H	-1.746933	-1.675556	5.760520	H	0.782247	-3.976588	2.096458
H	-1.514468	-0.262213	6.811974	H	-0.842597	-4.569132	1.712423
H	2.427208	1.913871	2.617030	H	-1.483091	-3.910166	-4.931098
H	3.241666	3.462469	4.384576	H	-1.227770	-2.913170	-6.382004
H	4.581413	2.545096	3.675765	H	-2.610750	-2.613620	-5.327083
H	4.324271	4.238895	3.219350	H	-0.589542	-0.806893	-6.317329
H	4.398918	3.392222	0.786733	H	0.959999	0.686552	-7.015694
H	4.464603	1.703215	1.294419	H	1.657251	1.897906	-5.914871
H	3.177853	2.259856	0.204009	H	-0.071378	1.911868	-6.277793
H	3.203744	5.222850	1.074497	H	-2.934473	-1.630609	-3.034703

H	-4.323934	-3.515280	-4.006056
H	-5.291784	-2.178875	-3.350515
H	-5.131528	-3.685830	-2.442513
H	-4.154878	-2.438475	-0.341993
H	-4.566939	-0.979505	-1.262544
H	-2.938218	-1.179870	-0.611310
H	-3.646728	-4.749111	-1.099350
H	-1.998293	-6.553720	-0.736055
H	0.308554	-6.298851	-1.572886
H	1.678675	-3.156282	-2.928119
H	2.545123	-4.606211	-4.766068
H	0.885696	-4.073585	-5.067328
H	1.194614	-5.727811	-4.506941
H	2.370328	-4.750842	-1.038723
H	3.465973	-4.703025	-2.432760
H	2.365144	-6.063372	-2.227827
H	3.036776	-0.603721	-3.549801
H	4.960047	0.779943	-5.480974
H	3.343873	0.171426	-5.875424
H	4.628237	-0.963101	-5.429418
H	5.309395	-1.251409	-3.003449
H	4.813425	-0.054778	-1.791322
H	5.903632	0.404033	-3.108557
H	5.124885	2.434642	-3.078025
H	4.198867	4.683991	-2.652022
H	1.760903	5.058336	-2.797168
H	-0.705858	2.413597	-3.750849
H	-0.056608	5.380396	-4.176257
H	-1.537428	4.535773	-4.644827
H	-0.000569	4.152117	-5.446645
H	-0.762830	2.938136	-1.347005
H	-1.889082	3.984155	-2.218791
H	-0.332093	4.628986	-1.665622
H	1.266669	-1.168936	1.549158
H	2.107908	0.223371	0.900661
H	1.894372	-2.542762	-0.373347
H	4.395818	-2.454146	-0.308226
H	4.313351	-0.945693	0.597384
H	3.121490	-2.154708	2.461354
H	3.237378	-3.653016	1.546062
H	5.619396	-1.989768	2.519881
H	5.746023	-3.476625	1.593614
H	4.476823	-3.245425	4.390417
H	6.071142	-3.928408	4.034590
H	4.609349	-4.743809	3.457982

168

[C1] scf done: -4071.120272 / Energies= -4069.557019 / Enthalpies= -4069.556075 / Free Energies= -4069.774684

C	-3.768302	-0.717346	2.744794
C	-2.475630	-1.200899	3.072701
C	-2.191237	-2.584659	2.966008
C	-3.198320	-3.450393	2.528159
C	-4.467925	-2.983703	2.210062
C	-4.742431	-1.625436	2.321110
N	-1.456363	-0.273977	3.445932
C	-1.329377	0.045732	4.733342
C	-2.149792	-0.700738	5.768368
C	-0.825230	-3.156472	3.316882
C	-0.109196	-3.689546	2.068187
C	-4.124995	0.759862	2.841342
C	-4.425944	1.354614	1.457616
C	-0.510031	1.069593	5.242982
C	0.235977	2.081016	4.598861
C	0.659222	3.212609	5.521348
N	0.516618	2.125640	3.301508
C	1.208734	3.259025	2.782802
C	2.612822	3.192884	2.584590

C	3.261337	4.294734	2.017876
C	2.560477	5.434374	1.637555
C	1.185322	5.485335	1.825532
C	0.488797	4.416860	2.397957
C	3.419665	1.968991	3.002674
C	4.604295	1.679434	2.073126
C	-1.019364	4.526472	2.574106
C	-1.741609	4.508416	1.218602
C	3.924105	2.075589	4.451347
C	-1.423903	5.772512	3.375500
C	-0.905959	-4.257594	4.385136
C	-5.299229	1.012356	3.798788
Ca	-0.169463	0.539140	1.626557
Ca	0.174534	-0.523919	-1.680167
N	-0.544994	-2.287427	-3.165521
C	-1.225253	-3.362517	-2.518460
C	-2.616368	-3.267433	-2.264057
C	-3.247806	-4.302973	-1.568124
C	-2.546356	-5.424783	-1.145497
C	-1.185012	-5.520036	-1.413606
C	-0.499833	-4.504902	-2.088517
C	-3.451639	-2.091518	-2.752288
C	-3.988669	-1.249674	-1.587008
C	0.984109	-4.673687	-2.391130
C	1.764896	-5.317303	-1.237213
C	-0.507670	-2.258391	-4.492999
C	-1.184249	-3.345241	-5.314318
C	0.072602	-1.242676	-5.283070
C	0.548799	0.045145	-4.975216
C	0.887011	0.895687	-6.186719
N	0.661805	0.564398	-3.753404
C	1.035761	1.937046	-3.631105
C	2.396772	2.296830	-3.460105
C	2.723019	3.643060	-3.270883

C	1.745033	4.629784	-3.243350
C	0.413727	4.272713	-3.421116
C	0.033976	2.942514	-3.626101
C	3.517295	1.266494	-3.495929
C	4.315776	1.252683	-2.185553
C	-1.437821	2.627810	-3.862941
C	-2.293991	2.922617	-2.622808
C	-1.996216	3.384973	-5.078584
C	4.469048	1.485158	-4.682756
C	-4.607140	-2.551038	-3.655420
C	1.219294	-5.488078	-3.674388
C	1.725190	-0.679668	0.439123
C	2.531468	-1.643549	-0.445042
C	3.981041	-1.960662	-0.027903
C	4.112725	-2.696941	1.304438
C	5.560317	-3.034054	1.667347
C	5.691729	-3.764119	3.002484
H	2.607946	-1.260416	-1.485092
H	-1.147066	0.593413	-0.324906
H	1.519107	3.769116	5.144882
H	-0.169639	3.923558	5.628834
H	0.888604	2.832227	6.521194
H	-0.556501	1.179878	6.321283
H	-3.221018	-0.536664	5.607739
H	-1.988630	-1.780745	5.693542
H	-1.900748	-0.381007	6.782687
H	2.745364	1.103687	2.961986
H	3.102186	2.132132	5.169289
H	4.530885	1.198304	4.707949
H	4.549921	2.967649	4.579161
H	5.408274	2.415530	2.195168
H	5.030547	0.697475	2.307334
H	4.307711	1.678466	1.019449
H	4.336796	4.261369	1.867755

H	3.085631	6.276779	1.194197
H	0.638033	6.374899	1.521786
H	-1.354477	3.649836	3.138849
H	-2.504705	5.770946	3.561592
H	-0.911967	5.814274	4.342859
H	-1.185758	6.696347	2.834926
H	-1.521213	3.598908	0.648394
H	-2.828123	4.561497	1.357863
H	-1.440516	5.361614	0.598842
H	-3.252468	1.283923	3.246178
H	-6.223132	0.546087	3.435743
H	-5.099533	0.615730	4.800275
H	-5.488689	2.088513	3.894544
H	-4.637756	2.428096	1.537683
H	-3.583751	1.222280	0.769087
H	-5.301834	0.876322	1.002394
H	-5.736390	-1.259540	2.072818
H	-5.239214	-3.674772	1.878869
H	-2.983008	-4.512313	2.437051
H	-0.220532	-2.340618	3.729724
H	-1.446249	-5.137365	4.015730
H	0.100981	-4.585158	4.670871
H	-1.417612	-3.912054	5.289972
H	0.006902	-2.909737	1.306951
H	0.890210	-4.062231	2.324752
H	-0.670657	-4.510325	1.607796
H	-1.366006	-4.259197	-4.747222
H	-0.588928	-3.586538	-6.200423
H	-2.154640	-2.977518	-5.670793
H	0.042969	-1.454856	-6.347087
H	1.031334	0.277115	-7.075851
H	1.782789	1.499922	-6.026232
H	0.070239	1.596891	-6.394909
H	-2.802086	-1.447852	-3.355647

H	-4.251019	-3.170013	-4.485838
H	-5.129387	-1.682753	-4.075462
H	-5.344971	-3.139157	-3.096458
H	-4.629057	-1.845936	-0.927116
H	-4.582865	-0.408378	-1.965078
H	-3.179319	-0.837570	-0.974597
H	-4.313191	-4.230515	-1.362980
H	-3.057751	-6.225155	-0.616094
H	-0.642464	-6.405491	-1.092735
H	1.396452	-3.672562	-2.565285
H	2.294327	-5.612988	-3.854954
H	0.787922	-5.002920	-4.553438
H	0.773801	-6.486960	-3.586772
H	1.552467	-4.835824	-0.277190
H	2.842259	-5.244142	-1.426380
H	1.530456	-6.383586	-1.132529
H	3.054836	0.280566	-3.621335
H	4.975777	2.455341	-4.612316
H	3.941370	1.454946	-5.641460
H	5.241735	0.706740	-4.700631
H	5.070067	0.456878	-2.201147
H	3.664923	1.087554	-1.320792
H	4.839431	2.202970	-2.026656
H	3.766243	3.923774	-3.143832
H	2.018758	5.670487	-3.088646
H	-0.351654	5.045740	-3.409721
H	-1.519403	1.556141	-4.075100
H	-2.015902	4.467262	-4.902396
H	-3.025355	3.066378	-5.284357
H	-1.400304	3.209589	-5.980716
H	-1.964418	2.336715	-1.757818
H	-3.345496	2.676030	-2.816728
H	-2.246394	3.984968	-2.353182
H	1.687158	-1.156362	1.441793

H	2.354598	0.221586	0.594119
H	1.992747	-2.600375	-0.527600
H	4.454940	-2.566280	-0.816211
H	4.547343	-1.020004	0.021054
H	3.674320	-2.088340	2.107295
H	3.524410	-3.626468	1.268034
H	6.150941	-2.106918	1.697138
H	6.001825	-3.647759	0.868886
H	5.290153	-3.160029	3.825742
H	6.738537	-3.990476	3.235923
H	5.140860	-4.712945	2.991085
H	1.452419	2.911067	-0.180250
H	0.912567	2.785262	-0.676774

168

[TS-C1C2] scf done: -4071.100274 / Energies= -4069.537193 / Enthalpies= -4069.536249 / Free Energies= -4069.749941

C	-3.966611	-0.653117	2.988912
C	-2.654769	-1.093905	3.294910
C	-2.368837	-2.481918	3.346138
C	-3.415329	-3.391670	3.163314
C	-4.711905	-2.965272	2.900427
C	-4.972710	-1.604359	2.796845
N	-1.619802	-0.134289	3.514480
C	-1.439243	0.321664	4.752858
C	-2.276919	-0.221613	5.898285
C	-0.964707	-3.013656	3.602831
C	-0.564258	-4.088148	2.581352
C	-4.308529	0.822721	2.842872
C	-4.672443	1.158816	1.387863
C	-0.559553	1.357569	5.127345
C	0.105041	2.350204	4.375744
C	0.659831	3.481814	5.226461
N	0.213731	2.377799	3.051702
C	0.737004	3.544773	2.418123
C	2.091743	3.583206	2.000329
C	2.557091	4.718543	1.330558
C	1.721422	5.795839	1.058985
C	0.394585	5.749279	1.468193
C	-0.119106	4.642794	2.151669
C	3.058760	2.447318	2.306129
C	3.959420	2.089847	1.117201
C	-1.575715	4.665252	2.595690
C	-2.534974	4.667099	1.396807
C	3.924044	2.758777	3.538686
C	-1.876436	5.859887	3.514270
C	-0.797594	-3.567027	5.026979
C	-5.432214	1.263170	3.792122
Ca	-0.412299	0.590341	1.600256
Ca	0.271301	-0.459609	-1.755128
N	-0.351221	-2.150365	-3.317004
C	-0.505745	-3.443053	-2.738400
C	-1.714378	-3.823326	-2.104427
C	-1.766933	-5.054526	-1.440075
C	-0.673274	-5.909618	-1.407751
C	0.500602	-5.544801	-2.060142
C	0.611344	-4.323535	-2.730009
C	-2.969002	-2.962633	-2.157671
C	-3.404456	-2.480745	-0.768491
C	1.887521	-3.977695	-3.487429
C	3.163993	-4.526229	-2.839181
C	-0.717685	-1.924423	-4.575525
C	-1.498135	-2.970832	-5.353447
C	-0.456938	-0.742948	-5.296737
C	0.293881	0.406507	-4.974202
C	0.477229	1.383737	-6.122692
N	0.806015	0.675985	-3.777201

C	1.620546	1.833747	-3.603452
C	3.031176	1.683559	-3.584840
C	3.826186	2.808323	-3.348104
C	3.261198	4.057607	-3.116461
C	1.878878	4.194417	-3.127751
C	1.038762	3.104498	-3.375411
C	3.682916	0.336035	-3.862269
C	4.924993	0.070632	-3.002472
C	-0.468326	3.318005	-3.377087
C	-0.998463	3.569894	-1.959013
C	-0.898204	4.458553	-4.312069
C	4.032756	0.180404	-5.351208
C	-4.128723	-3.704612	-2.842709
C	1.814548	-4.457271	-4.947095
C	1.440668	-1.145992	0.819746
C	2.471268	-1.680252	-0.176536
C	3.756293	-2.273448	0.422834
C	3.534015	-3.507668	1.295241
C	4.830875	-4.080887	1.869266
C	4.609196	-5.314165	2.742427
H	2.802332	-0.861617	-0.853683
H	-1.264611	0.225347	-0.368574
H	1.367786	4.109970	4.683990
H	-0.157848	4.123934	5.575362
H	1.150612	3.076953	6.117531
H	-0.512981	1.525617	6.199185
H	-3.212831	0.346672	5.969511
H	-2.551972	-1.268864	5.762767
H	-1.753124	-0.109033	6.851465
H	2.456364	1.563617	2.551757
H	3.318576	2.922437	4.434917
H	4.611739	1.928774	3.744075
H	4.526060	3.660389	3.370276
H	4.692043	2.879393	0.911133
H	4.527335	1.178044	1.339123
H	3.376418	1.923455	0.206477
H	3.595407	4.759301	1.012726
H	2.104352	6.667541	0.533978
H	-0.258360	6.594520	1.261254
H	-1.766301	3.752291	3.169370
H	-2.902280	5.799067	3.897910
H	-1.194285	5.893759	4.370149
H	-1.781566	6.812427	2.979016
H	-2.379036	3.795183	0.752597
H	-3.577676	4.656539	1.737489
H	-2.396116	5.561745	0.777964
H	-3.413464	1.397506	3.106067
H	-6.379887	0.765238	3.554264
H	-5.191926	1.035485	4.836264
H	-5.599385	2.344059	3.710648
H	-4.873897	2.231125	1.276741
H	-3.866289	0.888589	0.695552
H	-5.571716	0.616006	1.071979
H	-5.982687	-1.271428	2.567882
H	-5.511264	-3.689805	2.765003
H	-3.208545	-4.457267	3.227117
H	-0.270847	-2.170813	3.497287
H	-1.500756	-4.388902	5.210805
H	0.217979	-3.956831	5.169062
H	-0.970302	-2.800566	5.788114
H	-0.736654	-3.760453	1.550923
H	0.497317	-4.338691	2.688441
H	-1.131573	-5.015142	2.727885
H	-1.372532	-3.974847	-4.942895
H	-1.204667	-2.978037	-6.407236
H	-2.567707	-2.728974	-5.316302
H	-0.839203	-0.753046	-6.312864
H	0.410102	0.873386	-7.087007

H	1.433986	1.908116	-6.063692
H	-0.309616	2.147455	-6.096328
H	-2.740416	-2.075407	-2.759251
H	-3.844379	-4.089889	-3.827407
H	-4.985702	-3.032435	-2.972241
H	-4.467284	-4.555818	-2.239732
H	-3.629032	-3.320676	-0.101881
H	-4.309668	-1.866601	-0.844706
H	-2.632739	-1.867259	-0.292784
H	-2.689421	-5.349749	-0.945385
H	-0.736163	-6.861726	-0.886446
H	1.346671	-6.226616	-2.047866
H	1.962221	-2.883029	-3.513978
H	2.739669	-4.204948	-5.480255
H	0.982784	-3.997212	-5.486335
H	1.685357	-5.546053	-4.989929
H	3.228083	-4.276816	-1.775258
H	4.044907	-4.107841	-3.340013
H	3.232940	-5.616924	-2.932085
H	2.939026	-0.436521	-3.628281
H	4.741555	0.956991	-5.664806
H	3.144680	0.258344	-5.985541
H	4.495749	-0.796305	-5.540090
H	5.254701	-0.967532	-3.128561
H	4.728500	0.240072	-1.938196
H	5.767277	0.710539	-3.291649
H	4.908260	2.706117	-3.343017
H	3.896292	4.919624	-2.927416
H	1.439260	5.171018	-2.939850
H	-0.937574	2.397457	-3.741268
H	-0.550827	5.432214	-3.946794
H	-1.992198	4.505629	-4.374541
H	-0.503315	4.326750	-5.325392
H	-0.771332	2.729082	-1.294175
H	-2.087391	3.703257	-1.974570
H	-0.550430	4.468644	-1.519796
H	0.767813	-1.954253	1.142546
H	1.975792	-0.804732	1.725594
H	2.009525	-2.452691	-0.815607
H	4.452859	-2.523018	-0.390960
H	4.249392	-1.491338	1.018588
H	2.853742	-3.252593	2.119716
H	3.020529	-4.283937	0.707757
H	5.340764	-3.303226	2.456150
H	5.513133	-4.334280	1.044942
H	3.960285	-5.084003	3.596687
H	5.555125	-5.700961	3.138651
H	4.131594	-6.121606	2.173575
H	1.238432	0.229691	0.178342
H	1.210535	1.073019	-0.261147

168

[C2] scf done: -4071.174449 / Energies= -4069.606568 / Enthalpies= -4069.605623 / Free Energies= -4069.831178

C	-4.009204	-0.985792	2.857722
C	-2.643784	-1.335261	3.015153
C	-2.224701	-2.674548	2.813346
C	-3.175233	-3.626247	2.432569
C	-4.512825	-3.288025	2.261382
C	-4.919474	-1.976998	2.479602
N	-1.686267	-0.313850	3.280482
C	-1.352543	-0.030891	4.535587
C	-1.938125	-0.855784	5.664247
C	-0.775741	-3.101585	3.007066
C	-0.106025	-3.452262	1.670889
C	-4.510705	0.430451	3.107672
C	-5.076476	1.071008	1.831898
C	-0.493875	1.016369	4.924398

C	0.091276	2.050773	4.167527	C	0.404985	1.594832	-6.548835
C	0.834350	3.106992	4.961173	N	0.013879	1.042201	-4.186716
N	0.017097	2.161493	2.844493	C	1.013453	2.006470	-3.867577
C	0.553752	3.317007	2.205394	C	2.355471	1.588512	-3.679128
C	1.901471	3.332269	1.766128	C	3.299721	2.527644	-3.253673
C	2.362850	4.439896	1.049133	C	2.948663	3.852016	-3.017483
C	1.534839	5.522218	0.773764	C	1.633613	4.256629	-3.216265
C	0.218222	5.505977	1.219044	C	0.651532	3.359213	-3.645339
C	-0.295795	4.419392	1.933021	C	2.795632	0.154396	-3.942786
C	2.853383	2.179523	2.055414	C	3.252903	-0.549166	-2.656437
C	3.164518	1.376327	0.784451	C	-0.769672	3.857664	-3.870651
C	-1.740903	4.461018	2.411615	C	-1.464897	4.203573	-2.545997
C	-2.729777	4.513072	1.237539	C	-0.819018	5.059893	-4.825705
C	4.157164	2.649563	2.718106	C	3.897886	0.078688	-5.010420
C	-1.991402	5.634490	3.371261	C	-6.054135	-1.291612	-3.946648
C	-0.641506	-4.274123	3.990615	C	-0.348179	-5.015622	-4.265225
C	-5.555431	0.476736	4.233245	C	5.118888	-3.695145	2.592890
Ca	-0.876447	0.638290	1.273658	C	6.389444	-2.958984	2.168576
Ca	-0.917051	0.115721	-2.221643	C	7.409717	-2.751257	3.293542
N	-1.910237	-1.273538	-3.854581	C	8.010124	-4.039983	3.859542
C	-2.606945	-2.348185	-3.230989	C	9.084924	-3.790372	4.919256
C	-3.958619	-2.189736	-2.834327	C	9.680843	-5.076983	5.486934
C	-4.569304	-3.213908	-2.104456	H	6.112317	-1.977245	1.760597
C	-3.885280	-4.379099	-1.777208	H	-1.972958	1.164436	-0.572997
C	-2.567235	-4.537174	-2.189939	H	1.883953	3.160284	4.652300
C	-1.908146	-3.542126	-2.917457	H	0.408003	4.099872	4.781992
C	-4.761059	-0.945996	-3.191967	H	0.798813	2.902407	6.033554
C	-5.082584	-0.100483	-1.951553	H	-0.314121	1.080364	5.991661
C	-0.472436	-3.773069	-3.370420	H	-3.031557	-0.787028	5.664500
C	0.495120	-3.874671	-2.182429	H	-1.694442	-1.916536	5.544112
C	-1.908232	-1.160378	-5.178663	H	-1.570798	-0.522742	6.637551
C	-2.718713	-2.134123	-6.010441	H	2.353218	1.504735	2.759442
C	-1.189769	-0.186969	-5.900537	H	3.966422	3.244978	3.618124
C	-0.280576	0.794762	-5.459326	H	4.767960	1.785848	3.006968

H	4.759458	3.263209	2.037736	H	-1.317200	-0.235180	-6.976149
H	3.634374	2.009101	0.022319	H	0.069329	1.290069	-7.542634
H	3.848684	0.548189	1.008414	H	1.491965	1.469475	-6.495853
H	2.260190	0.946307	0.337701	H	0.210356	2.666057	-6.429047
H	3.392958	4.455719	0.701652	H	-4.143113	-0.334223	-3.859103
H	1.915684	6.375993	0.218689	H	-5.859446	-1.913652	-4.827369
H	-0.426763	6.357175	1.011458	H	-6.554668	-0.375395	-4.282295
H	-1.932007	3.536531	2.967600	H	-6.760129	-1.836357	-3.308387
H	-3.018850	5.602450	3.753970	H	-5.684014	-0.669721	-1.232732
H	-1.311419	5.609163	4.229824	H	-5.651518	0.794978	-2.231886
H	-1.855384	6.599824	2.868784	H	-4.174300	0.229060	-1.433544
H	-2.607422	3.657524	0.563483	H	-5.603137	-3.096346	-1.788004
H	-3.763076	4.509246	1.605809	H	-4.379046	-5.162170	-1.207378
H	-2.593220	5.424384	0.642848	H	-2.037494	-5.455146	-1.944583
H	-3.653692	1.032183	3.429802	H	-0.172477	-2.906351	-3.969539
H	-6.465243	-0.070888	3.959148	H	0.679200	-5.117933	-4.635365
H	-5.172356	0.038544	5.161296	H	-1.015108	-4.958425	-5.132422
H	-5.844295	1.513751	4.443684	H	-0.595116	-5.932828	-3.717104
H	-5.402148	2.099470	2.030312	H	0.464834	-2.979081	-1.551003
H	-4.334237	1.101435	1.026114	H	1.525659	-4.003322	-2.535867
H	-5.944401	0.513942	1.459114	H	0.252525	-4.733720	-1.545409
H	-5.968551	-1.716160	2.357393	H	1.927801	-0.391496	-4.328945
H	-5.235248	-4.044054	1.964178	H	4.820749	0.564978	-4.672006
H	-2.860954	-4.654342	2.268355	H	3.592423	0.562399	-5.944739
H	-0.233673	-2.250475	3.434724	H	4.139371	-0.967489	-5.234706
H	-1.096392	-5.189423	3.593645	H	3.531769	-1.589223	-2.866371
H	0.416812	-4.489700	4.180480	H	2.467022	-0.557264	-1.892198
H	-1.120771	-4.057820	4.951955	H	4.128209	-0.051415	-2.221508
H	-0.107260	-2.604126	0.976243	H	4.331435	2.214590	-3.108296
H	0.937515	-3.751664	1.829412	H	3.697401	4.566924	-2.685410
H	-0.623886	-4.282954	1.176647	H	1.362653	5.294130	-3.035964
H	-2.461378	-3.169621	-5.764765	H	-1.335572	3.044240	-4.338792
H	-2.558400	-1.978866	-7.079757	H	-0.336603	5.942992	-4.390141
H	-3.788803	-2.022100	-5.802713	H	-1.859951	5.328417	-5.042591

H	-0.319028	4.845570	-5.776874
H	-1.528510	3.337357	-1.876722
H	-2.487239	4.557926	-2.728569
H	-0.923990	4.991579	-2.008865
H	5.330726	-4.718570	2.922350
H	4.620298	-3.176114	3.421632
H	6.869155	-3.504902	1.343182
H	8.225969	-2.117825	2.916701
H	6.937989	-2.182900	4.109724
H	7.217533	-4.663206	4.296951
H	8.442466	-4.630445	3.037143
H	8.655032	-3.193546	5.736513
H	9.885892	-3.175238	4.484488
H	8.908882	-5.695943	5.960843
H	10.447583	-4.866388	6.241414
H	10.147302	-5.679613	4.697684
H	4.405226	-3.758365	1.763305
H	0.169145	-0.427881	-0.371359

184

[D]

C	2.206047	2.655963	-3.102787
C	2.434382	1.285276	-3.378574
C	3.765266	0.799854	-3.476612
C	4.826838	1.686091	-3.276163
C	4.607292	3.025528	-2.975008
C	3.303688	3.497657	-2.892357
N	1.355978	0.359127	-3.499564
C	0.798912	0.146450	-4.690183
C	1.114726	1.082571	-5.843884
C	4.059590	-0.646300	-3.848181
C	4.456827	-0.770929	-5.328133
C	0.807458	3.249743	-3.042984
C	0.642386	4.448628	-3.989910
C	-0.094065	-0.900013	-5.006231
C	-0.492767	-2.069166	-4.314890
C	-1.215238	-3.083187	-5.187036
N	-0.290390	-2.313010	-3.025303
C	-0.604563	-3.588264	-2.471499
C	-1.925082	-3.926329	-2.082436
C	-2.154988	-5.170281	-1.484522
C	-1.125405	-6.076144	-1.268184
C	0.168554	-5.738739	-1.649527
C	0.454140	-4.507568	-2.244946
C	-3.109051	-2.991694	-2.285183
C	-4.241098	-3.650166	-3.089206
C	1.873338	-4.196822	-2.696508
C	2.117355	-4.706108	-4.126027
C	-3.649441	-2.478948	-0.942711
C	2.948593	-4.742203	-1.749829
C	5.138269	-1.281126	-2.962433
C	0.425375	3.651265	-1.613097
Ca	0.323554	-0.539985	-1.548333
C	-1.410226	0.733084	-0.106712
C	-2.029289	0.848862	-1.508351
C	-3.407776	1.516166	-1.582845
C	-3.977289	1.625296	-2.996634
C	-5.357476	2.281721	-3.047929
C	-5.920550	2.389788	-4.463217
Ca	0.054211	0.321896	1.939806
N	0.240569	2.375550	3.176757
C	0.624812	3.623324	2.600881
C	1.980741	3.915234	2.313774
C	2.302986	5.141433	1.722379
C	1.324809	6.078497	1.417048
C	-0.006425	5.781699	1.687322
C	-0.382150	4.565715	2.265115
C	3.104006	2.934303	2.611805
C	4.241755	3.561422	3.431265

C	-1.850502	4.291608	2.554508	H	-2.689668	-0.038295	6.418639
C	-2.275253	4.887066	3.906497	H	-2.073185	-1.604131	5.899509
C	0.269347	2.235716	4.499479	H	2.681412	2.117745	3.207505
C	0.915910	3.309262	5.360857	H	3.869110	4.046595	4.339611
C	-0.248999	1.138013	5.228157	H	4.965792	2.792395	3.726948
C	-1.159839	0.105234	4.900313	H	4.787503	4.317210	2.854005
C	-1.783386	-0.572360	6.109536	H	4.061309	3.110339	0.655255
N	-1.515996	-0.259139	3.672069	H	4.476903	1.628888	1.532411
C	-2.643041	-1.119196	3.508455	H	2.893493	1.793621	0.747876
C	-3.952988	-0.572893	3.505001	H	3.344135	5.367013	1.503107
C	-5.042248	-1.419862	3.281126	H	1.595691	7.031562	0.969053
C	-4.875063	-2.783071	3.070736	H	-0.772063	6.512884	1.442310
C	-3.592178	-3.314728	3.083278	H	-1.966360	3.203776	2.624483
C	-2.468139	-2.510051	3.297741	H	-3.336459	4.686681	4.100012
C	-4.221967	0.907986	3.730456	H	-1.699208	4.462768	4.734601
C	-5.141214	1.150864	4.937703	H	-2.130544	5.974840	3.915718
C	-1.100939	-3.175740	3.336414	H	-2.468617	4.423858	0.460886
C	-0.970815	-4.150812	4.517306	H	-3.804619	4.447327	1.625130
C	-0.777763	-3.896752	2.021231	H	-2.815893	5.888537	1.396985
C	-4.817295	1.559947	2.474381	H	-3.264476	1.397264	3.934468
C	3.662862	2.331145	1.314921	H	-6.149965	0.758324	4.761002
C	-2.781245	4.793202	1.443511	H	-4.757600	0.673124	5.845483
C	2.144601	-0.948376	2.912419	H	-5.236522	2.225610	5.134581
C	2.268697	-1.623749	1.736234	H	-5.012844	2.624718	2.649968
C	3.528902	-1.596272	0.911479	H	-4.137299	1.473368	1.620803
C	4.575636	-2.486410	1.600943	H	-5.768806	1.090397	2.195947
C	5.917009	-2.553937	0.876209	H	-6.045767	-0.999951	3.275546
C	6.939595	-3.413656	1.616702	H	-5.736498	-3.425149	2.903833
H	1.221547	-0.805518	0.335342	H	-3.456463	-4.383384	2.931851
H	2.007376	3.203842	5.338683	H	-0.355404	-2.387715	3.487351
H	0.686818	4.312422	4.991695	H	-1.679517	-4.982284	4.423102
H	0.593722	3.234408	6.402102	H	0.039629	-4.576283	4.554380
H	-0.046149	1.201317	6.292431	H	-1.163962	-3.656323	5.474913
H	-1.092602	-0.560170	6.957455	H	-0.814346	-3.217861	1.161910

H	0.221323	-4.347435	2.060252
H	-1.489532	-4.704324	1.819688
H	-1.118084	-4.100323	-4.800986
H	-0.837122	-3.056016	-6.212555
H	-2.285899	-2.847201	-5.226456
H	-0.442475	-0.874276	-6.033907
H	0.858386	0.635819	-6.807382
H	2.169730	1.368043	-5.856222
H	0.537602	2.009549	-5.739096
H	-2.759427	-2.125878	-2.858192
H	-3.882731	-4.057104	-4.040466
H	-5.028967	-2.918254	-3.304631
H	-4.703815	-4.472933	-2.531494
H	-4.025323	-3.302124	-0.324500
H	-4.476067	-1.775798	-1.099877
H	-2.877228	-1.965085	-0.361223
H	-3.166844	-5.432620	-1.184350
H	-1.328142	-7.039850	-0.807380
H	0.972236	-6.451225	-1.484795
H	1.973338	-3.104698	-2.725616
H	3.136465	-4.467471	-4.454876
H	1.420227	-4.253213	-4.837508
H	1.991535	-5.794969	-4.175242
H	2.757203	-4.454732	-0.710083
H	3.932647	-4.353563	-2.035501
H	3.014504	-5.836131	-1.788068
H	3.131914	-1.212231	-3.708892
H	5.367734	-0.196057	-5.537329
H	3.668836	-0.403150	-5.992514
H	4.652524	-1.818714	-5.588012
H	5.233144	-2.349954	-3.189125
H	4.899534	-1.175793	-1.899496
H	6.124042	-0.830101	-3.128178
H	5.847603	1.321434	-3.361993
H	5.447019	3.697954	-2.816771
H	3.130048	4.547932	-2.669516
H	0.107398	2.474483	-3.374456
H	1.247338	5.303743	-3.666413
H	-0.403902	4.777149	-4.007371
H	0.940609	4.201771	-5.014648
H	0.475201	2.800874	-0.924239
H	-0.597059	4.046226	-1.580176
H	1.094267	4.425590	-1.222466
H	1.446099	-1.259662	3.687706
H	2.852492	-0.168075	3.186338
H	1.636990	-2.495520	1.576932
H	3.333477	-1.954897	-0.105487
H	3.912652	-0.573562	0.825127
H	4.730800	-2.114154	2.621956
H	4.169783	-3.503276	1.711204
H	6.314269	-1.536288	0.751538
H	5.769500	-2.950120	-0.137751
H	7.135458	-3.018900	2.621358
H	7.895247	-3.450907	1.081319
H	6.581443	-4.444245	1.731356
H	-2.164173	0.217481	0.523401
H	-1.379234	1.765413	0.295896
H	-2.143024	-0.148385	-1.978411
H	-1.360126	1.415013	-2.182686
H	-4.102026	0.950208	-0.945810
H	-3.333100	2.517443	-1.135526
H	-3.281110	2.197544	-3.628771
H	-4.036934	0.622399	-3.446400
H	-5.298093	3.283538	-2.598797
H	-6.053621	1.708825	-2.418582
H	-5.261537	2.985332	-5.107317
H	-6.908403	2.864931	-4.468244
H	-6.025097	1.399868	-4.924628

184

[TS-DE]

C	24.456856	21.899686	3.145105
C	24.685240	20.528724	2.871514
C	26.015761	20.042877	2.771150
C	27.077485	20.929506	2.970665
C	26.858062	22.268860	3.271877
C	25.554712	22.741468	3.354537
N	23.603932	19.601922	2.752542
C	23.049385	19.389866	1.559029
C	23.366860	20.326015	0.405941
C	26.310992	18.596983	2.399029
C	26.708167	18.473215	0.918914
C	23.058492	22.493903	3.204403
C	22.893750	23.692665	2.257301
C	22.157375	18.343961	1.241497
C	21.758789	17.175866	1.933788
C	21.037181	16.160783	1.062533
N	21.960755	16.935234	3.225096
C	21.647020	15.656743	3.778158
C	20.326262	15.318257	4.165202
C	20.096358	14.073820	4.762204
C	21.126181	13.168312	4.978456
C	22.420030	13.505627	4.597101
C	22.705842	14.737315	4.002403
C	19.142043	16.252432	3.962218
C	18.010207	15.593570	3.158168
C	24.125325	15.046997	3.550783
C	24.368626	14.537681	2.121112
C	18.601570	16.764908	5.304722
C	25.200247	14.501370	4.497619
C	27.390162	17.962612	3.284502
C	22.676921	22.895768	4.634247
Ca	22.585805	18.695218	4.701758
C	20.860720	19.963183	6.132816
C	20.227271	20.088505	4.735114
C	18.847345	20.758239	4.667573
C	18.275868	20.868654	3.252999
C	16.895047	21.525169	3.200767
C	16.331936	21.633135	1.785075
Ca	22.314316	19.563285	8.215377
N	22.487817	21.623030	9.424399
C	22.875342	22.867926	8.848467
C	24.231804	23.158920	8.561085
C	24.554036	24.385149	7.969482
C	23.576288	25.322726	7.663877
C	22.244776	25.026419	7.933762
C	21.869031	23.810707	8.511690
C	25.354556	22.177348	8.859385
C	26.492733	22.804565	9.678314
C	20.400939	23.536042	8.801614
C	19.976143	24.131041	10.153717
C	22.519473	21.480168	10.746788
C	23.168583	22.552272	11.607902
C	21.998780	20.384403	11.475164
C	21.090881	19.349916	11.147273
C	20.469270	18.670550	12.356496
N	20.730828	18.987351	9.919686
C	19.607267	18.125177	9.756003
C	18.297020	18.670587	9.751997
C	17.208038	17.823598	9.528051
C	17.376017	16.460386	9.317782
C	18.659294	15.929394	9.330426
C	19.783360	16.734207	9.545101
C	18.028909	20.151513	9.977752
C	17.109953	20.394784	11.184991
C	21.151496	16.070014	9.583977
C	21.280875	15.093628	10.764087

C	21.473090	15.347789	8.268894
C	17.433787	20.803928	8.721880
C	25.914096	21.576061	7.562109
C	19.469786	24.037099	7.690798
C	24.355895	18.323125	9.124794
C	24.461654	17.659471	7.889467
C	25.784811	17.643759	7.139781
C	26.817826	16.759833	7.851389
C	28.164349	16.690542	7.129817
C	29.190226	15.830292	7.865516
H	23.617042	18.262148	6.773990
H	24.259798	22.444972	11.584941
H	22.940302	23.556119	11.239811
H	22.847106	22.477178	12.649377
H	22.205272	20.445085	12.538873
H	21.160620	18.683073	13.203902
H	19.562263	19.203039	12.666440
H	20.181113	17.638329	12.146333
H	24.932933	21.357835	9.451079
H	26.120123	23.289302	10.586907
H	27.216175	22.035085	9.973962
H	27.038683	23.560785	9.101526
H	26.315109	22.354783	6.903213
H	26.723480	20.868815	7.778973
H	25.142195	21.042367	6.994293
H	25.595424	24.610711	7.751051
H	23.847688	26.276080	7.216676
H	21.479466	25.758207	7.689192
H	20.285957	22.448179	8.872463
H	18.915071	23.930157	10.347638
H	20.552811	23.706377	10.981114
H	20.120603	25.218860	10.163234
H	19.782556	23.668279	6.707848
H	18.446383	23.691146	7.872373
H	19.434944	25.132416	7.643919
H	18.986735	20.639916	10.181846
H	16.101020	20.002444	11.008648
H	17.494036	19.916751	12.092352
H	17.015111	21.469532	11.382215
H	17.238289	21.868746	8.897286
H	18.113919	20.717731	7.868292
H	16.482406	20.334350	8.442927
H	16.204224	18.242984	9.522976
H	16.514790	15.817749	9.151592
H	18.795367	14.860615	9.179764
H	21.897614	16.857769	9.733591
H	20.572037	14.262099	10.669817
H	22.291447	14.668540	10.801039
H	21.088293	15.587813	11.721938
H	21.433873	16.026589	7.409232
H	22.473342	14.899993	8.305836
H	20.761942	14.539076	8.068842
H	21.134647	15.143808	1.448660
H	21.415272	16.188205	0.037080
H	19.966493	16.396618	1.023301
H	21.810102	18.369066	0.213538
H	23.110489	19.879155	-0.557414
H	24.421885	20.611085	0.393736
H	22.789965	21.253128	0.510637
H	19.491096	17.118312	3.388841
H	18.368565	15.186226	2.207076
H	17.222425	16.325443	2.942494
H	17.547377	14.771121	3.716110
H	18.226262	15.941892	5.923294
H	17.774608	17.467660	5.147735
H	19.373459	17.278553	5.886877
H	19.084650	13.811283	5.062370
H	20.923685	12.204837	5.439693

H	23.223764	12.793388	4.761911
H	24.227092	16.139138	3.520985
H	25.387864	14.775742	1.792482
H	23.671743	14.990560	1.409306
H	24.242433	13.448913	2.072139
H	25.009562	14.788779	5.537369
H	26.184233	14.890039	4.212123
H	25.265923	13.407513	4.458621
H	25.383907	18.029701	2.537482
H	27.618999	19.048210	0.710059
H	25.920352	18.840839	0.254183
H	26.904248	17.425554	0.659127
H	27.485052	16.893883	3.057698
H	27.152790	18.067382	4.347664
H	28.375654	18.413704	3.117733
H	28.098082	20.564743	2.884730
H	27.697872	22.940994	3.430425
H	25.381347	23.791734	3.577176
H	22.358064	21.718978	2.872757
H	23.498726	24.547682	2.580730
H	21.847543	24.021295	2.239964
H	23.191837	23.445734	1.232551
H	22.729194	22.045918	5.323796
H	21.654052	23.289308	4.668329
H	23.344935	23.670714	5.024881
H	23.816880	17.866116	9.952294
H	25.099937	19.070571	9.396781
H	23.944337	16.698704	7.832975
H	25.632085	17.280934	6.113215
H	26.175127	18.665770	7.058184
H	26.963791	17.136339	8.871769
H	26.411377	15.742832	7.960004
H	28.562297	17.708120	7.005261
H	28.017379	16.295517	6.114922
H	29.388517	16.223242	8.870346
H	30.144528	15.794716	7.327293
H	28.833307	14.799160	7.979528
H	20.114927	19.446469	6.769453
H	20.906667	20.991700	6.540091
H	20.109743	19.093225	4.262191
H	20.893299	20.656742	4.059898
H	18.153794	20.191684	5.304585
H	18.923446	21.758969	5.115551
H	18.971874	21.441038	2.620850
H	18.216042	19.865860	2.803061
H	16.954026	22.527074	3.649586
H	16.198665	20.952353	3.829814
H	16.990501	22.228940	1.140791
H	15.344165	22.108309	1.780876
H	16.226896	20.643328	1.323571

184

[E]

C	2.057307	2.899381	-3.099994
C	2.366577	1.531067	-3.301168
C	3.724322	1.114805	-3.335124
C	4.729993	2.067207	-3.149718
C	4.430045	3.407028	-2.929257
C	3.101737	3.810179	-2.906809
N	1.340456	0.544388	-3.414740
C	0.773819	0.314301	-4.596754
C	1.048998	1.241322	-5.770690
C	4.099183	-0.329898	-3.631561
C	4.321738	-0.544733	-5.137694
C	0.628183	3.420266	-3.097722
C	0.425609	4.572444	-4.093916
C	-0.111269	-0.745667	-4.897460
C	-0.457510	-1.938853	-4.223261
C	-1.232426	-2.925991	-5.082943

N	-0.174067	-2.226308	-2.956120	C	0.472123	2.215698	4.474240
C	-0.390450	-3.544151	-2.452166	C	1.211694	3.259755	5.293000
C	-1.650826	-3.945161	-1.944319	C	-0.024197	1.128642	5.225487
C	-1.778516	-5.215892	-1.371914	C	-0.939404	0.094090	4.925722
C	-0.705352	-6.093702	-1.306165	C	-1.492413	-0.610255	6.152956
C	0.524041	-5.704919	-1.827448	N	-1.367315	-0.238323	3.712224
C	0.707866	-4.444322	-2.401588	C	-2.421182	-1.193044	3.582966
C	-2.880848	-3.053162	-2.016136	C	-3.769422	-0.753235	3.568997
C	-4.047841	-3.735524	-2.746938	C	-4.786625	-1.693690	3.381944
C	2.050537	-4.081632	-3.019815	C	-4.506064	-3.045556	3.223160
C	2.103219	-4.493948	-4.500304	C	-3.183617	-3.470884	3.243155
C	-3.329830	-2.593421	-0.623333	C	-2.127109	-2.570261	3.414897
C	3.251988	-4.677959	-2.277116	C	-4.147969	0.707242	3.770392
C	5.326640	-0.814972	-2.850705	C	-5.020871	0.902886	5.019729
C	0.189049	3.853310	-1.693440	C	-0.704343	-3.111470	3.439919
Ca	0.340219	-0.421394	-1.450484	C	-0.455331	-4.025863	4.650209
C	-1.679004	0.868821	-0.359682	C	-0.357754	-3.857497	2.144235
C	-2.261136	0.947308	-1.779619	C	-4.852596	1.288014	2.536004
C	-3.661495	1.566959	-1.901111	C	3.640153	2.386445	0.884442
C	-4.196999	1.624476	-3.332537	C	-2.739626	4.729931	1.764156
C	-5.592185	2.242773	-3.441742	C	1.568191	-0.763336	0.839804
C	-6.118160	2.295368	-4.874752	C	2.602309	-1.534147	0.007350
Ca	-0.327987	0.589443	1.729002	C	3.894303	-1.967231	0.727424
N	0.340871	2.379699	3.158724	C	3.688529	-2.969973	1.861496
C	0.714436	3.630809	2.579636	C	4.991624	-3.378297	2.551518
C	2.039129	3.876371	2.138572	C	4.784938	-4.390650	3.676389
C	2.339066	5.111763	1.554317	H	2.928360	-0.928915	-0.860076
C	1.373749	6.098456	1.399434	H	2.293444	3.151687	5.153275
C	0.072732	5.846553	1.819399	H	0.952288	4.274686	4.980129
C	-0.282134	4.626561	2.401671	H	0.999460	3.154892	6.359385
C	3.152154	2.846613	2.265823	H	0.232900	1.179594	6.278327
C	4.345534	3.365272	3.083926	H	-0.752811	-0.622939	6.958374
C	-1.717242	4.418012	2.865510	H	-2.371905	-0.070751	6.525602
C	-2.023830	5.241627	4.126309	H	-1.808182	-1.633376	5.942526

H	2.741734	1.975503	2.790520	H	-1.131103	-3.952425	-4.724809
H	4.042542	3.734233	4.069176	H	-0.907034	-2.880755	-6.125877
H	5.081382	2.565600	3.232161	H	-2.300169	-2.673167	-5.062611
H	4.854972	4.188170	2.568194	H	-0.498634	-0.703243	-5.910944
H	4.092790	3.213228	0.326101	H	0.942151	0.715747	-6.723064
H	4.395542	1.597552	0.982888	H	2.047498	1.681314	-5.719614
H	2.821655	1.995559	0.271530	H	0.329321	2.069629	-5.768500
H	3.355486	5.304337	1.218630	H	-2.610757	-2.161281	-2.591140
H	1.632061	7.055983	0.953562	H	-3.750649	-4.099364	-3.736089
H	-0.684360	6.617833	1.698109	H	-4.879147	-3.031793	-2.876355
H	-1.824453	3.360572	3.131685	H	-4.429762	-4.592352	-2.179571
H	-3.050811	5.059203	4.466173	H	-3.625266	-3.443038	0.002062
H	-1.347537	4.987061	4.948934	H	-4.188897	-1.915772	-0.694800
H	-1.921381	6.316034	3.929086	H	-2.530628	-2.064956	-0.092992
H	-2.550707	4.140284	0.860409	H	-2.744198	-5.523079	-0.976674
H	-3.753963	4.500182	2.110805	H	-0.825891	-7.078196	-0.860624
H	-2.724811	5.788976	1.480556	H	1.358246	-6.400096	-1.789697
H	-3.221684	1.271652	3.922641	H	2.137513	-2.989186	-2.984337
H	-5.993354	0.407464	4.911050	H	3.070920	-4.224068	-4.941028
H	-4.541869	0.494766	5.915966	H	1.320570	-4.004003	-5.086099
H	-5.210371	1.969498	5.191509	H	1.974604	-5.578726	-4.603573
H	-5.101090	2.344052	2.696487	H	3.210435	-4.477358	-1.201391
H	-4.223356	1.220926	1.642025	H	4.182410	-4.249162	-2.666876
H	-5.788598	0.757224	2.323411	H	3.320559	-5.764087	-2.414048
H	-5.822028	-1.360064	3.371270	H	3.244367	-0.951005	-3.339252
H	-5.312522	-3.762377	3.088893	H	5.137447	0.091565	-5.503539
H	-2.962489	-4.529509	3.126611	H	3.424493	-0.309634	-5.717556
H	-0.024120	-2.256872	3.532770	H	4.589450	-1.588738	-5.342723
H	-1.096775	-4.914859	4.614443	H	5.448157	-1.896935	-2.978188
H	0.586600	-4.368086	4.660579	H	5.237697	-0.606723	-1.779296
H	-0.649810	-3.513109	5.597657	H	6.251984	-0.346737	-3.207689
H	-0.509873	-3.231717	1.258668	H	5.770513	1.755651	-3.181896
H	0.689826	-4.178428	2.154316	H	5.227838	4.131541	-2.784274
H	-0.976660	-4.752694	2.017626	H	2.865367	4.858880	-2.742239

H	-0.023348	2.598462	-3.412699
H	0.968864	5.473296	-3.785410
H	-0.636726	4.838048	-4.155985
H	0.771054	4.305881	-5.098496
H	0.256523	3.026313	-0.978330
H	-0.850401	4.203217	-1.701280
H	0.815096	4.666215	-1.310246
H	1.224696	-1.455193	1.635524
H	2.136192	0.018064	1.388286
H	2.137022	-2.439803	-0.419636
H	4.589354	-2.400713	-0.007133
H	4.387030	-1.067484	1.125340
H	3.002837	-2.546907	2.608536
H	3.193073	-3.869565	1.464363
H	5.484180	-2.480747	2.952717
H	5.682967	-3.795445	1.805083
H	4.127037	-3.986985	4.456156
H	5.734289	-4.663982	4.151426
H	4.323860	-5.312359	3.299880
H	-2.436395	0.334775	0.255202
H	-1.697719	1.910134	0.031522
H	-2.311818	-0.058622	-2.232390
H	-1.586719	1.524308	-2.433528
H	-4.356264	0.993163	-1.270205
H	-3.634464	2.581634	-1.476960
H	-3.498171	2.197421	-3.960964
H	-4.217901	0.608434	-3.755305
H	-5.571326	3.258464	-3.021266
H	-6.291321	1.670154	-2.815392
H	-5.458579	2.891964	-5.517295
H	-7.118612	2.741050	-4.920203
H	-6.181227	1.290366	-5.310466

184

[F]

Ca	19.228654	12.998280	8.885079
N	17.229352	14.232036	9.294274
N	17.754398	11.114680	8.996304
C	15.186891	14.608790	10.615289
H	14.759721	15.263017	9.849170
H	14.378823	14.036123	11.075575
H	15.620681	15.264657	11.378782
C	16.251820	13.701097	10.025687
C	16.086036	12.326908	10.307898
H	15.248645	12.121151	10.965524
C	16.671975	11.157366	9.768427
C	15.956422	9.869044	10.138741
H	16.496832	9.353269	10.941359
H	14.940886	10.064384	10.490856
H	15.909270	9.177964	9.292810
C	17.137210	15.600498	8.895073
C	17.677716	16.636757	9.696570
C	18.302270	16.367410	11.057087
H	18.094547	15.322086	11.312944
C	17.696500	17.238884	12.167924
H	16.604301	17.159657	12.198209
H	18.087746	16.933269	13.145825
H	17.946841	18.297708	12.033002
C	19.825376	16.552594	11.016305
H	20.093899	17.583143	10.759419
H	20.273878	16.321120	11.990058
H	20.295276	15.902111	10.269772
C	17.639188	17.952522	9.223713
H	18.060234	18.747081	9.835294
C	17.068563	18.267022	7.996795
H	17.040946	19.297343	7.650414
C	16.525353	17.249467	7.221676
H	16.065860	17.495246	6.266822
C	16.549022	15.916806	7.642784

C	15.906603	14.856694	6.759009
H	16.108221	13.881156	7.213857
C	14.380766	15.030919	6.693963
H	14.109948	15.992115	6.239658
H	13.928623	14.235946	6.088184
H	13.925357	14.993647	7.689215
C	16.499430	14.844717	5.343655
H	17.579604	14.666374	5.362726
H	16.036034	14.052728	4.743384
H	16.325433	15.793291	4.821429
C	18.113753	9.886071	8.362366
C	17.718539	9.673111	7.015144
C	16.856211	10.682594	6.270892
H	16.967765	11.645566	6.782883
C	15.368584	10.302450	6.342013
H	15.012800	10.260184	7.376063
H	14.755692	11.037870	5.806206
H	15.194699	9.320322	5.884594
C	17.283411	10.875459	4.810651
H	17.086619	9.984258	4.202704
H	16.721753	11.701405	4.360316
H	18.350489	11.108872	4.730392
C	18.095273	8.487972	6.377467
H	17.781604	8.312919	5.351816
C	18.860059	7.525344	7.026459
H	19.142183	6.609427	6.512795
C	19.259500	7.747706	8.337595
H	19.863515	6.998960	8.844806
C	18.897784	8.910316	9.026865
C	19.379680	9.082860	10.459498
H	18.890676	9.973557	10.869502
C	19.005124	7.894691	11.358729
H	19.540592	6.983979	11.065729
H	19.271747	8.108465	12.400812

H	17.933326	7.672886	11.319541
C	20.896705	9.314395	10.504124
H	21.194208	10.177058	9.898993
H	21.235040	9.490725	11.532356
H	21.441246	8.446263	10.115926
C	21.364164	13.134431	10.369903
H	21.935147	14.039546	10.666760
H	22.105238	12.311659	10.439122
C	20.339937	12.893312	11.491500
H	19.597479	13.710980	11.514188
H	19.756825	11.973959	11.295398
C	20.903956	12.762011	12.914096
H	21.483988	13.667326	13.144783
H	21.621564	11.929025	12.931742
C	19.837401	12.547316	13.989185
H	19.253712	11.645209	13.750337
H	19.122222	13.383683	13.964526
C	20.407528	12.416644	15.402794
H	21.116995	11.577135	15.430643
H	20.993287	13.316156	15.640980
C	19.332594	12.211549	16.468431
H	18.752868	11.300207	16.275867
H	19.770191	12.122300	17.469480
H	18.628121	13.052414	16.486557
Ca	22.472553	13.625481	8.153301
N	24.471996	12.391944	7.744113
N	23.946614	15.509236	8.042066
C	26.514520	12.015386	6.423119
H	26.941313	11.360699	7.189044
H	27.322848	12.588141	5.963389
H	26.080866	11.360022	5.659102
C	25.449521	12.922973	7.012760
C	25.615209	14.297184	6.730607
H	26.452630	14.503040	6.073048

C	25.029116	15.466659	7.270049
C	25.744604	16.755049	6.899847
H	25.204160	17.270905	6.097306
H	26.760141	16.559785	6.547689
H	25.791757	17.446030	7.745858
C	24.564218	11.023500	8.143358
C	24.023841	9.987158	7.341880
C	23.399431	10.256352	5.981263
H	23.607004	11.301708	5.725414
C	24.005560	9.384960	4.870555
H	25.097746	9.464410	4.840448
H	23.614408	9.690452	3.892578
H	23.755422	8.326088	5.005487
C	21.876351	10.070878	6.021775
H	21.607990	9.040286	6.278656
H	21.427993	10.302212	5.047922
H	21.406172	10.721297	6.768188
C	24.062423	8.671424	7.814824
H	23.641491	7.876797	7.203251
C	24.632980	8.357035	9.041801
H	24.660639	7.326737	9.388246
C	25.176086	9.374670	9.816890
H	25.635535	9.128979	10.771787
C	25.152361	10.707303	9.395697
C	25.794688	11.767514	10.279420
H	25.592989	12.743011	9.824520
C	27.320540	11.593421	10.344454
H	27.591445	10.632247	10.798754
H	27.772619	12.388434	10.950228
H	27.775926	11.630731	9.349193
C	25.201867	11.779515	11.694776
H	24.121673	11.957738	11.675699
H	25.665183	12.571593	12.294993
H	25.375973	10.830993	12.217060

C	23.587048	16.737823	8.675920
C	23.981957	16.950803	10.023230
C	24.844093	15.941300	10.767676
H	24.732349	14.978254	10.255858
C	26.331798	16.321112	10.696469
H	26.687599	16.363043	9.662413
H	26.944530	15.585695	11.232466
H	26.505875	17.303316	11.153653
C	24.416880	15.748782	12.227958
H	24.614016	16.639987	12.835791
H	24.978259	14.922692	12.678375
H	23.349720	15.515764	12.308293
C	23.605075	18.135950	10.660807
H	23.918508	18.311020	11.686525
C	22.840408	19.098552	10.011633
H	22.558168	20.014479	10.525216
C	22.441216	18.876142	8.700428
H	21.837265	19.624851	8.193089
C	22.803096	17.713528	8.011254
C	22.321420	17.540909	6.578554
H	22.810565	16.650253	6.168629
C	22.695961	18.729105	5.679352
H	22.160336	19.639755	5.972255
H	22.429514	18.515276	4.637235
H	23.767725	18.951049	5.718685
C	20.804423	17.309223	6.533734
H	20.506925	16.446532	7.138829
H	20.466237	17.132860	5.505458
H	20.259748	18.177301	6.921865
C	20.337083	13.489115	6.668462
H	19.766183	12.583926	6.371686
H	19.595944	14.311818	6.599141
C	21.361320	13.730200	5.546870
H	22.103832	12.912570	5.524287

H	21.944366	14.649612	5.742898
C	20.797339	13.861322	4.124244
H	20.217327	12.955970	3.893648
H	20.079723	14.694299	4.106475
C	21.863922	14.075898	3.049159
H	22.447622	14.978018	3.287930
H	22.579084	13.239519	3.073913
C	21.293831	14.206439	1.635523
H	20.584405	15.045979	1.607564
H	20.708034	13.306928	1.397426
C	22.368798	14.411361	0.569887
H	22.948566	15.322695	0.762361
H	21.931227	14.500521	-0.431181
H	23.073230	13.570459	0.551869

186

[F1] scf done: -4306.903730 / Energies= -4305.158315 / Enthalpies= -4305.157371 / Free Energies= -4305.393277

C	1.973309	4.359591	-0.301677
C	2.816718	3.364311	0.251387
C	3.316387	3.520670	1.571356
C	2.951688	4.652992	2.305206
C	2.111411	5.623947	1.772683
C	1.631389	5.469485	0.478166
N	3.140035	2.176474	-0.472624
C	4.220638	2.174509	-1.254078
C	4.919956	3.486082	-1.568117
C	4.282986	2.519201	2.189281
C	3.822488	2.027109	3.567679
C	1.432359	4.268234	-1.720223
C	-0.081687	4.020198	-1.727175
C	4.813388	1.037205	-1.836889
C	4.624911	-0.352166	-1.643389
C	5.746547	-1.196620	-2.229248
N	3.600806	-0.916803	-1.017070
C	3.582319	-2.321955	-0.774281
C	3.206922	-3.249858	-1.778880
C	3.102305	-4.603648	-1.441136
C	3.360370	-5.056073	-0.154414
C	3.744273	-4.144340	0.823143
C	3.864261	-2.781300	0.540334
C	2.917314	-2.842345	-3.218052
C	1.456753	-3.112641	-3.609489
C	4.359599	-1.823788	1.615144
C	3.938922	-2.213232	3.036395
C	3.852634	-3.550334	-4.212303
C	5.888220	-1.672572	1.543954
C	5.702528	3.101556	2.283257
C	1.763498	5.513685	-2.556923
Ca	1.621363	0.330096	-0.397660
C	0.499610	0.038989	1.864053
C	-0.528579	-0.167934	2.990237
C	0.017892	-0.157606	4.425411
C	-1.050857	-0.359597	5.500776
C	-0.497183	-0.344910	6.926811
C	-1.573570	-0.539759	7.992851
C	-0.536319	0.213301	-1.842960
C	0.482674	0.522159	-2.954145
C	-0.080133	0.663240	-4.376100
C	0.980954	0.940614	-5.442231
C	0.408325	1.093281	-6.852602
C	1.478009	1.351293	-7.911978
Ca	-1.631901	-0.238102	0.406520
N	-3.654897	0.986277	0.777407
C	-3.741207	2.333255	0.310709
C	-3.242329	3.413754	1.080267
C	-3.277390	4.704115	0.540991
C	-3.805104	4.950838	-0.720142

C	-4.311411	3.890535	-1.463017
C	-4.290397	2.580973	-0.975200
C	-2.680572	3.222613	2.480833
C	-1.167310	3.475317	2.521210
C	-4.909504	1.470379	-1.813150
C	-4.338438	1.415354	-3.236150
C	-4.656425	0.486820	1.501064
C	-4.833518	-0.870585	1.837572
C	-4.214139	-2.062018	1.391764
C	-4.967090	-3.321461	1.791966
C	-5.736757	1.416429	2.027086
N	-3.090499	-2.137379	0.688674
C	-2.644849	-3.391085	0.173975
C	-2.861092	-3.672037	-1.201234
C	-2.350240	-4.855883	-1.740149
C	-1.635463	-5.757991	-0.960101
C	-1.433307	-5.481024	0.385215
C	-1.927327	-4.313126	0.976286
C	-3.691527	-2.739921	-2.071835
C	-3.216308	-2.668323	-3.527262
C	-1.669630	-4.091107	2.460569
C	-0.172204	-3.922251	2.757371
C	-3.385293	4.108881	3.519835
C	-6.440641	1.600471	-1.862087
C	-5.178466	-3.127842	-2.022501
C	-2.242026	-5.228872	3.321459
H	-6.046812	2.139894	1.268303
H	-6.615056	0.860949	2.363769
H	-5.356884	1.994617	2.877931
H	-5.705685	-1.052163	2.457788
H	-4.732438	-3.580833	2.831692
H	-6.047572	-3.161353	1.734602
H	-4.703308	-4.177230	1.167518
H	-2.858327	2.179228	2.765166
H	-4.473006	3.983543	3.493579
H	-3.036833	3.860748	4.529806
H	-3.172200	5.171333	3.352846
H	-0.930403	4.510834	2.253310
H	-0.768233	3.290654	3.525938
H	-0.623872	2.831370	1.821450
H	-2.891149	5.534019	1.128492
H	-3.830240	5.962474	-1.118007
H	-4.741457	4.084500	-2.443113
H	-4.676556	0.519936	-1.321299
H	-6.741871	2.540414	-2.341201
H	-6.878651	0.775536	-2.437575
H	-6.881400	1.581238	-0.859976
H	-3.251815	1.282036	-3.228301
H	-4.778519	0.576560	-3.789001
H	-4.560560	2.328779	-3.801013
H	-3.610317	-1.733318	-1.642884
H	-5.570291	-3.094554	-1.001534
H	-5.777265	-2.443441	-2.636163
H	-5.325056	-4.144787	-2.407604
H	-3.414876	-3.599838	-4.071078
H	-3.749113	-1.869537	-4.055197
H	-2.143056	-2.460882	-3.596158
H	-2.516553	-5.079471	-2.790299
H	-1.243345	-6.672543	-1.398177
H	-0.878207	-6.189102	0.996388
H	-2.175198	-3.164679	2.754816
H	-1.710344	-6.170888	3.141415
H	-2.136153	-4.991402	4.387127
H	-3.302366	-5.404215	3.114391
H	0.262499	-3.088585	2.197072
H	-0.010463	-3.732742	3.825656
H	0.389118	-4.824791	2.488607
H	1.016615	0.993692	2.091141

H	1.281604	-0.735489	2.012641	H	4.187341	-1.405124	3.733309
H	-1.311365	0.610677	2.942660	H	4.462074	-3.111212	3.386900
H	-1.063977	-1.127054	2.858761	H	4.327798	1.652568	1.521092
H	0.535256	0.797896	4.594157	H	6.084436	3.397841	1.300977
H	0.785922	-0.939394	4.514184	H	6.395114	2.361628	2.703230
H	-1.570729	-1.313803	5.325112	H	5.724234	3.986855	2.931014
H	-1.818320	0.423445	5.403471	H	3.793379	2.840543	4.302730
H	0.265063	-1.130971	7.027160	H	4.516474	1.268814	3.950411
H	0.024678	0.606872	7.101996	H	2.824258	1.579903	3.522362
H	-2.091099	-1.498399	7.862582	H	3.341530	4.781131	3.312606
H	-1.146967	-0.526528	9.002507	H	1.839784	6.498004	2.359704
H	-2.330323	0.252883	7.940677	H	0.981323	6.233020	0.056502
H	5.761541	-2.209495	-1.822495	H	1.917351	3.411393	-2.201495
H	6.717308	-0.727707	-2.042801	H	1.237817	6.400783	-2.184249
H	5.627918	-1.271205	-3.317124	H	1.454403	5.364734	-3.598651
H	5.679744	1.265398	-2.449859	H	2.835532	5.738291	-2.548297
H	4.327272	4.067525	-2.284379	H	-0.350166	3.119399	-1.164780
H	5.907463	3.320252	-2.004689	H	-0.453415	3.898842	-2.751778
H	5.029764	4.107290	-0.674937	H	-0.624446	4.855972	-1.272040
H	3.092909	-1.764294	-3.304405	H	-1.070170	-0.707485	-2.159111
H	4.906889	-3.411138	-3.954213	H	-1.307719	1.008202	-1.903763
H	3.694614	-3.163275	-5.226447	H	1.262902	-0.260498	-2.993624
H	3.659041	-4.629363	-4.239438	H	1.023830	1.460995	-2.732710
H	1.222304	-4.182074	-3.552202	H	-0.626615	-0.256703	-4.629061
H	1.269534	-2.784586	-4.639313	H	-0.826498	1.470739	-4.377562
H	0.754061	-2.589982	-2.954299	H	1.536922	1.853015	-5.177456
H	2.809759	-5.318160	-2.207105	H	1.721087	0.126037	-5.439137
H	3.270572	-6.112739	0.085282	H	-0.321273	1.915761	-6.859538
H	3.959609	-4.502431	1.825864	H	-0.155507	0.186239	-7.114184
H	3.933197	-0.835928	1.398982	H	2.037130	2.270057	-7.695118
H	6.378885	-2.638758	1.715589	H	1.038273	1.457248	-8.910469
H	6.243544	-0.970881	2.308707	H	2.201381	0.527399	-7.952343
H	6.211393	-1.298440	0.568192	H	0.587201	-2.818115	-0.209663
H	2.862119	-2.400650	3.107283	H	0.233450	-2.362357	-0.679232

186

[TS-F1F2] scf done: -4306.884899 / Energies= -4305.139672 / Enthalpies= -4305.138728 / Free Energies= -4305.371088

C	23.070572	17.592384	8.415191
C	23.836882	16.508846	8.912464
C	24.335684	16.556967	10.241534
C	24.053824	17.673375	11.033873
C	23.289950	18.730960	10.554573
C	22.806145	18.680480	9.254087
N	24.081893	15.339666	8.126991
C	25.162719	15.305030	7.343801
C	25.942132	16.582503	7.082837
C	25.210223	15.449262	10.812485
C	24.740304	14.978626	12.194670
C	22.523650	17.627264	6.996201
C	20.989557	17.581168	6.988714
C	25.681865	14.160883	6.708289
C	25.442211	12.774483	6.876048
C	26.541387	11.910449	6.276090
N	24.410080	12.234050	7.509006
C	24.373435	10.821674	7.732065
C	23.816798	9.944458	6.766387
C	23.745312	8.578975	7.057734
C	24.207880	8.065320	8.262836
C	24.760172	8.929019	9.200402
C	24.852840	10.303529	8.960938
C	23.328565	10.422391	5.406390
C	21.861279	10.046649	5.154314
C	25.520377	11.189290	10.002687
C	24.911585	11.017773	11.400506
C	24.206503	9.885295	4.263963
C	27.036184	10.938318	10.047520
C	26.684544	15.880308	10.869961
C	23.005571	18.858867	6.213160
Ca	22.499614	13.549945	8.133936
C	21.259358	13.598375	10.326814
C	20.246289	13.353828	11.458574
C	20.760265	13.564631	12.890265
C	19.707747	13.327854	13.974534
C	20.238724	13.525030	15.395710
C	19.178431	13.298864	16.471625
C	20.176702	13.798214	6.575785
C	21.252852	14.001879	5.504080
C	20.746485	14.496164	4.141948
C	21.852884	14.688329	3.103901
C	21.338105	15.169618	1.746002
C	22.448935	15.360180	0.715289
Ca	19.179123	12.929360	8.921306
N	17.113641	14.121117	9.181740
C	17.009362	15.437729	8.638692
C	17.391870	16.583338	9.379971
C	17.344144	17.837421	8.760317
C	16.924482	17.984099	7.445313
C	16.538803	16.858538	6.725760
C	16.573085	15.582801	7.294178
C	17.843057	16.513284	10.831738
C	19.315864	16.918136	10.984432
C	16.084164	14.385353	6.492298
C	16.442317	14.456938	5.003570
C	16.135186	13.630549	9.936099
C	16.025595	12.292014	10.378858
C	16.686690	11.101814	10.004906
C	16.024020	9.823698	10.486111
C	15.001020	14.527313	10.407159
N	17.791988	11.024207	9.265599
C	18.214545	9.740037	8.799864

C	17.806366	9.302690	7.514600
C	18.258869	8.067226	7.041888
C	19.104940	7.266656	7.799018
C	19.507752	7.703683	9.054722
C	19.073973	8.925280	9.577802
C	16.874840	10.128052	6.638752
C	17.499059	10.432241	5.269618
C	19.561448	9.345341	10.955835
C	21.049507	9.721138	10.914689
C	16.966140	17.377837	11.751677
C	14.567335	14.197263	6.659242
C	15.508981	9.447155	6.460429
C	19.318057	8.274709	12.029928
H	14.851255	15.383099	9.745645
H	14.063962	13.968971	10.482286
H	15.229851	14.918215	11.406543
H	15.172504	12.127749	11.028923
H	16.698709	9.252580	11.132243
H	15.106128	10.030374	11.040694
H	15.780590	9.171699	9.640844
H	17.741853	15.473511	11.162177
H	15.903098	17.139351	11.644531
H	17.247387	17.224418	12.800715
H	17.089056	18.445235	11.532706
H	19.471548	17.962328	10.689616
H	19.640934	16.815560	12.026813
H	19.975413	16.303321	10.364090
H	17.638647	18.718002	9.326738
H	16.891801	18.968353	6.984498
H	16.199895	16.976316	5.700154
H	16.566328	13.494498	6.912812
H	14.026295	15.076999	6.288717
H	14.221786	13.323533	6.092872
H	14.291077	14.048426	7.707257
H	17.509946	14.647639	4.852808
H	16.192208	13.509408	4.512750
H	15.881036	15.242075	4.482653
H	16.702502	11.081269	7.150063
H	15.017541	9.270254	7.422981
H	14.842728	10.072294	5.853132
H	15.610248	8.478461	5.955630
H	17.659323	9.517014	4.687048
H	16.840280	11.083176	4.682427
H	18.469517	10.929884	5.371187
H	17.939063	7.725090	6.059919
H	19.448352	6.309751	7.413498
H	20.175119	7.080751	9.645587
H	18.995061	10.236811	11.249308
H	19.921989	7.377442	11.850697
H	19.593469	8.661042	13.018794
H	18.268287	7.963292	12.068157
H	21.248066	10.499826	10.169743
H	21.387026	10.083892	11.893622
H	21.666425	8.856843	10.643311
H	21.646387	14.627254	10.462471
H	22.128354	12.945291	10.563777
H	19.365263	14.010460	11.336130
H	19.853422	12.320815	11.411372
H	21.151977	14.588526	12.972627
H	21.617381	12.895478	13.054693
H	19.304567	12.308290	13.877556
H	18.856202	14.005636	13.810490
H	21.083557	12.841845	15.563962
H	20.647397	14.541310	15.491145
H	18.774855	12.279885	16.421926
H	19.588521	13.445664	17.477513
H	18.337169	13.992646	16.350510
H	26.354040	10.843085	6.395685

H	27.502297	12.147740	6.746338
H	26.646718	12.131855	5.207777
H	26.549031	14.365388	6.087430
H	25.402973	17.214177	6.367262
H	26.929591	16.371171	6.666252
H	26.063953	17.172558	7.995302
H	23.405880	11.515533	5.393360
H	25.257650	10.162513	4.388485
H	23.863334	10.280987	3.299863
H	24.155344	8.791032	4.210384
H	21.735143	8.960836	5.069206
H	21.509325	10.491029	4.214619
H	21.215018	10.390902	5.966928
H	23.321124	7.902949	6.319193
H	24.145826	6.998955	8.465844
H	25.138837	8.527067	10.137330
H	25.369011	12.230095	9.696360
H	27.254429	9.910827	10.364264
H	27.520967	11.618909	10.758359
H	27.497477	11.087577	9.065863
H	23.835880	11.223272	11.399151
H	25.387038	11.704427	12.110459
H	25.054868	10.000943	11.784676
H	25.144723	14.596145	10.128300
H	27.066354	16.150621	9.880180
H	27.309145	15.067373	11.260563
H	26.813651	16.749070	11.527580
H	24.827193	15.769680	12.949098
H	25.355136	14.137975	12.537075
H	23.696679	14.649267	12.170832
H	24.445603	17.717406	12.047255
H	23.079530	19.589404	11.187824
H	22.214708	19.509935	8.872731
H	22.895040	16.736245	6.477065
H	22.597005	19.784312	6.636240
H	22.673793	18.801250	5.169215
H	24.096592	18.949655	6.219735
H	20.604159	16.720289	7.543598
H	20.602781	17.526894	5.964226
H	20.565660	18.477409	7.456076
H	19.337509	13.255141	6.105200
H	19.764167	14.775036	6.877139
H	21.795728	13.055269	5.333738
H	22.012299	14.730899	5.843499
H	20.007649	13.774943	3.763757
H	20.205382	15.441867	4.286695
H	22.592161	15.408480	3.486062
H	22.395942	13.740683	2.969541
H	20.795001	16.116215	1.880004
H	20.601446	14.449098	1.362641
H	23.183390	16.100280	1.056552
H	22.050250	15.705503	-0.245516
H	22.986624	14.421185	0.534513
H	20.984440	11.779701	7.800610
H	20.668576	12.567174	7.349127

186

[F2] scf done: -4306.954184 / Energies= -4305.203734 / Enthalpies= -4305.202790 / Free Energies= -4305.447600

C	0.889270	4.255377	-0.294140
C	1.928388	3.448980	0.233796
C	2.491137	3.754421	1.501426
C	1.996496	4.851897	2.211427
C	0.963953	5.635665	1.707989
C	0.420084	5.331988	0.465791
N	2.362229	2.278233	-0.452718
C	3.330188	2.355549	-1.360894
C	3.855681	3.712336	-1.792304

C	3.654215	2.954841	2.075510
C	3.421906	2.517610	3.527877
C	0.282869	3.999025	-1.667130
C	-1.168354	3.508705	-1.569240
C	3.931168	1.251784	-1.999662
C	3.795197	-0.136131	-1.790422
C	4.737435	-1.001972	-2.606506
N	2.918864	-0.704329	-0.967187
C	2.959865	-2.111282	-0.742810
C	2.239623	-2.999317	-1.580804
C	2.223252	-4.361353	-1.262913
C	2.913002	-4.859261	-0.164154
C	3.632024	-3.985195	0.643681
C	3.665477	-2.612364	0.382835
C	1.500328	-2.522519	-2.823704
C	-0.021811	-2.640754	-2.664453
C	4.498305	-1.702306	1.276416
C	4.203749	-1.898831	2.769574
C	1.957506	-3.270942	-4.085808
C	6.001081	-1.884651	1.008687
C	4.972576	3.738120	1.966239
C	0.361453	5.240332	-2.569719
Ca	1.068393	0.361244	0.069609
C	0.271997	-0.104870	2.436568
C	-0.535182	-0.461339	3.696499
C	0.235669	-0.455237	5.024802
C	-0.614810	-0.815898	6.243765
C	0.165808	-0.808044	7.559440
C	-0.691537	-1.167965	8.771162
C	1.926012	1.275696	-7.012878
C	3.415529	1.404902	-7.324739
C	3.737345	1.258271	-8.812672
C	5.227513	1.384577	-9.131588
C	5.550889	1.237740	-10.619249

C	7.041204	1.366774	-10.927222
Ca	-2.055427	-0.446782	1.304320
N	-4.046833	0.658192	1.999179
C	-4.202672	2.044055	1.685661
C	-3.713032	3.052939	2.553815
C	-3.834645	4.392932	2.172629
C	-4.429164	4.757026	0.970895
C	-4.906548	3.764611	0.123846
C	-4.803056	2.410020	0.453828
C	-3.048322	2.734742	3.885303
C	-1.554901	3.092258	3.854112
C	-5.353467	1.378045	-0.520025
C	-4.643041	1.452638	-1.879156
C	-4.990429	0.049007	2.712174
C	-5.078399	-1.341640	2.950951
C	-4.443143	-2.453733	2.354038
C	-5.080654	-3.795993	2.662414
C	-6.118348	0.871350	3.307387
N	-3.390159	-2.406441	1.541079
C	-3.005133	-3.593436	0.843775
C	-3.537493	-3.840214	-0.447489
C	-3.116924	-4.972559	-1.151076
C	-2.185948	-5.855196	-0.617191
C	-1.663507	-5.606161	0.645660
C	-2.057796	-4.492840	1.394640
C	-4.562843	-2.919163	-1.093298
C	-4.029900	-2.317698	-2.401440
C	-1.446949	-4.289727	2.772875
C	0.047695	-3.953129	2.669396
C	-3.725952	3.441146	5.069965
C	-6.873065	1.517209	-0.698973
C	-5.900017	-3.634800	-1.339201
C	-1.646390	-5.503360	3.693753
H	-6.684927	1.373273	2.515305

H	-6.808023	0.250710	3.883338
H	-5.731930	1.660986	3.958659
H	-5.892139	-1.614628	3.613120
H	-4.348483	-4.492690	3.082313
H	-5.909955	-3.695800	3.365934
H	-5.459518	-4.260952	1.745793
H	-3.137657	1.654945	4.051285
H	-4.799599	3.229795	5.116937
H	-3.275750	3.113964	6.014974
H	-3.607152	4.529490	5.009772
H	-1.411331	4.169990	3.716159
H	-1.065429	2.805724	4.792982
H	-1.032183	2.592007	3.032354
H	-3.457834	5.167848	2.836292
H	-4.521131	5.805504	0.697836
H	-5.375309	4.046038	-0.816590
H	-5.162210	0.386111	-0.096461
H	-7.137282	2.483946	-1.144867
H	-7.254855	0.730510	-1.361072
H	-7.401481	1.437611	0.257261
H	-3.566029	1.279321	-1.779041
H	-5.045537	0.698432	-2.565054
H	-4.780630	2.433215	-2.350651
H	-4.752215	-2.094250	-0.398387
H	-6.313768	-4.050423	-0.414054
H	-6.637404	-2.936538	-1.753634
H	-5.787105	-4.460301	-2.052587
H	-3.791038	-3.098726	-3.133297
H	-4.779719	-1.659197	-2.855304
H	-3.120401	-1.731238	-2.232203
H	-3.531009	-5.166642	-2.138131
H	-1.872300	-6.731517	-1.179363
H	-0.934116	-6.295288	1.065196
H	-1.957377	-3.438422	3.237973

H	-1.097982	-6.379785	3.328802
H	-1.274818	-5.280327	4.701282
H	-2.701233	-5.786297	3.777527
H	0.224715	-3.071997	2.043239
H	0.472439	-3.756000	3.661388
H	0.608645	-4.781282	2.221888
H	0.724426	0.890378	2.632061
H	1.137512	-0.800584	2.421845
H	-1.386591	0.233252	3.818967
H	-0.988298	-1.463936	3.592738
H	0.680664	0.540402	5.165918
H	1.079233	-1.155849	4.941684
H	-1.063136	-1.810269	6.095680
H	-1.459057	-0.113798	6.320864
H	1.008005	-1.510861	7.484132
H	0.612893	0.185373	7.708527
H	-1.122564	-2.171540	8.667498
H	-0.105746	-1.151552	9.697501
H	-1.523679	-0.463013	8.890912
H	5.074506	-1.876529	-2.044292
H	5.610676	-0.435250	-2.938910
H	4.222157	-1.376847	-3.499455
H	4.684692	1.518798	-2.733122
H	3.188251	4.149295	-2.545479
H	4.850847	3.631113	-2.236474
H	3.895610	4.416199	-0.956982
H	1.736608	-1.461818	-2.965901
H	3.045000	-3.232447	-4.212443
H	1.493547	-2.830552	-4.976564
H	1.666266	-4.327582	-4.053624
H	-0.324987	-3.676748	-2.474639
H	-0.528894	-2.301807	-3.576444
H	-0.393285	-2.027302	-1.835847
H	1.663186	-5.046190	-1.895213

H	2.895760	-5.923474	0.058276
H	4.182869	-4.377814	1.495278
H	4.244319	-0.667867	1.017769
H	6.317763	-2.910727	1.233975
H	6.589975	-1.205263	1.637328
H	6.254178	-1.679064	-0.036283
H	3.139451	-1.771131	2.993690
H	4.763856	-1.167621	3.364705
H	4.499688	-2.895896	3.117132
H	3.761860	2.049939	1.466492
H	5.203827	3.998949	0.928635
H	5.807394	3.143538	2.357566
H	4.924575	4.670492	2.542630
H	3.361172	3.375126	4.208543
H	4.252872	1.889250	3.870470
H	2.497639	1.940330	3.634121
H	2.432094	5.100617	3.176343
H	0.590566	6.482998	2.277992
H	-0.383918	5.948326	0.070627
H	0.867702	3.205499	-2.146359
H	-0.280488	6.048435	-2.199304
H	0.024171	4.993410	-3.583666
H	1.381911	5.633332	-2.634679
H	-1.240649	2.556904	-1.030890
H	-1.587385	3.350873	-2.570771
H	-1.802899	4.234599	-1.048109
H	1.538685	0.298222	-7.326433
H	1.343008	2.044195	-7.535643
H	3.975061	0.647278	-6.757465
H	3.780412	2.379556	-6.969735
H	3.370598	0.283281	-9.168060
H	3.177919	2.016750	-9.380931
H	5.594167	2.359549	-8.776015
H	5.786687	0.626075	-8.562976
H	4.992033	1.995275	-11.187296
H	5.186227	0.263187	-10.974533
H	7.427389	2.345098	-10.615094
H	7.240880	1.257365	-11.999424
H	7.622776	0.599853	-10.400691
H	-1.010239	0.056210	-0.552206
H	1.731123	1.382335	-5.939364

168

[F3] scf done: -4071.120272 / Energies= -4069.557019 / Enthalpies= -4069.556075 / Free Energies= -4069.774684

C	-3.768302	-0.717346	2.744794
C	-2.475630	-1.200899	3.072701
C	-2.191237	-2.584659	2.966008
C	-3.198320	-3.450393	2.528159
C	-4.467925	-2.983703	2.210062
C	-4.742431	-1.625436	2.321110
N	-1.456363	-0.273977	3.445932
C	-1.329377	0.045732	4.733342
C	-2.149792	-0.700738	5.768368
C	-0.825230	-3.156472	3.316882
C	-0.109196	-3.689546	2.068187
C	-4.124995	0.759862	2.841342
C	-4.425944	1.354614	1.457616
C	-0.510031	1.069593	5.242982
C	0.235977	2.081016	4.598861
C	0.659222	3.212609	5.521348
N	0.516618	2.125640	3.301508
C	1.208734	3.259025	2.782802
C	2.612822	3.192884	2.584590
C	3.261337	4.294734	2.017876
C	2.560477	5.434374	1.637555
C	1.185322	5.485335	1.825532
C	0.488797	4.416860	2.397957

C	3.419665	1.968991	3.002674
C	4.604295	1.679434	2.073126
C	-1.019364	4.526472	2.574106
C	-1.741609	4.508416	1.218602
C	3.924105	2.075589	4.451347
C	-1.423903	5.772512	3.375500
C	-0.905959	-4.257594	4.385136
C	-5.299229	1.012356	3.798788
Ca	-0.169463	0.539140	1.626557
Ca	0.174534	-0.523919	-1.680167
N	-0.544994	-2.287427	-3.165521
C	-1.225253	-3.362517	-2.518460
C	-2.616368	-3.267433	-2.264057
C	-3.247806	-4.302973	-1.568124
C	-2.546356	-5.424783	-1.145497
C	-1.185012	-5.520036	-1.413606
C	-0.499833	-4.504902	-2.088517
C	-3.451639	-2.091518	-2.752288
C	-3.988669	-1.249674	-1.587008
C	0.984109	-4.673687	-2.391130
C	1.764896	-5.317303	-1.237213
C	-0.507670	-2.258391	-4.492999
C	-1.184249	-3.345241	-5.314318
C	0.072602	-1.242676	-5.283070
C	0.548799	0.045145	-4.975216
C	0.887011	0.895687	-6.186719
N	0.661805	0.564398	-3.753404
C	1.035761	1.937046	-3.631105
C	2.396772	2.296830	-3.460105
C	2.723019	3.643060	-3.270883
C	1.745033	4.629784	-3.243350
C	0.413727	4.272713	-3.421116
C	0.033976	2.942514	-3.626101
C	3.517295	1.266494	-3.495929
C	4.315776	1.252683	-2.185553
C	-1.437821	2.627810	-3.862941
C	-2.293991	2.922617	-2.622808
C	-1.996216	3.384973	-5.078584
C	4.469048	1.485158	-4.682756
C	-4.607140	-2.551038	-3.655420
C	1.219294	-5.488078	-3.674388
C	1.725190	-0.679668	0.439123
C	2.531468	-1.643549	-0.445042
C	3.981041	-1.960662	-0.027903
C	4.112725	-2.696941	1.304438
C	5.560317	-3.034054	1.667347
C	5.691729	-3.764119	3.002484
H	2.607946	-1.260416	-1.485092
H	-1.147066	0.593413	-0.324906
H	1.519107	3.769116	5.144882
H	-0.169639	3.923558	5.628834
H	0.888604	2.832227	6.521194
H	-0.556501	1.179878	6.321283
H	-3.221018	-0.536664	5.607739
H	-1.988630	-1.780745	5.693542
H	-1.900748	-0.381007	6.782687
H	2.745364	1.103687	2.961986
H	3.102186	2.132132	5.169289
H	4.530885	1.198304	4.707949
H	4.549921	2.967649	4.579161
H	5.408274	2.415530	2.195168
H	5.030547	0.697475	2.307334
H	4.307711	1.678466	1.019449
H	4.336796	4.261369	1.867755
H	3.085631	6.276779	1.194197
H	0.638033	6.374899	1.521786
H	-1.354477	3.649836	3.138849
H	-2.504705	5.770946	3.561592

H	-0.911967	5.814274	4.342859	H	-4.582865	-0.408378	-1.965078
H	-1.185758	6.696347	2.834926	H	-3.179319	-0.837570	-0.974597
H	-1.521213	3.598908	0.648394	H	-4.313191	-4.230515	-1.362980
H	-2.828123	4.561497	1.357863	H	-3.057751	-6.225155	-0.616094
H	-1.440516	5.361614	0.598842	H	-0.642464	-6.405491	-1.092735
H	-3.252468	1.283923	3.246178	H	1.396452	-3.672562	-2.565285
H	-6.223132	0.546087	3.435743	H	2.294327	-5.612988	-3.854954
H	-5.099533	0.615730	4.800275	H	0.787922	-5.002920	-4.553438
H	-5.488689	2.088513	3.894544	H	0.773801	-6.486960	-3.586772
H	-4.637756	2.428096	1.537683	H	1.552467	-4.835824	-0.277190
H	-3.583751	1.222280	0.769087	H	2.842259	-5.244142	-1.426380
H	-5.301834	0.876322	1.002394	H	1.530456	-6.383586	-1.132529
H	-5.736390	-1.259540	2.072818	H	3.054836	0.280566	-3.621335
H	-5.239214	-3.674772	1.878869	H	4.975777	2.455341	-4.612316
H	-2.983008	-4.512313	2.437051	H	3.941370	1.454946	-5.641460
H	-0.220532	-2.340618	3.729724	H	5.241735	0.706740	-4.700631
H	-1.446249	-5.137365	4.015730	H	5.070067	0.456878	-2.201147
H	0.100981	-4.585158	4.670871	H	3.664923	1.087554	-1.320792
H	-1.417612	-3.912054	5.289972	H	4.839431	2.202970	-2.026656
H	0.006902	-2.909737	1.306951	H	3.766243	3.923774	-3.143832
H	0.890210	-4.062231	2.324752	H	2.018758	5.670487	-3.088646
H	-0.670657	-4.510325	1.607796	H	-0.351654	5.045740	-3.409721
H	-1.366006	-4.259197	-4.747222	H	-1.519403	1.556141	-4.075100
H	-0.588928	-3.586538	-6.200423	H	-2.015902	4.467262	-4.902396
H	-2.154640	-2.977518	-5.670793	H	-3.025355	3.066378	-5.284357
H	0.042969	-1.454856	-6.347087	H	-1.400304	3.209589	-5.980716
H	1.031334	0.277115	-7.075851	H	-1.964418	2.336715	-1.757818
H	1.782789	1.499922	-6.026232	H	-3.345496	2.676030	-2.816728
H	0.070239	1.596891	-6.394909	H	-2.246394	3.984968	-2.353182
H	-2.802086	-1.447852	-3.355647	H	1.687158	-1.156362	1.441793
H	-4.251019	-3.170013	-4.485838	H	2.354598	0.221586	0.594119
H	-5.129387	-1.682753	-4.075462	H	1.992747	-2.600375	-0.527600
H	-5.344971	-3.139157	-3.096458	H	4.454940	-2.566280	-0.816211
H	-4.629057	-1.845936	-0.927116	H	4.547343	-1.020004	0.021054

H	3.674320	-2.088340	2.107295
H	3.524410	-3.626468	1.268034
H	6.150941	-2.106918	1.697138
H	6.001825	-3.647759	0.868886
H	5.290153	-3.160029	3.825742
H	6.738537	-3.990476	3.235923
H	5.140860	-4.712945	2.991085
H	1.452419	2.911067	-0.180250
H	0.912567	2.785262	-0.676774

168

[TS-F3F4] scf done: -4071.100274 / Energies= -4069.537193 / Enthalpies= -4069.536249 / Free Energies= -4069.749941

C	-3.966611	-0.653117	2.988912
C	-2.654769	-1.093905	3.294910
C	-2.368837	-2.481918	3.346138
C	-3.415329	-3.391670	3.163314
C	-4.711905	-2.965272	2.900427
C	-4.972710	-1.604359	2.796845
N	-1.619802	-0.134289	3.514480
C	-1.439243	0.321664	4.752858
C	-2.276919	-0.221613	5.898285
C	-0.964707	-3.013656	3.602831
C	-0.564258	-4.088148	2.581352
C	-4.308529	0.822721	2.842872
C	-4.672443	1.158816	1.387863
C	-0.559553	1.357569	5.127345
C	0.105041	2.350204	4.375744
C	0.659831	3.481814	5.226461
N	0.213731	2.377799	3.051702
C	0.737004	3.544773	2.418123
C	2.091743	3.583206	2.000329
C	2.557091	4.718543	1.330558
C	1.721422	5.795839	1.058985
C	0.394585	5.749279	1.468193
C	-0.119106	4.642794	2.151669
C	3.058760	2.447318	2.306129
C	3.959420	2.089847	1.117201
C	-1.575715	4.665252	2.595690
C	-2.534974	4.667099	1.396807
C	3.924044	2.758777	3.538686
C	-1.876436	5.859887	3.514270
C	-0.797594	-3.567027	5.026979
C	-5.432214	1.263170	3.792122
Ca	-0.412299	0.590341	1.600256
Ca	0.271301	-0.459609	-1.755128
N	-0.351221	-2.150365	-3.317004
C	-0.505745	-3.443053	-2.738400
C	-1.714378	-3.823326	-2.104427
C	-1.766933	-5.054526	-1.440075
C	-0.673274	-5.909618	-1.407751
C	0.500602	-5.544801	-2.060142
C	0.611344	-4.323535	-2.730009
C	-2.969002	-2.962633	-2.157671
C	-3.404456	-2.480745	-0.768491
C	1.887521	-3.977695	-3.487429
C	3.163993	-4.526229	-2.839181
C	-0.717685	-1.924423	-4.575525
C	-1.498135	-2.970832	-5.353447
C	-0.456938	-0.742948	-5.296737
C	0.293881	0.406507	-4.974202
C	0.477229	1.383737	-6.122692
N	0.806015	0.675985	-3.777201
C	1.620546	1.833747	-3.603452
C	3.031176	1.683559	-3.584840
C	3.826186	2.808323	-3.348104
C	3.261198	4.057607	-3.116461

C	1.878878	4.194417	-3.127751	H	-0.258360	6.594520	1.261254
C	1.038762	3.104498	-3.375411	H	-1.766301	3.752291	3.169370
C	3.682916	0.336035	-3.862269	H	-2.902280	5.799067	3.897910
C	4.924993	0.070632	-3.002472	H	-1.194285	5.893759	4.370149
C	-0.468326	3.318005	-3.377087	H	-1.781566	6.812427	2.979016
C	-0.998463	3.569894	-1.959013	H	-2.379036	3.795183	0.752597
C	-0.898204	4.458553	-4.312069	H	-3.577676	4.656539	1.737489
C	4.032756	0.180404	-5.351208	H	-2.396116	5.561745	0.777964
C	-4.128723	-3.704612	-2.842709	H	-3.413464	1.397506	3.106067
C	1.814548	-4.457271	-4.947095	H	-6.379887	0.765238	3.554264
C	1.440668	-1.145992	0.819746	H	-5.191926	1.035485	4.836264
C	2.471268	-1.680252	-0.176536	H	-5.599385	2.344059	3.710648
C	3.756293	-2.273448	0.422834	H	-4.873897	2.231125	1.276741
C	3.534015	-3.507668	1.295241	H	-3.866289	0.888589	0.695552
C	4.830875	-4.080887	1.869266	H	-5.571716	0.616006	1.071979
C	4.609196	-5.314165	2.742427	H	-5.982687	-1.271428	2.567882
H	2.802332	-0.861617	-0.853683	H	-5.511264	-3.689805	2.765003
H	-1.264611	0.225347	-0.368574	H	-3.208545	-4.457267	3.227117
H	1.367786	4.109970	4.683990	H	-0.270847	-2.170813	3.497287
H	-0.157848	4.123934	5.575362	H	-1.500756	-4.388902	5.210805
H	1.150612	3.076953	6.117531	H	0.217979	-3.956831	5.169062
H	-0.512981	1.525617	6.199185	H	-0.970302	-2.800566	5.788114
H	-3.212831	0.346672	5.969511	H	-0.736654	-3.760453	1.550923
H	-2.551972	-1.268864	5.762767	H	0.497317	-4.338691	2.688441
H	-1.753124	-0.109033	6.851465	H	-1.131573	-5.015142	2.727885
H	2.456364	1.563617	2.551757	H	-1.372532	-3.974847	-4.942895
H	3.318576	2.922437	4.434917	H	-1.204667	-2.978037	-6.407236
H	4.611739	1.928774	3.744075	H	-2.567707	-2.728974	-5.316302
H	4.526060	3.660389	3.370276	H	-0.839203	-0.753046	-6.312864
H	4.692043	2.879393	0.911133	H	0.410102	0.873386	-7.087007
H	4.527335	1.178044	1.339123	H	1.433986	1.908116	-6.063692
H	3.376418	1.923455	0.206477	H	-0.309616	2.147455	-6.096328
H	3.595407	4.759301	1.012726	H	-2.740416	-2.075407	-2.759251
H	2.104352	6.667541	0.533978	H	-3.844379	-4.089889	-3.827407

H	-4.985702	-3.032435	-2.972241
H	-4.467284	-4.555818	-2.239732
H	-3.629032	-3.320676	-0.101881
H	-4.309668	-1.866601	-0.844706
H	-2.632739	-1.867259	-0.292784
H	-2.689421	-5.349749	-0.945385
H	-0.736163	-6.861726	-0.886446
H	1.346671	-6.226616	-2.047866
H	1.962221	-2.883029	-3.513978
H	2.739669	-4.204948	-5.480255
H	0.982784	-3.997212	-5.486335
H	1.685357	-5.546053	-4.989929
H	3.228083	-4.276816	-1.775258
H	4.044907	-4.107841	-3.340013
H	3.232940	-5.616924	-2.932085
H	2.939026	-0.436521	-3.628281
H	4.741555	0.956991	-5.664806
H	3.144680	0.258344	-5.985541
H	4.495749	-0.796305	-5.540090
H	5.254701	-0.967532	-3.128561
H	4.728500	0.240072	-1.938196
H	5.767277	0.710539	-3.291649
H	4.908260	2.706117	-3.343017
H	3.896292	4.919624	-2.927416
H	1.439260	5.171018	-2.939850
H	-0.937574	2.397457	-3.741268
H	-0.550827	5.432214	-3.946794
H	-1.992198	4.505629	-4.374541
H	-0.503315	4.326750	-5.325392
H	-0.771332	2.729082	-1.294175
H	-2.087391	3.703257	-1.974570
H	-0.550430	4.468644	-1.519796
H	0.767813	-1.954253	1.142546
H	1.975792	-0.804732	1.725594
H	2.009525	-2.452691	-0.815607
H	4.452859	-2.523018	-0.390960
H	4.249392	-1.491338	1.018588
H	2.853742	-3.252593	2.119716
H	3.020529	-4.283937	0.707757
H	5.340764	-3.303226	2.456150
H	5.513133	-4.334280	1.044942
H	3.960285	-5.084003	3.596687
H	5.555125	-5.700961	3.138651
H	4.131594	-6.121606	2.173575
H	1.238432	0.229691	0.178342
H	1.210535	1.073019	-0.261147

168

[F4] scf done: -4071.174449 / Energies= -4069.606568 / Enthalpies= -4069.605623 / Free Energies= -4069.831178

C	-4.009204	-0.985792	2.857722
C	-2.643784	-1.335261	3.015153
C	-2.224701	-2.674548	2.813346
C	-3.175233	-3.626247	2.432569
C	-4.512825	-3.288025	2.261382
C	-4.919474	-1.976998	2.479602
N	-1.686267	-0.313850	3.280482
C	-1.352543	-0.030891	4.535587
C	-1.938125	-0.855784	5.664247
C	-0.775741	-3.101585	3.007066
C	-0.106025	-3.452262	1.670889
C	-4.510705	0.430451	3.107672
C	-5.076476	1.071008	1.831898
C	-0.493875	1.016369	4.924398
C	0.091276	2.050773	4.167527
C	0.834350	3.106992	4.961173
N	0.017097	2.161493	2.844493
C	0.553752	3.317007	2.205394

C	1.901471	3.332269	1.766128
C	2.362850	4.439896	1.049133
C	1.534839	5.522218	0.773764
C	0.218222	5.505977	1.219044
C	-0.295795	4.419392	1.933021
C	2.853383	2.179523	2.055414
C	3.164518	1.376327	0.784451
C	-1.740903	4.461018	2.411615
C	-2.729777	4.513072	1.237539
C	4.157164	2.649563	2.718106
C	-1.991402	5.634490	3.371261
C	-0.641506	-4.274123	3.990615
C	-5.555431	0.476736	4.233245
Ca	-0.876447	0.638290	1.273658
Ca	-0.917051	0.115721	-2.221643
N	-1.910237	-1.273538	-3.854581
C	-2.606945	-2.348185	-3.230989
C	-3.958619	-2.189736	-2.834327
C	-4.569304	-3.213908	-2.104456
C	-3.885280	-4.379099	-1.777208
C	-2.567235	-4.537174	-2.189939
C	-1.908146	-3.542126	-2.917457
C	-4.761059	-0.945996	-3.191967
C	-5.082584	-0.100483	-1.951553
C	-0.472436	-3.773069	-3.370420
C	0.495120	-3.874671	-2.182429
C	-1.908232	-1.160378	-5.178663
C	-2.718713	-2.134123	-6.010441
C	-1.189769	-0.186969	-5.900537
C	-0.280576	0.794762	-5.459326
C	0.404985	1.594832	-6.548835
N	0.013879	1.042201	-4.186716
C	1.013453	2.006470	-3.867577
C	2.355471	1.588512	-3.679128

C	3.299721	2.527644	-3.253673
C	2.948663	3.852016	-3.017483
C	1.633613	4.256629	-3.216265
C	0.651532	3.359213	-3.645339
C	2.795632	0.154396	-3.942786
C	3.252903	-0.549166	-2.656437
C	-0.769672	3.857664	-3.870651
C	-1.464897	4.203573	-2.545997
C	-0.819018	5.059893	-4.825705
C	3.897886	0.078688	-5.010420
C	-6.054135	-1.291612	-3.946648
C	-0.348179	-5.015622	-4.265225
C	5.118888	-3.695145	2.592890
C	6.389444	-2.958984	2.168576
C	7.409717	-2.751257	3.293542
C	8.010124	-4.039983	3.859542
C	9.084924	-3.790372	4.919256
C	9.680843	-5.076983	5.486934
H	6.112317	-1.977245	1.760597
H	-1.972958	1.164436	-0.572997
H	1.883953	3.160284	4.652300
H	0.408003	4.099872	4.781992
H	0.798813	2.902407	6.033554
H	-0.314121	1.080364	5.991661
H	-3.031557	-0.787028	5.664500
H	-1.694442	-1.916536	5.544112
H	-1.570798	-0.522742	6.637551
H	2.353218	1.504735	2.759442
H	3.966422	3.244978	3.618124
H	4.767960	1.785848	3.006968
H	4.759458	3.263209	2.037736
H	3.634374	2.009101	0.022319
H	3.848684	0.548189	1.008414
H	2.260190	0.946307	0.337701

H	3.392958	4.455719	0.701652
H	1.915684	6.375993	0.218689
H	-0.426763	6.357175	1.011458
H	-1.932007	3.536531	2.967600
H	-3.018850	5.602450	3.753970
H	-1.311419	5.609163	4.229824
H	-1.855384	6.599824	2.868784
H	-2.607422	3.657524	0.563483
H	-3.763076	4.509246	1.605809
H	-2.593220	5.424384	0.642848
H	-3.653692	1.032183	3.429802
H	-6.465243	-0.070888	3.959148
H	-5.172356	0.038544	5.161296
H	-5.844295	1.513751	4.443684
H	-5.402148	2.099470	2.030312
H	-4.334237	1.101435	1.026114
H	-5.944401	0.513942	1.459114
H	-5.968551	-1.716160	2.357393
H	-5.235248	-4.044054	1.964178
H	-2.860954	-4.654342	2.268355
H	-0.233673	-2.250475	3.434724
H	-1.096392	-5.189423	3.593645
H	0.416812	-4.489700	4.180480
H	-1.120771	-4.057820	4.951955
H	-0.107260	-2.604126	0.976243
H	0.937515	-3.751664	1.829412
H	-0.623886	-4.282954	1.176647
H	-2.461378	-3.169621	-5.764765
H	-2.558400	-1.978866	-7.079757
H	-3.788803	-2.022100	-5.802713
H	-1.317200	-0.235180	-6.976149
H	0.069329	1.290069	-7.542634
H	1.491965	1.469475	-6.495853
H	0.210356	2.666057	-6.429047
H	-4.143113	-0.334223	-3.859103
H	-5.859446	-1.913652	-4.827369
H	-6.554668	-0.375395	-4.282295
H	-6.760129	-1.836357	-3.308387
H	-5.684014	-0.669721	-1.232732
H	-5.651518	0.794978	-2.231886
H	-4.174300	0.229060	-1.433544
H	-5.603137	-3.096346	-1.788004
H	-4.379046	-5.162170	-1.207378
H	-2.037494	-5.455146	-1.944583
H	-0.172477	-2.906351	-3.969539
H	0.679200	-5.117933	-4.635365
H	-1.015108	-4.958425	-5.132422
H	-0.595116	-5.932828	-3.717104
H	0.464834	-2.979081	-1.551003
H	1.525659	-4.003322	-2.535867
H	0.252525	-4.733720	-1.545409
H	1.927801	-0.391496	-4.328945
H	4.820749	0.564978	-4.672006
H	3.592423	0.562399	-5.944739
H	4.139371	-0.967489	-5.234706
H	3.531769	-1.589223	-2.866371
H	2.467022	-0.557264	-1.892198
H	4.128209	-0.051415	-2.221508
H	4.331435	2.214590	-3.108296
H	3.697401	4.566924	-2.685410
H	1.362653	5.294130	-3.035964
H	-1.335572	3.044240	-4.338792
H	-0.336603	5.942992	-4.390141
H	-1.859951	5.328417	-5.042591
H	-0.319028	4.845570	-5.776874
H	-1.528510	3.337357	-1.876722
H	-2.487239	4.557926	-2.728569
H	-0.923990	4.991579	-2.008865

H	5.330726	-4.718570	2.922350
H	4.620298	-3.176114	3.421632
H	6.869155	-3.504902	1.343182
H	8.225969	-2.117825	2.916701
H	6.937989	-2.182900	4.109724
H	7.217533	-4.663206	4.296951
H	8.442466	-4.630445	3.037143
H	8.655032	-3.193546	5.736513
H	9.885892	-3.175238	4.484488
H	8.908882	-5.695943	5.960843
H	10.447583	-4.866388	6.241414
H	10.147302	-5.679613	4.697684
H	4.405226	-3.758365	1.763305
H	0.169145	-0.427881	-0.371359

18

[Hexene]

C	21.277124	13.336836	5.541683
C	20.985690	13.275225	4.071232
C	22.047551	13.986662	3.220210
C	21.775655	13.896538	1.718092
C	22.833305	14.604485	0.873881
C	20.492300	13.900249	6.460605
H	20.763682	13.918784	7.513228
H	19.543202	14.362729	6.194081
H	22.220988	12.887179	5.857885
H	20.931358	12.221713	3.754490
H	19.997568	13.711596	3.871689
H	22.101549	15.041617	3.523370
H	23.036469	13.556352	3.437517
H	20.786686	14.325938	1.503089
H	21.717576	12.838933	1.423038
H	22.892368	15.670786	1.124612
H	22.610019	14.526365	-0.196352
H	23.827670	14.171388	1.038678

12

[Benzene]

C	21.367687	13.684368	5.588222
C	20.961742	13.643853	4.254353
C	21.863282	13.968892	3.240842
C	23.170664	14.334387	3.561267
C	23.576609	14.374903	4.895135
C	22.675069	14.049864	5.908647
H	19.942512	13.359038	4.004737
H	21.546922	13.937397	2.201042
H	23.873466	14.587617	2.771201
H	24.595839	14.659717	5.144752
H	22.991429	14.081358	6.948447
H	20.664885	13.431139	6.378288

30

[hexylbenzene]

C	1.610761	-0.359575	-2.186381
C	1.554189	-1.568136	-1.482440
C	1.964442	-1.645144	-0.152543
C	2.440765	-0.508548	0.499617
C	2.502309	0.702599	-0.188615
C	2.090564	0.773271	-1.518292
C	1.202260	-0.288161	-3.637952
C	2.370358	-0.541085	-4.604173
C	1.956230	-0.477776	-6.074526
C	3.113584	-0.720151	-7.044396
C	2.698718	-0.663809	-8.515510
C	3.860176	-0.901764	-9.478267
H	0.409855	-1.023186	-3.834601
H	0.769106	0.698672	-3.851411
H	2.810640	-1.524097	-4.384920
H	3.162812	0.195507	-4.409916
H	1.164091	-1.218553	-6.261183
H	1.506381	0.504527	-6.283971

H 3.903721 0.023604 -6.861757
H 3.566999 -1.700261 -6.833025
H 2.242921 0.314426 -8.725752
H 1.911875 -1.409708 -8.698133
H 4.647072 -0.149194 -9.344022
H 3.531577 -0.856163 -10.522946
H 4.315334 -1.886645 -9.315575
H 2.757595 -0.565134 1.537998
H 2.867071 1.596243 0.312320
H 2.136310 1.723858 -2.047042
H 1.178324 -2.458860 -1.983015
H 1.907381 -2.593517 0.376519

20

[hexane] scf done: -236.997222 / Energies= -236.798164 / Enthalpies= -236.797219 / Free Energies= -236.838809

C 4.839661 -3.514738 2.443914
C 6.083296 -2.727327 2.032233
C 7.112334 -2.534420 3.151929
C 7.756976 -3.826377 3.658916
C 8.839421 -3.586721 4.713141
C 9.481855 -4.876147 5.220441
H 5.774080 -1.738717 1.666165
H 5.084471 -4.543049 2.732562
H 4.337104 -3.041493 3.297087
H 6.566820 -3.227445 1.180529
H 7.905003 -1.863189 2.790582
H 6.635637 -2.012320 3.995545
H 6.988814 -4.489018 4.081804
H 8.194294 -4.371651 2.808419
H 8.404350 -3.036009 5.559489
H 9.615193 -2.930834 4.292494
H 8.736075 -5.536542 5.680081
H 10.253527 -4.672427 5.971787
H 9.953971 -5.432690 4.401232
H 4.116958 -3.568666 1.621549

74

[A1-mono] scf done: -1917.054066 / Energies= -1916.373767 / Enthalpies= -1916.372822 / Free Energies= -1916.484445

Ca 22.391870 19.436559 8.205572
N 22.862160 21.220293 9.627007
N 21.221697 18.739006 10.094659
C 22.951256 22.687734 11.591755
H 22.612397 23.581871 11.056538
H 22.578467 22.734567 12.617357
H 24.045597 22.737378 11.615650
C 22.488558 21.431025 10.884521
C 21.674298 20.556459 11.633579
H 21.464222 20.886128 12.644928
C 21.088185 19.323505 11.280505
C 20.254541 18.646289 12.348021
H 20.652577 17.651878 12.578703
H 20.225439 19.231582 13.269798
H 19.227071 18.495370 11.998134
C 23.681028 22.155770 8.932921
C 25.088155 21.984921 8.915601
C 25.770289 20.881903 9.712066
H 25.003994 20.394898 10.326134
C 26.841133 21.428179 10.667590
H 26.430566 22.185443 11.344687
H 27.256400 20.617343 11.278008
H 27.673334 21.887546 10.121095
C 26.375691 19.817710 8.781948
H 27.177056 20.243601 8.166323
H 26.803840 18.992716 9.364352
H 25.633865 19.397552 8.090514

C 25.860989 22.841234 8.125950
H 26.941001 22.714775 8.106064
C 25.278595 23.845279 7.361418
H 25.897552 24.498511 6.751664
C 23.897301 24.001327 7.375528
H 23.443025 24.781791 6.769242
C 23.079340 23.171990 8.147859
C 21.569135 23.358126 8.104273
H 21.131655 22.695892 8.859860
C 21.140919 24.791348 8.449749
H 20.047318 24.865370 8.484051
H 21.531977 25.103244 9.424325
H 21.495773 25.512116 7.703704
C 21.000863 22.948405 6.735819
H 21.275623 21.920492 6.463290
H 19.906364 23.018761 6.728805
H 21.384975 23.595581 5.938512
C 20.604914 17.487055 9.813572
C 19.329529 17.454682 9.194864
C 18.545182 18.729249 8.916292
H 19.080594 19.558795 9.392260
C 17.132545 18.693149 9.515769
H 16.515213 17.914783 9.052045
H 17.159748 18.496601 10.593105
H 16.625319 19.652788 9.359134
C 18.477956 19.017698 7.407730
H 17.972327 19.971100 7.212195
H 19.474953 19.062984 6.948757
H 17.928467 18.230053 6.878937
C 18.796998 16.221249 8.809181
H 17.820052 16.188810 8.331953
C 19.492920 15.036079 9.017115
H 19.063542 14.086849 8.706997
C 20.746660 15.075755 9.616112
H 21.293639 14.148072 9.768046
C 21.324276 16.282866 10.019969
C 22.714122 16.275836 10.641099
H 22.935157 17.297509 10.970662
C 22.797082 15.368816 11.877129
H 22.633104 14.316262 11.617741
H 23.788913 15.443236 12.338971
H 22.051462 15.644456 12.631155
C 23.781335 15.874739 9.609606
H 23.760489 16.515258 8.718611
H 24.784973 15.934129 10.048173
H 23.627957 14.845640 9.263178
H 23.127241 18.631736 6.465093

92

[TS-A1A2-mono] scf done: -2152.813363 / Energies= -2151.956189 / Enthalpies= -2151.955245 / Free Energies= -2152.085202

C 20.606444 16.623162 10.843599
C 20.114012 17.730380 10.104227
C 19.153250 17.527854 9.079299
C 18.733601 16.223384 8.798831
C 19.229361 15.132842 9.503355
C 20.153476 15.340801 10.521450
N 20.684238 19.019905 10.295509
C 20.211549 19.858879 11.209094
C 19.019632 19.449129 12.049898
C 18.545390 18.686089 8.298606
C 18.857221 18.604065 6.796839
C 21.612719 16.795458 11.973808
C 22.979886 16.194475 11.614293
C 20.754918 21.130613 11.484877
C 21.885814 21.781836 10.950719
C 22.204907 23.143707 11.532831

N	22.665677	21.284790	9.997356
C	23.810433	21.993983	9.535981
C	25.077288	21.743502	10.125509
C	26.214153	22.328161	9.560032
C	26.126651	23.148290	8.440857
C	24.881388	23.404082	7.878952
C	23.711773	22.847359	8.406020
C	25.230060	20.864518	11.360367
C	26.048059	19.599000	11.064185
C	22.373930	23.192201	7.764619
C	22.270442	22.667479	6.324161
C	25.854587	21.631353	12.536274
C	22.104032	24.704836	7.797711
C	17.027587	18.778008	8.524022
C	21.104853	16.198375	13.294694
Ca	22.428060	19.305868	8.745771
C	24.142736	17.536301	7.846861
C	23.676405	17.925800	6.608162
C	24.502645	18.762368	5.660915
C	25.516050	17.902590	4.888029
C	26.327801	18.712578	3.875895
C	27.339559	17.866682	3.105472
H	22.271512	19.224508	6.690129
H	23.215278	23.159318	11.955430
H	22.183140	23.912419	10.752699
H	21.496398	23.424476	12.315374
H	20.232446	21.688700	12.253930
H	18.625548	20.290445	12.624726
H	18.218281	19.040958	11.426246
H	19.301631	18.657929	12.754782
H	24.227048	20.547858	11.667245
H	25.287484	22.537023	12.776976
H	25.881192	21.001068	13.433503
H	26.884634	21.936007	12.315324

H	27.069718	19.847581	10.752784
H	26.115954	18.963739	11.955695
H	25.598757	19.003305	10.261306
H	27.187619	22.138426	10.007294
H	27.023019	23.590612	8.013187
H	24.812850	24.054586	7.009805
H	21.588346	22.704453	8.353172
H	21.100039	24.920071	7.412605
H	22.172030	25.110435	8.813204
H	22.820898	25.253228	7.174846
H	22.371460	21.575857	6.277667
H	21.296698	22.931616	5.893083
H	23.046585	23.106758	5.685263
H	18.989429	19.612544	8.680816
H	16.509824	17.897861	8.123871
H	16.777580	18.853348	9.588046
H	16.619949	19.661066	8.017288
H	18.402275	19.450934	6.268219
H	19.936704	18.632718	6.602728
H	18.456471	17.683492	6.354915
H	18.000156	16.060777	8.012225
H	18.892367	14.126914	9.265642
H	20.531844	14.487377	11.080052
H	21.756487	17.870079	12.130111
H	21.005947	15.108032	13.233856
H	21.805644	16.420331	14.108555
H	20.125053	16.603014	13.570631
H	23.396927	16.645606	10.706440
H	23.699269	16.351417	12.427317
H	22.905386	15.114674	11.436839
H	23.758162	16.637107	8.327286
H	25.084998	17.933616	8.226883
H	22.918148	17.307901	6.131720
H	23.843057	19.281543	4.957483

H 25.042137 19.539271 6.220288
H 26.194090 17.410160 5.598125
H 24.983558 17.095049 4.363524
H 26.852029 19.524950 4.399707
H 25.641959 19.200298 3.168573
H 28.059995 17.394192 3.784659
H 27.905918 18.472667 2.388776
H 26.841672 17.066292 2.544157
92
[A2-mono] scf done: -2152.847538 / Energies= -2151.985083 / Enthalpies= -2151.984139 / Free Energies= -2152.119329
Ca 23.682212 13.987019 7.510310
N 25.585550 12.656462 7.827030
N 25.196763 15.646236 8.171139
C 27.878316 11.943678 8.331843
H 27.586523 11.194614 9.076608
H 28.840392 12.368797 8.626354
H 28.008745 11.405818 7.386258
C 26.810463 13.010179 8.200561
C 27.210418 14.330607 8.495115
H 28.251209 14.433107 8.781346
C 26.483301 15.539434 8.485422
C 27.255937 16.786638 8.863051
H 27.244995 17.511703 8.041178
H 28.295079 16.558848 9.110849
H 26.793526 17.285227 9.722210
C 25.262113 11.298626 7.549492
C 25.348056 10.811123 6.220519
C 25.892884 11.671315 5.088386
H 26.286783 12.592459 5.532672
C 27.049486 10.993044 4.340454
H 27.857939 10.709652 5.023107
H 27.465543 11.670427 3.585010
H 26.719191 10.085106 3.822345
C 24.780483 12.063830 4.103084
H 24.357382 11.179886 3.611572
H 25.166733 12.730263 3.322231
H 23.945709 12.574677 4.600929
C 24.898604 9.516369 5.945627
H 24.959762 9.136488 4.928161
C 24.371756 8.705921 6.944778
H 24.025164 7.702564 6.710739
C 24.286610 9.191420 8.244684
H 23.867887 8.557870 9.023452
C 24.721081 10.478878 8.572161
C 24.581687 10.974874 10.004396
H 25.053421 11.962655 10.058712
C 25.303764 10.065631 11.009153
H 24.855528 9.065548 11.043853
H 25.243277 10.488859 12.019009
H 26.362800 9.945074 10.755605
C 23.104359 11.139075 10.395930
H 22.566012 11.801030 9.706096
H 23.014771 11.555384 11.406759
H 22.582863 10.174510 10.384162
C 24.534785 16.905989 8.189938
C 23.860180 17.333025 9.361684
C 23.914947 16.528177 10.652325
H 24.642491 15.721447 10.508530
C 24.390780 17.366946 11.847143
H 25.361337 17.835799 11.650339
H 24.493849 16.736363 12.738391
H 23.679770 18.165317 12.090198
C 22.554736 15.881641 10.960379
H 21.787864 16.643943 11.143349
H 22.618754 15.249450 11.854494

H	22.194424	15.261160	10.130132
C	23.106169	18.508913	9.314271
H	22.582544	18.841350	10.207703
C	23.004679	19.258605	8.147787
H	22.409947	20.168372	8.131176
C	23.664638	18.832009	7.001138
H	23.578371	19.417836	6.088486
C	24.432067	17.663589	6.995852
C	25.115179	17.220231	5.709891
H	25.738768	16.351514	5.950670
C	26.040430	18.301199	5.133339
H	25.479195	19.189788	4.821119
H	26.571072	17.920281	4.252504
H	26.788115	18.621977	5.866813
C	24.086062	16.783603	4.654820
H	23.415871	15.997052	5.026999
H	24.584918	16.404538	3.754600
H	23.445892	17.622104	4.356333
C	21.256038	13.726925	7.304368
H	20.905846	12.864426	7.905570
H	20.746025	14.595062	7.767732
C	20.683435	13.558634	5.885298
H	21.139472	12.682836	5.390287
H	20.961010	14.423465	5.256285
C	19.158206	13.395621	5.800673
H	18.863824	12.523850	6.405113
H	18.683970	14.267429	6.277069
C	18.617811	13.235232	4.378193
H	18.912696	14.109163	3.776257
H	19.098047	12.365951	3.902064
C	17.098905	13.068278	4.307057
H	16.617727	13.935405	4.782171
H	16.803534	12.193981	4.905058
C	16.571243	12.911234	2.882036
H	16.820420	13.786466	2.268777
H	15.481202	12.793046	2.864945
H	17.008713	12.031872	2.392572

References

1. A. S. S. Wilson, M. S. Hill, M. F. Mahon, C. Dinoi, L. Maron, *Science*, **2017**, *358*, 1168.
2. O. V. Dolomanov, L. J. Bourhis, R. J. Gildea, J. A. K. Howard and H. Puschmann, *J. Appl. Cryst.*, **2009**, *42*, 339-341.
3. L. J. Bourhis, O. V. Dolomanov, R. J. Gildea, J. A. K. Howard, H. Puschmann, *Acta Cryst. a-Foundation and Advances*, **2015**, *71*, 59-75.
4. G. M. Sheldrick, *Acta Cryst. C-Structural Chemistry*, **2015**, *71*, 3-8.
5. Gaussian09, revision D.01. Frisch, M. J.; Trucks, G. W.; Schlegel, H. B.; Scuseria, G. E.; Robb, M. A.; Cheeseman, J. R.; Scalmani, G.; Barone, V.; Mennucci, B.; Petersson, G. A.; Nakatsuji, H.; Caricato, M.; Li, X.; Hratchian, H. P.; Izmaylov, A. F.; Bloino, J.; Zheng, G.; Sonnenberg, J. L.; Hada, M.; Ehara, M.; Toyota, K.; Fukuda, R.; Hasegawa, J.; Ishida, M.; Nakajima, T.; Honda, Y.; Kitao, O.; Nakai, H.; Vreven, T.; Montgomery, Jr., J. A.; Peralta, J. E.; Ogliaro, F.; Bearpark, M.; Heyd, J. J.; Brothers, E.; Kudin, K. N.; Staroverov, V. N.; Keith, T.; Kobayashi, R.; Normand, J.; Raghavachari, K.; Rendell, A.; Burant, J. C.; Iyengar, S. S.; Tomasi, J.; Cossi, M.; Rega, N.; Millam, J. M.; Klene, M.; Knox, J. E.; Cross, J. B.; Bakken, V.; Adamo, C.; Jaramillo, J.; Gomperts, R.; Stratmann, R. E.; Yazyev, O.; Austin, A. J.; Cammi, R.; Pomelli, C.; Ochterski, J. W.; Martin, R. L.; Morokuma, K.; Zakrzewski, V. G.; Voth, G. A.; Salvador, P.; Dannenberg, J. J.; Dapprich, S.; Daniels, A. D.; Farkas, O.; Foresman, J. B.; Ortiz, J. V.; Cioslowski, J.; Fox, D. J.; Gaussian, Inc., Wallingford CT, **2013**.
6. a) J. P. Perdew, J. A. Chevary, S. H. Vosko, K. A. Jackson, M. R. Pederson, D. J. Singh, C. Fiolhais, *Phys. Rev. B* **1992**, *46*, 6671; b) A. D. Becke, *J. Chem. Phys.* **1993**, *98*, 5648.
7. (a) P. C. Hariharan, J. A. Pople, *Theor. Chem. Acc.*, **1973**, 28, 213. (b) W. J. Hehre, R. Ditchfield and J. A. Pople, *J. Chem. Phys.*, **1972**, 56, 2257
8. (a) A. E. Reed and F. J. Weinhold, *Chem. Phys.*, **1983**, 78, 4066; (b) A. E. Reed, L. A. Curtiss and F. Weinhold, *Chem. Rev.*, **1988**, 88, 899.
